# 3D Pharmacophore-Based Discovery of Novel K_V_10.1 Inhibitors with Antiproliferative Activity

**DOI:** 10.3390/cancers13061244

**Published:** 2021-03-12

**Authors:** Žan Toplak, Louise Antonia Hendrickx, Špela Gubič, Štefan Možina, Bojana Žegura, Alja Štern, Matjaž Novak, Xiaoyi Shi, Steve Peigneur, Jan Tytgat, Tihomir Tomašič, Luis A. Pardo, Lucija Peterlin Mašič

**Affiliations:** 1 Faculty of Pharmacy, University of Ljubljana, Aškerčeva 7, 1000 Ljubljana, Slovenia; zan.toplak@ffa.uni-lj.si (Ž.T.); spela.gubic@ffa.uni-lj.si (Š.G.); stefan.mozina@ffa.uni-lj.si (Š.M.); tihomir.tomasic@ffa.uni-lj.si (T.T.); 2 Toxicology and Pharmacology, Campus Gasthuisberg, University of Leuven, Onderwijs en Navorsing 2, Herestraat 49, P.O. Box 922, 3000 Leuven, Belgium; louise.hendrickx@kuleuven.be (L.A.H.); steve.peigneur@kuleuven.be (S.P.); jan.tytgat@kuleuven.be (J.T.); 3 Department of Genetic Toxicology and Cancer Biology, National Institute of Biology, Večna pot 111, 1000 Ljubljana, Slovenia; bojana.zegura@nib.si (B.Ž.); alja.stern@nib.si (A.Š.); matjaz.novak@nib.si (M.N.); 4 University of Ljubljana, Kongresni trg 12, 1000 Ljubljana, Slovenia; 5 AG Oncophysiology, Max-Planck Institute for Experimental Medicine, Hermann-Rein-Str. 3, 37075 Göttingen, Germany; shi@em.mpg.de

**Keywords:** K_V_10.1, ion channels, hERG, pharmacophore modelling, virtual screening, antiproliferative activity

## Abstract

**Simple Summary:**

A novel structural class of inhibitors of the voltage-gated potassium channel K_V_10.1 was discovered by a ligand-based drug design method using a 3D pharmacophore model. The virtual screening hit compound ZVS-08 inhibited the channel in a voltage-dependent manner consistent with the action of a gating modifier. Structure–activity relationship studies revealed a nanomolar K_V_10.1 inhibitor that is selective for some K_V_ and Na_V_ channels but exhibits significant inhibition of the hERG channel. K_V_10.1 inhibitor 1 inhibited the growth of the MCF-7 cell line expressing high levels of K_V_10.1 and low levels of hERG more potently than the Panc1 cell line (no K_V_10.1 and high hERG expression). Moreover, the K_V_10.1 inhibitor 1 induced significant apoptosis in tumour spheroids of Colo-357 cells. This study may provide a basis for the use of computational drug design methods for the discovery of novel K_V_10.1 inhibitors as new promising anticancer drugs.

**Abstract:**

(1) Background: The voltage-gated potassium channel K_V_10.1 (Eag1) is considered a near- universal tumour marker and represents a promising new target for the discovery of novel anticancer drugs. (2) Methods: We utilized the ligand-based drug discovery methodology using 3D pharmacophore modelling and medicinal chemistry approaches to prepare a novel structural class of K_V_10.1 inhibitors. Whole-cell patch clamp experiments were used to investigate potency, selectivity, kinetics and mode of inhibition. Anticancer activity was determined using 2D and 3D cell-based models. (3) Results: The virtual screening hit compound ZVS-08 discovered by 3D pharmacophore modelling exhibited an IC_50_ value of 3.70 µM against K_V_10.1 and inhibited the channel in a voltage-dependent manner consistent with the action of a gating modifier. Structural optimization resulted in the most potent K_V_10.1 inhibitor of the series with an IC_50_ value of 740 nM, which was potent on the MCF-7 cell line expressing high K_V_10.1 levels and low hERG levels, induced significant apoptosis in tumour spheroids of Colo-357 cells and was not mutagenic. (4) Conclusions: Computational ligand-based drug design methods can be successful in the discovery of new potent K_V_10.1 inhibitors. The main problem in the field of K_V_10.1 inhibitors remains selectivity against the hERG channel, which needs to be addressed in the future also with target-based drug design methods.

## 1. Introduction

**Figure 1:**
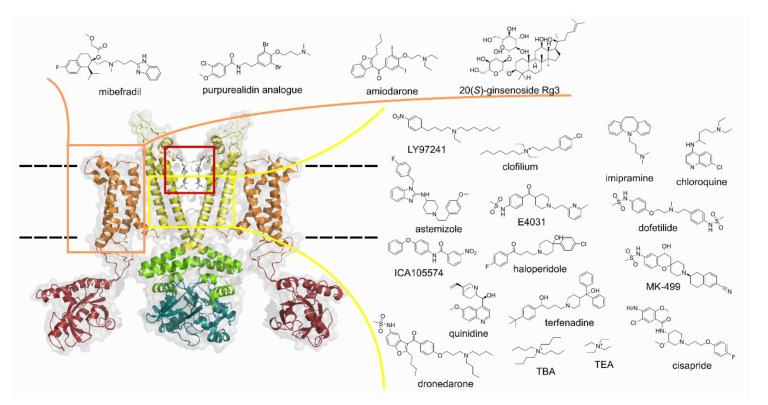
Opposite two subunits of K_V_10.1 with voltage-sensor domain (VSD) in orange, pore domain and central cavity in yellow, selectivity filter in red box. Structures of known ligands binding to the VSD and the central cavity of the pore domain. The structure (PDB: 5k7l) in the figure represents Kv10.1 with the pore domain in the closed conformation due to calmodulin binding and VSD in the upward/conductive conformation. TBA: tetrabutylammonium: TEA: tetraethylammonium. The structure of K_V_10.1 was prepared using PyMOL [21].

The voltage-gated potassium channel K_V_10.1 (Eag1) is a member of the ether-à-go-go family, which also includes the erg (eag-related gene, K_V_11) and elk (eag-like K^+^ channel, K_V_12) subfamilies [1]. Like other K_V_ channels, K_V_10.1 is a tetramer with a transmembrane region consisting of a voltage-sensor domain (VSD) with four alpha-helical segments (S1–S4) and segments S5 and S6 forming the ion-conducting pore (Figure 1). Positively charged residues in the VSD respond to membrane depolarisation by a movement that translates into S5 and S6 helices opening the pore to K^+^ flux that drives the membrane voltage toward the negative K^+^ equilibrium potential [2].

In healthy tissues, K_V_10.1 is almost undetectable outside the central nervous system, although it is highly expressed in over 70% of human cancers, regardless of tumour type [3]. On this basis, K_V_10.1 is considered a nearly universal tumour marker and represents a promising new target for new anticancer drug discovery [4]. The channel is involved in cell cycle control and cell proliferation, migration, angiogenesis and resistance to hypoxia of cancerous cells [5]. The mechanisms of cancer progression are complex with changes in membrane potential control and non-canonical effects of overexpressed K_V_10.1 that impact the Ca^2+^ signalling and microtubule dynamics [6]. In vitro and in vivo animal experiments showed that inhibiting the K_V_10.1 channel leads to the reduction of tumour progression [7,8,9,10,11]. Therefore, inhibition of the K_V_10.1 could increase the survival of patients with the fibrosarcoma [12], ovary carcinoma [13], glioblastoma [14], acute lymphoid leukaemia [15], gastric [16], head and neck [17] and colon cancers [18], all entities where a correlation between K_V_10.1 and poorer outcome has been documented. Importantly, treatment with K_V_10.1 inhibitors of brain metastasis patients improved survival in a retrospective study [14]. However, to date, no K_V_10.1-specific small-molecule inhibitors have been reported, as most of them also inhibit voltage-gated sodium channels or, most importantly, the cardiac hERG channel, thus posing the risk of cardiac side effects [4]. Recent studies have pointed to a particular structural difference between K_V_10.1 and hERG channels, suggesting the possibility of selective targeting of K_V_10.1 in cancer therapies [19]. This now opens exciting possibilities for further exploration using the published cryo-electron microscopy structures of K_V_10.1 and hERG [2,20].

To date, only a handful of different small molecules have been reported to inhibit K_V_10.1 (Figure 1). The identification of K_V_10.1 inhibitors is mostly based on the already known inhibitory activity at the structurally similar hERG channel and/or on the anti-tumour properties of the small molecules. The anti-tumour activity of natural products has led to the discovery of their blocking of Kv10.1, but to our knowledge, no other ligand- or structure-based drug design approach has been used to discover new K_V_10.1 inhibitors.

Due to the high similarity between K_V_10.1 and hERG, particularly in the putative drug binding sites in the central cavities of the channels, achieving selective inhibition of K_V_10.1 with pore-blocking small molecules presents a difficult hurdle. A VSD modulation approach could potentially circumvent this problem [20]. To date, only four types of small molecules are known to bind to the VSD, namely, mibefradil, natural bromotyrosine purpurealidin I analogues, amiodarone and 20(*S*)-ginsenoside Rg3 (Figure 1). The exact binding sites of these inhibitors are still unknown, but recently published cryo-electron microscopy structures of both K_V_10.1 and hERG offer the possibility of using the VSD as a binding region for new modulators [2,20,22,23]. In this work, we describe the ligand-based drug discovery of a new structural class of K_V_10.1 inhibitors using 3D pharmacophore modelling, structural optimisation of the virtual screening hit towards potent nanomolar K_V_10.1 inhibitors, selectivity profiling against hERG and some selected ion channels, mutagenicity study, and antiproliferative activity in 2D and 3D cell based models.

## 2. Materials and Methods

### 2.1. Virtual Compound Library Preparation

Three compound libraries were prepared: (1) a selected set of purpurealidin analogues as K_V_10.1 active compounds, (2) a calculated set of decoy molecules, and (3) a merged set of commercially available compounds available for hit identification via virtual screening with prioritised ligand-based pharmacophore model. For the K_V_10.1 actives library, structures of 11 purpurealidin I analogues were retrieved from a scientific publication of Moreels et al. [22]. A second library of 551 decoy molecules was generated based on each of the 11 K_V_10.1 inhibitors by submitting their structures to the DUDE decoy online generator [24], which resulted in 50 decoy molecules per compound with similar 1D physicochemical properties but dissimilar 2D topology in comparison to the 11 active compounds.

A third library of 556,000 compounds from commercial providers was prepared based on the diversity sets from Asinex, ChemBridge, Enamine, KeyOrganics, LifeChemicals, Maybridge, and Vitas-M. Libraries were downloaded in SDF format, merged, and duplicates removed using the LigandScout Database Merger and Duplicates Remover nodes as implemented in the Inte:Ligand Expert KNIME Extensions [25].

For each of the three libraries, a maximum of 200 conformations were generated for each molecule using LigandScout’s iCon algorithm [26,27] with the default “BEST” settings (Max. number of conformers per molecules: 200, Timeout (s): 600, RMS threshold: 0.8, energy window: 20.0, max. pool size: 4000, max. fragment build time: 30). Each library was saved in LDB (LigandScout database format) using LigandScout’s idbgen algorithm with default settings.

### 2.2. Ligand-Based Pharmacophore Modelling

Eleven purpurealidin-based K_V_10.1 inhibitors were used for creation of ten ligand-based pharmacophore models in LigandScout 4.3 Expert. The models were generated using the following ligand-based pharmacophore creation settings: Scoring function: pharmacophore fit and atom overlap; Pharmacophore type: Merged feature pharmacophore; Number of omitted features for merged pharmacophore: 4; partially matching features optional, threshold (%): 10.0; Feature tolerance scale factor: 1.0; Maximum number of result pharmacophores: 10. Creation of an exclusion volumes coat around the alignment of the ligands was also enabled for each of the models. All ligands of the training set were automatically aligned to the generated pharmacophore models. The resulting ligand-based pharmacophore models were tested for their performance in the distinguishing the active and decoy molecules. The best performing model was selected for virtual screening to identify new K_V_10.1 inhibitors.

### 2.3. Virtual Screening

Best final ligand-based pharmacophore model was used for virtual screening of the library of 556,000 commercially available compounds. The following settings were used for virtual screening: Scoring function: Pharmacophore-Fit; Screening mode: Match all query features; Retrieval mode: Get best matching conformation; Max. number of omitted features: 0, Check exclusion volumes: Checked. The virtual screening identified 18 virtual screening hits that were then visually inspected and nine of them where selected and purchased for in vitro screening against Kv10.1 using whole-cell patch clamp.

### 2.4. Chemistry

**Figure 2:**
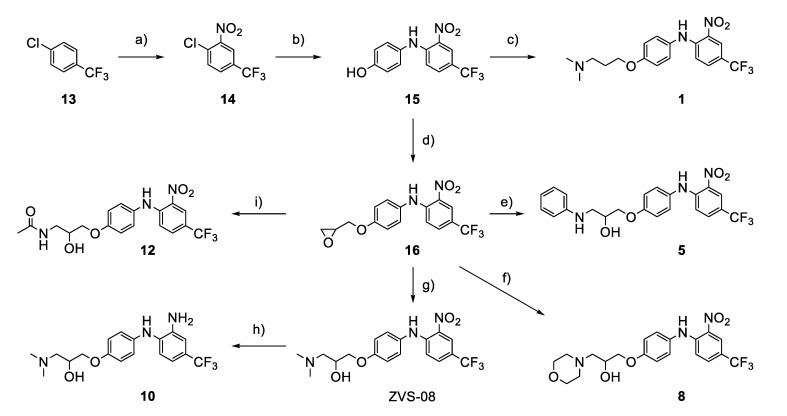
Reagents, solvents and conditions: (**a**) H_2_SO_4_, HNO_3_, 80 °C, 1 week; (**b**) 4-aminophenol, isopropanol, 80 °C, 40 h; (**b**) 4-aminophenol, NaHCO_3_, EtOH, 100 °C, 20 h; (**c**) 3-dimethylamino-1-propyl chloride hydrochloride, Cs_2_CO_3_, DMF, 100 °C, 3 h; (**d**) (±)-epichlorohydrin, MeCN, K_2_CO_3_, 80 °C, 16 h; (**e**) aniline, MeOH, r.t., 40 h; (**f**) morpholine, MeOH, r.t., 36–48 h; (**g**) Me_2_NH × HCl, MeOH, 70 °C, 24 h; (**h**) 20% wt Pd/C, H_2_, MeOH, r.t., 2 h; (**i**) BF_3_OEt_2_, MeCN, 80 °C, 16 h.

**Figure 3:**
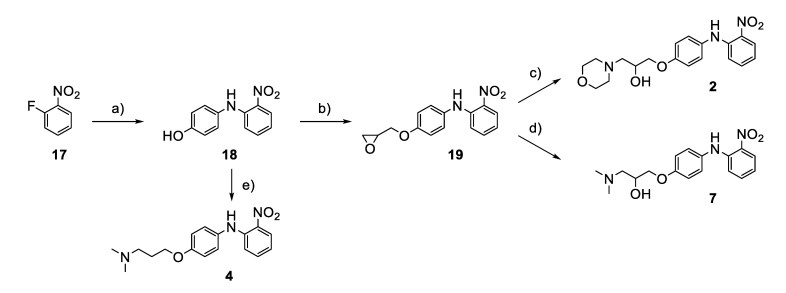
Reagents, solvents and conditions: (**a**) 4-aminophenol, NaHCO_3_, EtOH, 100 °C, 20 h; (**b**) (±)-epichlorohydrin, MeCN, K_2_CO_3_, 80 °C, 16 h; (**c**) morpholine, MeOH, r.t., 36–48 h; (**d**) Me_2_NH × HCl, MeOH, 70 °C, 24 h; (**e**) 3-dimethylamino-1-propyl chloride hydrochloride, Cs_2_CO_3_, DMF, 100 °C, 3 h.

**Figure 4:**
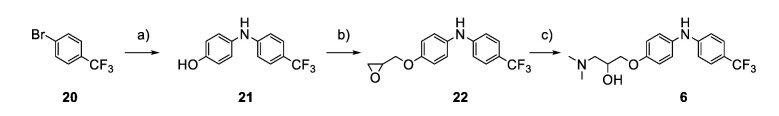
Reagents, solvents, and conditions: (**a**) 4-aminophenol, BrettPhos, Pd(dba_3_)_2_, NaO*t*-Bu, 90 °C, 12 h; (**b**) (±)-epichlorohydrin, MeCN, K_2_CO_3_, 80 °C, 16 h; (**c**) Me_2_NH × HCl, MeOH, 70 °C, 24 h.

**Figure 5:**
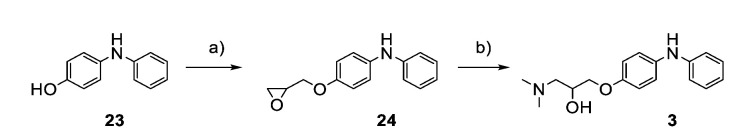
Reagents, solvents, and conditions: (**a**) (±)-epichlorohydrin, MeCN, K_2_CO_3_, 80 °C, 16 h; (**b**) Me_2_NH × HCl, MeOH, 70 °C, 24 h.

**Figure 6:**
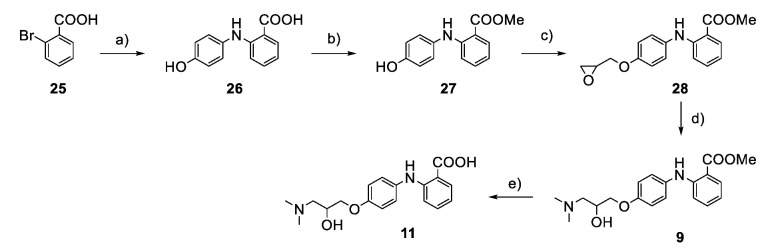
Reagents, solvents, and conditions: (**a**) 4-aminophenol, K_2_CO_3_, CuO, Cu, 2-ethoxyethanol, 130 °C, 16 h; (**b**) thionyl chloride, anhydrous MeOH, 70 °C, overnight; (**c**) (±)-epichlorohydrin, MeCN, K_2_CO_3_, 80 °C, 16 h; (**d**) NaOH, MeOH, water, r.t., 12 h.

The synthesis of the virtual screening hit ZVS-08 and its analogues is shown in Figure 2, Figure 3, Figure 4, Figure 5 and Figure 6. Synthesis of differently substituted 4-(phenylamino)phenols **15**, **18**, **21** and **26** was carried out using four different approaches. *o-*Nitro-*p-*trifluoromethyl-substituted phenol **15** was prepared in two steps (Figure 2). First, the nitro group was introduced by nitration of **13** with concentrated nitric and sulphuric acid to give **14**, followed by the addition of *p*-aminophenol at high temperature to yield **15** [28]. The nitro-substituted compound **18** was prepared by coupling *p*-aminophenol and **17** with the addition of base at reflux (Figure 3). For the synthesis of compounds without the electron-withdrawing nitro group, palladium-catalysed amination with Pd(dba_3_)_2_ was used for compound **21** (Figure 4), while CuO and Cu were used as catalysts for the synthesis of **26** (Figure 6) [29]. The carboxylic acid **26** was converted to the methyl ester **27** using thionyl chloride in anhydrous MeOH (Figure 6). Ethers **1** (Figure 2) and **4** (Figure 3) were formed from **15** and **18** using 3-dimethylamino-1-propyl chloride hydrochloride and Cs_2_CO_3_ as base. The preparation of epoxides **16**, **19**, **22**, **24** and **28** was carried out using (±)-epichlorohydrin and K_2_CO_3_ as base [30]. The synthesis of **12** by Ritter reaction (Figure 2) was carried out using acetonitrile in the presence of BF_3_OEt_2_ [31]. For epoxide opening, morpholine was used to give **2** and **8**; aniline was used to yield compound **5** and dimethylamine for compounds ZVS-08, **3**, **6**, **7** and **9**. Reduction of nitro group of ZVS-08 by catalytic hydrogenation gave compound **10** (Figure 2). The methyl ester of **9** was hydrolysed with 1 M sodium hydroxide to give compound **11** (Figure 6). Synthetic procedures, analytical data, chemicals, reagents, and equipment used for organic synthesis and analysis are written in Supplementary Materials under the Chemistry section.

### 2.5. Electrophysiological Recordings

Stage V-VI oocytes were isolated by partial ovariectomy from *Xenopus laevis* frogs (African clawed frog). Mature female frogs were purchased from CRB Xénopes (Rennes, France) and were housed in the Aquatic Facility (KU Leuven) in compliance with the regulations of the European Union (EU) concerning the welfare of laboratory animals as declared in Directive 2010/63/EU. The use of *Xenopus laevis* was approved by the Animal Ethics Committee of the KU Leuven (Project nr. P186/2019). After anesthetising the frogs by a 15-min submersion in 0.1% tricaine methanesulphonate (pH 7.0), the oocytes were collected. The isolated oocytes were then washed with a 1.5 mg/mL collagenase solution for 2 h to remove the follicle layer.

Ion channels (K_V_1.x, K_V_2.1, K_V_4.2, Na_V_1.4, Na_V_1.5) were expressed in *Xenopus laevis* oocytes, by linearisation of the plasmids and subsequent in vitro transcription using a commercial T7 or SP6 mMESSAGE mMACHINE transcription kit (Ambion, Carlsbad, CA, USA). Defolliculated *Xenopus* oocytes were then injected with 20–50 nL of the cRNA at a concentration of 1 ng/nL using a micro-injector (Drummond Scientific1, Broomall, PA, USA). The oocytes were incubated in a solution containing (in mM): NaCl, 96; KCl, 2; CaCl_2_, 1.8; MgCl_2_, 2 and HEPES, 5 (pH 7.4), supplemented with 50 mg/L gentamycin sulphate and 90 mg theophylline. After ex vivo translation, the ion channels are correctly inserted in the cell membrane of the oocytes.

Two-electrode voltage-clamp recordings were performed at room temperature (18–22 °C) using a Geneclamp 500 amplifier (Molecular Devices, San Jose, CA, USA) controlled by a pClamp data acquisition system (Axon Instruments1, Union City, CA, USA). Whole cell currents from oocytes were recorded 1–7 days after injection. The bath solution composition was either ND96 (in mM): NaCl, 96; KCl, 2; CaCl_2_, 1.8; MgCl_2_, 2 and HEPES, 5 (pH 7.5) or calcium- free ND96 supplemented with 10 mM BaCl_2_. Voltage and current electrodes were filled with a 3 M solution of KCl in H_2_O. Resistances of both electrodes were kept between 0.5 and 1.5 MΩ.

The elicited K_v_1.x, K_v_2.1, and K_v_4.2 currents were filtered at 0.5 kHz and sampled at 2 kHz, Na_V_1.4, and Na_v_1.5 currents were filtered at 1 kHz and sampled at 20 kHz using a four-pole low-pass Bessel filter. Leak subtraction was performed using a -P/4 protocol. For the electrophysiological analysis of the compounds, a number of protocols were applied from a holding potential of −90 mV. Currents for K_V_1.x, K_V_2.1, and K_V_4.2 were evoked by 0.5 s depolarising pulses to 0 mV, followed by 0.5 s repolarising pulses to −50 mV. Currents for Na_V_1.4 and Na_V_1.5 were evoked by 100 ms depolarising pulses to 0 mV.

Whole-cell patch clamp experiments were performed on stably transfected HEK-293 cells [32,33]. The cells were grown on fibronectin-coated glass coverslips in DMEM supplemented with 10% FCS and 300 µg/mL Zeocin. Experiments were performed at room temperature under continuous perfusion. The extracellular solution contained (mM) 160 NaCl, 2.5 KCl, 2 CaCl_2_, 1 MgCl_2_, 10 Hepes/NaOH, pH 7.4, 8 glucose. For the experiments determining conductance–voltage relationships, tail currents were made visible by substituting 60 mM NaCl by KCl (162.5 mM KCl, 100 NaCl). Pipettes were pulled from #1 glass capillaries (WPI) to resistances of 1–3 MΩ when loaded with the intracellular solution (100 KCl, 45 *N*-methyl-D-glucamine, 10 1,2-bis(*o*-aminophenoxy)ethane-*N*,*N*,*N′*,*N′*-tetraacetic acid (BAPTA), 10 Hepes pH 7.35). Data acquisition was performed using a EPC10 Plus amplifier (HEKA Elektronik) and Patch Master software. Drug application was performed using an 8-channel fast solution exchanger (ALA). All compounds were dissolved in DMSO to a final concentration of 10 mM. 0.5% DMSO in extracellular solution served as control. K_V_10.1 currents were elicited by 500 ms depolarisations to +40 mV under continuous perfusion with the compound-containing or control solution. hERG current amplitudes were measured as the peak tail current during a 4 s repolarization to −40 mV after a depolarising pulse to +20 mV lasting for 2 s.

### 2.6. Statistical Analysis

All electrophysiological data are presented as means ± SEM of *n* ≥ 3 independent experiments unless otherwise indicated. Oocyte data were analysed using pClamp Clampfit 10.4 (Molecular Devices, Downingtown, PA, USA) and OriginPro 9 (Originlab, Northampton, MA, USA) or GraphPad Prism 8 software (GraphPad Software, Inc., San Diego, CA, USA). Patch-clamp data analysis was performed using FitMaster (HEKA Electronics (Ludwigshafen, Germany)), IgorPro (Wavemetrics, Inc, Lake Oswego, OR, USA) and Prism v.8. The Dunnett test and one-way ANOVA were performed to calculate the significance of the induced inhibition compared to the control (*p* < 0.05).

### 2.7. Cell Culture

Cell lines MCF-7 (DSMZ ACC 115), PANC-1 (DSMZ ACC 783), and hTERT-RPE1 (ATCC CRL 4000) were grown in the media recommended by the cell provider in a humidified atmosphere at 5% CO_2_ and 37 °C. For cell growth experiments, 10^4^ cells/well were seeded in 96-well flat bottom culture plates (Corning) and allowed to grow for 24 h. The compounds were then added in the corresponding growth medium, and the plate was imaged every hour for 72 h (two images per well and eight wells per condition in two independent experiments for compound **8** and five for ZVS-08 and compound **1**) in an Incucyte system (Sartorius/Essen biosciences). Culture confluence was determined in the phase contrast images and is expressed as percent confluence per well. The growth was then normalised to the value in the absence of compounds.

Spheroids were cultured in round bottom ultra-low attachment 96-well plates (Corning) at a density of 5000 cells/well in 2% Matrigel (Corning) and centrifuged at 1000× *g* for 10 min. Spheroid formation was monitored continuously in the Incucyte system and treatments were added once spheroids were formed. As apoptosis sensitizer, 8.9 µM cycloheximide was added to controls and treated spheroids. Caspase 3/7 green reagent (Sartorius; 1:1000) was used as recommended by the manufacturer. Caspase 3/7 reagent substrate crosses the cell membrane and its cleavage by activated caspase-3/7 results in the release of a DNA dye and fluorescent staining of nuclear DNA.

### 2.8. Evaluation of Mutagenic Activity with Salmonella/Microsomal Reverse Mutation Assay

Mutagenicity of compounds ZVS-08 and **8** was tested with the Salmonella/microsomal reverse mutation (Ames) assay on *Salmonella typhimurium* strains TA98 and TA100 without and with external metabolic activation according to the OECD guidelines (OECD TG 471) with minor modifications. Briefly, 100 µL of ZVS-08 and 8 (0.158, 0.5, 1.58, 5, 15.8, and 50 mg/mL corresponding to the final concentration of 0.0158, 0.05, 0.158, 0.5, 1.58, and 5 mg/plate, respectively), 100 mL bacterial culture of *S. typhimurium* (overnight culture at 37 °C), and 500 mL of phosphate buffer (for assays without metabolic activation) or 10% S9 mix (for assays with metabolic activation) were added to 2 mL of molten top agar (supplemented with histidine/biotin, final concentration 0.05 mM), gently mixed and poured onto minimal agar plates. The number of His+ revertants was counted after 48 h (TA100) and 72 h (TA98) of incubation at 37 °C. Three plates were used per treatment point. The mutagenic potential of the compounds was expressed as an induction factor (IF), where IF = (number of revertants in the presence of the compound)/(number of revertants in solvent control). Additionally, each plate was checked for possible toxic effects of tested compounds. The concentration is considered as cytotoxic when the microcolony lawn is reduced and is classified as moderately (distinguished by a marked thinning of the bacterial lawn that may result in a pronounced increase in the size of microcolonies compared to the solvent control plate), extremely reduced (distinguished by an extreme thinning of the bacterial background lawn that may result in an increase in the size of the microcolonies compared to the solvent control plate such that the microcolony lawn is visible to the unaided eye as isolated colonies) or absent (distinguished by a complete lack of any bacterial background lawn over greater than or equal to 90% of the plate). 4-nitroquinoline- N-oxide (4-NQNO; 0.25 μg/plate) for TA98 and sodium azide (NaN*_3_*; 0.25 μg/plate) for TA100 were used as positive controls without S9 metabolic activation, while benzo[*a*]pyrene (B[a]P; 2.5 μg/plate) was used as positive controls for both bacterial strains with S9 metabolic activation.

## 3. Results

### 3.1. Ligand-Based Identification of Novel K_V_10.1 Inhibitors through in Silico Screening

The discovery of K_V_10.1 inhibitors has been limited to reports of known compounds or drugs that bind to this channel mostly as an off-target (Figure 1). Even though the three-dimensional structure of K_V_10.1 has been determined by cryo-electron microscopy [2], the actual binding sites of small molecules in K_V_10.1 remain mostly unknown. Therefore, structure-based design and virtual screening are rarely used to identify new K_V_10.1 inhibitors. Limitations of structure-based in silico approaches in the design of K_V_10.1 inhibitors can be overcome by ligand-based methods, such as 3D similarity search and pharmacophore modelling.

The latter method can be used to build ligand-based pharmacophore models (LBPM) based on a set of active ligands. The pharmacophore model consists of a set of interactions aligned in 3D space and is represented by a collection of pharmacophore features with defined properties, such as aromatic ring, hydrogen bond donors and acceptors, hydrophobic, negatively and positively charged groups, and halogen bonds features. The known active compounds are defined as a training set and aligned in 3D space, followed by the generation of a pharmacophore model.

One of the most important requirements for generating ligand-based pharmacophore models is that the ligands have the same mode of action and bind in the same orientation in the binding site. Therefore, to generate and validate the LBPM, we used a set of 11 purpurealidin I analogues that showed K_V_10.1 inhibition [22]. This is the only series of analogues that was evaluated for inhibition of K_V_10.1. They also displayed antiproliferative effects in cancer cell lines with high K_V_10.1 expression. Based on competition experiments with mibefradil, a suggested binding site for these compounds is in the VSD of K_V_10.1, although its exact location remains to be elucidated experimentally [22]. The LBPM method circumvents this limitation as the model is based solely on the alignment of the ligands in the training set. Due to the similarity between the ligands, we assumed that the binding site is shared among the active purpurealidin analogues, which is necessary for a valid LBPM generation. The higher diversity between K_V_10.1 and hERG channels in the VSD than in the pore of the channels highlights purpurealidin analogues as excellent starting points for identifying novel K_V_10.1 inhibitors [34].

In virtual screening using LBPM as a query, the ligands in the screening library are clustered into active and decoy (inactive) datasets, and then, their pharmacophore fit scores are calculated and ranked in the hitlist from highest to lowest score. Potentially active compounds have high pharmacophore fit scores, while decoys poorly fit the pharmacophore model and receive low scores. Screening results can be visualised by Receiver Operating Characteristic (ROC) plots, where the rate of true positives (active compounds) is on the *y*-axis and the rate of false positives (decoys) is on the *x*-axis. If the ROC plot curve follows the diagonal line, it means that the model is performing poorly and cannot distinguish between decoy and active compounds. A ROC curve plotted above the diagonal represents a pharmacophore model that can detect active compounds. In addition, the enrichment factor (EF) represents the number of active compounds identified by a query pharmacophore model, as opposed to the number of compounds hypothetically found to be active when randomly screened. Thus, an enrichment factor of 1 means that the ligands are randomly sorted. In contrast, high enrichment factors mean that only a small fraction of the hitlist needs to be screened in vitro to identify a high number of active compounds.

**Figure 7:**
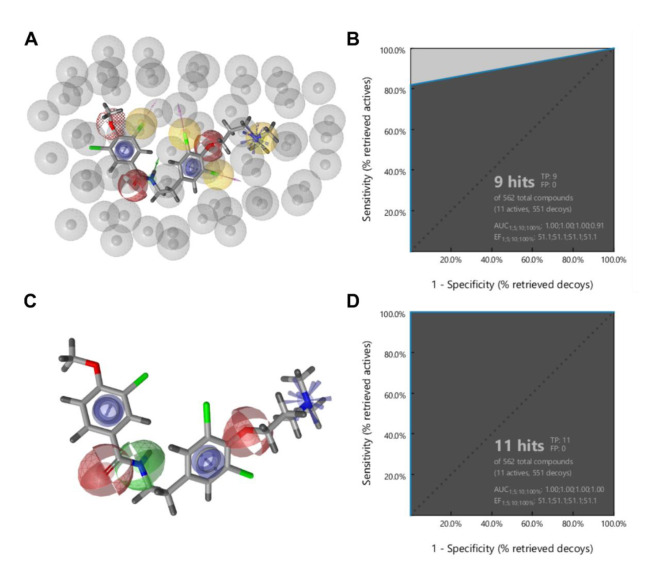
(**A**) Initial ligand-based pharmacophore model for K_V_10.1 inhibitors based on a library of purpurealidin analogues. Common chemical features of the aligned molecules include 4 hydrophobic features (yellow spheres), 3 hydrogen bond acceptors (red spheres), 1 hydrogen bond donor (a green arrow), 2 aromatic features (blue discs), 1 positive ionisable feature (blue star), 3 halogen bond features (pink arrows), and exclusion volumes (grey spheres) that define restricted regions based on the shape of the aligned molecules. Optional features are marked as dashed. (**C**) Final ligand-based pharmacophore model. Common chemical features of the aligned molecules include 2 hydrogen bond acceptors (red spheres), 1 hydrogen bond donor (a green sphere), 2 aromatic features (blue discs), and 1 positive ionisable feature (blue star). Grey spheres for exclusion volumes are not shown. Resulting ROC plot (curve shown in blue) for (**B**) initial and (**D**) final LBPM from virtually screening 562 compounds (11 K_V_10.1 active compounds and 551 generated decoys) with the ligand-based pharmacophore model. TP = true positives; FP = false positives; AUC = area under the curve; EF = enrichment factor.

To evaluate the performance of LBPM before running the real virtual screening campaign, it needs to be trained and validated. Ideally, the model will find a high number of active ligands from the training set and no ligands from the decoy set. Ligand-based pharmacophore modelling was performed in LigandScout 4.3 Expert [35,36]. Ten 3D LBPM with the best alignments were generated and scored with pharmacophore alignment scores. Exclusion volume features (Figure 7A, grey spheres) were automatically added to each model based on the shape of the ensemble of aligned molecules. These features represent restricted space and are important to increase the selectivity of a model for virtual screening campaigns.

An initial pharmacophore model was constructed based on the alignment of the 11 most potent purpurealidin-based K_V_10.1 inhibitors and is shown in Figure 7A [22]. It consisted of 14 pharmacophore features, 12 of which were essential (four hydrophobic features, marked as yellow spheres; two hydrogen bond acceptors, marked as red spheres; one hydrogen bond donor, marked as a green arrow; two aromatic features, marked as blue discs; one positive ionisable feature, marked as a blue star; and two halogen bond features, marked as pink arrows), and two were marked as optional (dashed red circle for one hydrogen bond acceptor and dashed pink arrow for one halogen bond). For each active compound in the dataset, 50 decoys were generated using the DUD-E server [24]. The DUD-E database calculates decoys based on the given set of K_V_10.1 inhibitors, which means that some of the decoys could still inhibit K_V_10.1 as they are similar to the active molecules. However, this is highly unlikely. This initial LBPM was tested with a library of active and decoy molecules. The performance of this model was very good in terms of the enrichment factor of 51.1, finding 9 out of 11 active compounds from the dataset and no decoy ligands (Figure 7B). However, it was too restrictive and performed poorly in virtual screening of commercially available compounds as no hits were identified. Therefore, our next step was to simplify the original LBPM while retaining its favourable screening properties, with the model able to distinguish between active and inactive compounds.

Through several rounds of simplification, we obtained our final LBPM, which was used for virtual screening. Specifically, we eliminated both optional pharmacophore features and removed halogen bond and hydrophobic features. In addition, we converted the directional hydrogen bond donor feature to a non-vector feature. Our final model (Figure 7C) consisted of six pharmacophore features: two aromatic, one hydrogen bond donor, two hydrogen bond acceptors, and one positive ionisable feature. The performance of the model in the validation screen was optimal as it was able to find all active ligands and no decoy compounds, resulting in a high enrichment factor of 51.1 (Figure 7D). When this model was used in the virtual screening campaign, it was able to identify 18 potentially active compounds from the library of 556,000 diverse commercially available compounds. The in silico hits were visually inspected and nine compounds (Table 1) were selected and purchased from the commercial vendors.

### 3.2. Patch-Clamp Screening of Virtual Screening Hit Compounds

**Figure 8:**
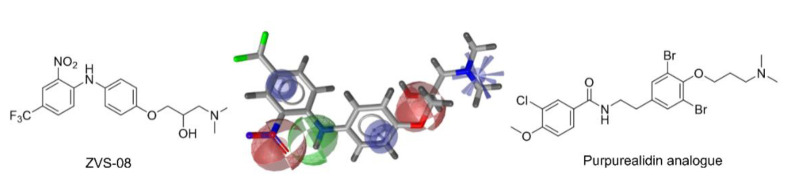
Alignment of the identified potent K_V_10.1 hit compound ZVS-08 with the final ligand-based pharmacophore model used for virtual screening (middle), structures of the most active purpurealidin analogue (right) and virtual screening hit compound ZVS-08 (left).

The nine selected in silico hits were tested by manual patch-clamp on a HEK293 cell line stably expressing K_V_10.1 [33] at a compound concentration of 50 μM (Table 1). Compound activity was tested by determining the current amplitude elicited by repetitive stimuli to +40 mV from a holding potential of −80 mV. The most potent inhibitor was hit compound ZVS-08 and after its resynthesis an IC_50_ value of 3.70 ± 1.01 μM was determined against K_V_10.1. The hit compound ZVS-08 represents a new structural class of K_V_10.1 inhibitors with diarylamine structure and has a unique structure among the acquired virtual screening hits. The alignment of ZVS-08 in the pharmacophore model used in the virtual screening shows that all pharmacophore features of the model are fulfilled (Figure 8). The most notable change from the purpurealidin analogues is the replacement of the hydrogen bond acceptor group of the amide bond with the oxygen atom of the nitro group of ZVS-08.

### 3.3. Characterisation of the K_V_10.1 Inhibition by ZVS-08

**Figure 9:**
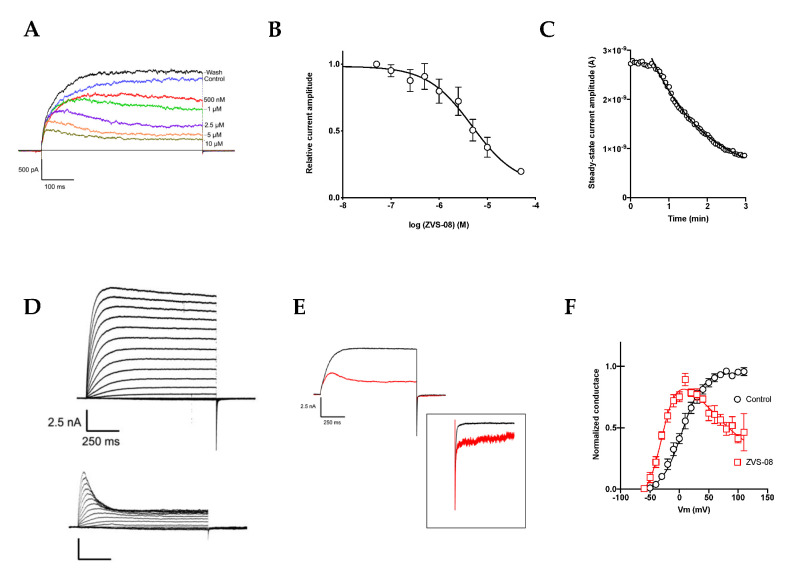
(**A**) Inhibition by different concentrations of ZVS-08 hit compound. (**B**) Dose–response (data from 3–10 independent determinations, mean ± SEM). (**C**,**D**) Current–voltage relationship in the absence (upper traces) or presence of 5 µM ZVS-08 (lower traces) obtained by depolarisation from a holding potential of −80 mV to potentials ranging between −60 and +100 mV. (**E**) The traces corresponding to depolarisation to +40 mV are superimposed for easier comparison. The inset shows the normalised tail currents to highlight the slower deactivation in the presence of ZVS-08. (**F**) Conductance–voltage relationship in control (black symbols) and in the presence (red symbols) of 5 µM ZVS-08, obtained by biexponential fit to the tail current (at −80 mV) and extrapolation to time 0. The solid lines represent the result of a fit using a Boltzmann distribution with a block at positive voltages.

The inhibition by ZVS-08 was characterised in more detail by patch clamp. Most hERG blockers bind to a hydrophobic cavity in the inner mouth of the channel pore [20], and some well-characterised compounds use the equivalent structure in K_V_10.1 (e.g., [32]) to interfere with ion permeation. The electrophysiological behaviour of our hit compound ZVS-08 was similar to that of astemizole, a well-established open-channel blocker of K_V_10.1 with an IC_50_ in the range of 200 nM [33]. The inhibition was concentration-dependent (Figure 9A,B), with an apparent IC_50_ of 3.70 µM (Table 2). The onset of inhibition was slow and required minutes of continued stimulation (Figure 9C), which would be compatible with an intracellular binding site. Moreover, the inhibition was not homogeneous during the stimulus. Current amplitude decreased along the stimulus in the presence of ZVS-08, resulting in apparent inactivation and smaller amplitude at the end of the pulse than in the beginning (Figure 9A,D). The effect was not cumulative, and the next stimulus (2.3 s later) showed a similar peak current and decay, indicating that the compound is not trapped in the closed channel. The apparent inactivation was more pronounced at more depolarised potentials (Figure 9D). Additionally, to the changes in inactivation of K_V_10.1, ZVS-08 also noticeably slowed down deactivation of the channel (Figure 9E), again indicating that the compound needs to unbind before the channels can deactivate. The inhibition was slightly voltage dependent (Figure 9C). In the presence of ZVS-08, the conductance–voltage relationship was well described by a Boltzmann function with a blocking component (Figure 9F). The activation was shifted by approximately −25 mV (semi-maximal activation at −29.88 ± 1.8 mV in the presence and 4.39 ± 1.9 mV in the absence of ZVS-08), compatible with the action of a gating modifier.

**Figure 10:**
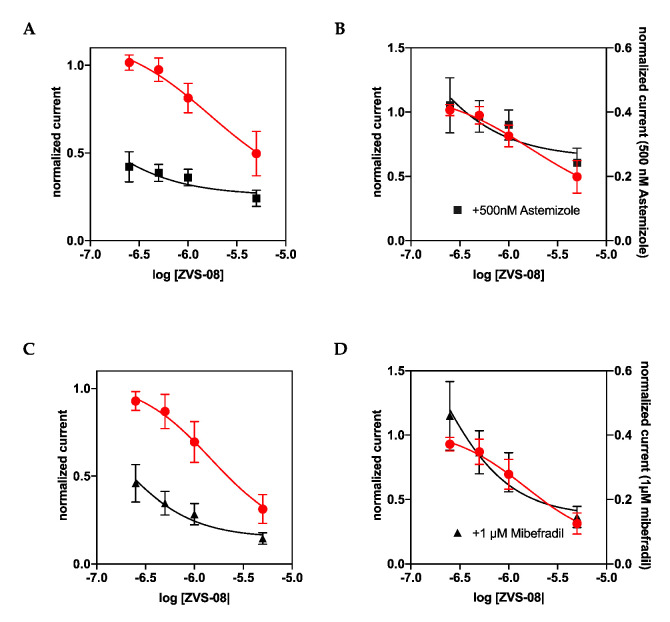
Competition results of ZVS-08 (red circles) with astemizole (known pore blocker, black square) on K_V_10.1 (**A**,**B**) and mibefradil (gating modifier, black triangle) (**C**,**D**). (**B**,**D**) represent the same dataset as (**A**,**C**), but each series is plotted on a different axis to visualise that the dose–response is unchanged in the presence of the competitor. The fits resulted in the same IC_50_ for the presence and absence of the competitor (1.69 µM for A and B and 1.47 µM for (**C**,**D**)). Current amplitudes were measured in all cases at the end of a 500 ms depolarising pulse to +40 mV.

To clarify the mechanism of inhibition by ZVS-08, we compared the compound to two well characterised inhibitors: astemizole, which behaves as an open channel blocker binding to the promiscuous intracellular hydrophobic pocket in the channel family, and mibefradil, which acts from the extracellular side and modifies channel gating. To test if ZVS-08 shares binding site with any of the two inhibitors, we performed competition experiments by determining if the presence of astemizole or mibefradil altered the effect of ZVS-08 on Kv10.1 current. The results are summarised in Figure 10. The degree of inhibition at the concentrations tested was independent of the presence of 500 nM astemizole or 1 µM mibefradil, indicating that the compounds do not compete for the same binding site.

### 3.4. Analogues of the Hit Compound ZVS-08 and Structure–Activity Relationships

**Figure 11:**
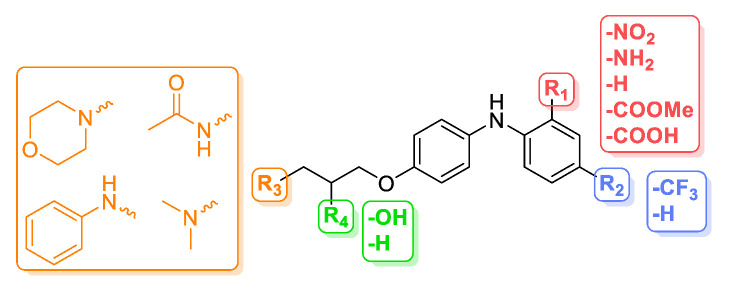
Design of the hit compound ZVS-08 analogues.

Analogues of the hit compound ZVS-08 were designed and synthesised to increase potency and selectivity and to investigate structure–activity relationships (SAR) important for K_V_10.1 inhibition. The hit compound ZVS-08 was modified at four different positions (Figure 11) to provide a focused library of compounds screened for K_V_10.1 (Table 3) and selected compounds for hERG inhibition (Table 2).

Modifications in the aromatic portion of ZVS-08 were performed on the phenyl ring bearing *ortho*-nitro and *para-*trifluoromethyl substituents. One or both groups were systematically removed in compounds **3**, **6** and **7** to analyse the influence of both substituents on the potency of K_V_10.1 inhibition. The nitro group was replaced by a methyl ester group in compound **9**, an amino group in compound **10**, and a carboxylic acid group in compound **11**, with the aim of removing the nitro group, which is sometimes associated with genotoxicity [37]. The basic dimethylamino group was replaced by four different amines (R_3_ in orange, Figure 11) to evaluate the importance of the cationic centre. Specifically, the basicity of the amine was reduced by introducing the aniline moiety (**5**) or removed by forming the acetamide (**12**). In addition, the aniline moiety in **5** was also introduced to assess whether the compound could be further extended in this part of the molecule and whether the formation of additional cation–π or π–π interactions was possible. The morpholino moiety in **2** and **8** was incorporated to maintain the basic character of the parent compound ZVS-08, but to further increase the polarity of the compound. The aliphatic chain between the basic centre and the aromatic ring of ZVS-08 (R_4_ in green, Figure 11) was also modified. The hydroxy group was removed to generate compounds **1** and **4**, which differ by the substitution of the aromatic ring.

**Figure 12:**
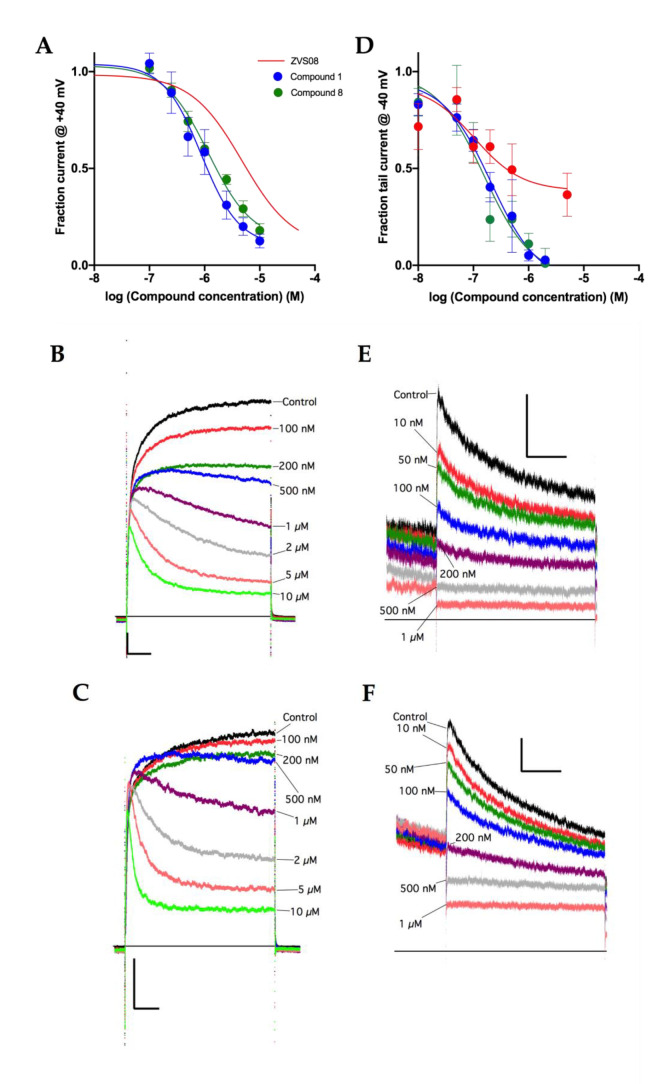
(**A**) Dose–response curves of compounds **1** and **8** for K_V_1.10 inhibition. The red trace has been included as reference and corresponds to ZVS-08 (from Figure 9B). The IC_50_ values from the fit were 740 nM (95% CI 490 nM to 1.17 µM, N = 7–9) for compound **1** and 1.01 µM (95% CI: 796 nM to 1.30 µM, N = 4–7) for compound **8**. (**B**) Representative Kv10.1 current traces obtained in the presence of different concentrations of Compound **1**. Scale bars: 1 nA, 50 ms. The cell was depolarized to +40 mV for 500 ms. No leak subtraction was performed. (**C**) Representative traces obtained in the presence of different concentrations of Compound **8**. Scale bars: 1 nA, 50 ms. (**D**) Dose–response curves of compounds ZVS-08, **1** and **8** for hERG inhibition. The IC_50_ values from the fit were 0.194 µM (95% CI 0.047 µM to 0.761 µM, N = 2–7) for ZVS-08, 0.207 µM (95% CI 0.113 µM to 0.395 µM, N = 3–8) for compound **1** and 0.156 µM (95% CI: 0.070 µM to 0.373 µM, N = 3–8) for compound 8. (**E**) Representative traces of hERG currents obtained in the presence of different concentrations of Compound **1**. Scale bars: 150 pA, 1 s. Only the end of the response at +20 mV and the tail current at −40 mV are represented. (**F**) Representative traces obtained in the presence of different concentrations of Compound **8**. Scale bars: 150 pA, 1 s.

Based on the study of structure–activity relationships of ZVS-08 analogues, we identified structural parts important for K_V_10.1 inhibition of this new structural type of K_V_10.1 inhibitors (Table 3). It is concluded that the two most potent K_V_10.1 inhibitors, **1** and **8**, contain a combination of disubstituted phenyl ring with both *ortho*-nitro and *para*-trifluoromethyl groups and basic moieties such as dimethylamino group in compound **1** and morpholino group in compound **8**. Both optimised compounds **1** and **8** exhibited improved potencies compared to the hit compound ZVS-08 with the IC_50_ values of 740 nM and 1.01 µM, respectively (Figure 12A, Table 2).

Disubstituted *ortho*-nitro and *para*-trifluoromethyl compounds with a weaker basic centre (**5**) or without it (**12**) were less potent. A similar effect of replacing the basic centre was observed with purpurealidin analogues [22]. The disubstituted compound in which the nitro group was replaced by the amino group in compound **10** was less potent. The unsubstituted compound **3** was almost inactive, indicating the importance of the disubstituted *ortho*-nitro and *para*-trifluoromethyl phenyl moiety in this structural type of K_V_10.1 inhibitors. Compounds **9** and **11**, in which the nitro group was exchanged with the methyl ester and carboxylic acid groups, were also inactive. The analogue of ZVS-08 without the nitro group and retaining the *para*-trifluoromethyl group (**6**) was also less potent. All three *ortho*-nitro compounds (**2**, **4** and **7**) without the *para*-trifluoromethyl group and with basic groups, showed almost the same inhibitory activity on K_V_10.1. Compounds **1** and **4** lack the hydroxyl group in the aliphatic part of the molecule, but this modification was not essential for the inhibitory potency against K_V_10.1. In general, the aliphatic amine plays an important role in binding.

Compounds ZVS-08, **1** and **8** were tested for selectivity on hERG with the patch-clamp assay (Figure 12B, Table 2). The selectivity of ZVS-08, **1** and **8** against the hERG channel remains an issue similar to the purpurealidin analogues [38]. However, with structural optimization, we increased the potency for K_V_10.1, while the potency for hERG remains the same for all three compounds ZVS-08, **1** and **8** (Table 2).

### 3.5. Selectivity of Compounds ZVS-08 and 1 against Other Voltage-Gated Ion Channels

**Figure 13:**
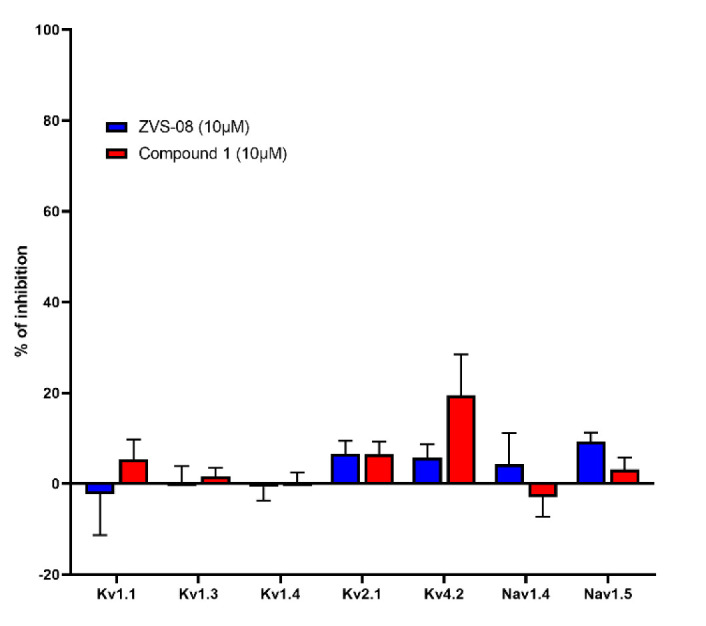
Selectivity screening of ZVS-08 and 1 against voltage-gated potassium and sodium channels at a concentration of 10 µM. Data were obtained with the two-electrode voltage clamp on *Xenopus laevis* oocytes. Data are presented as means ± SEM of *n* ≥ 3 independent experiments.

Selected potent K_V_10.1 inhibitors ZVS-08 and **1** were further evaluated for their selectivity against other potential targets in a panel of voltage-gated ion channels using the *Xenopus laevis* heterologous expression system, which allows efficient horizontal pharmacological comparison. Several different voltage-gated ion channels relevant in cardiac physiology, the immune system, and skeletal muscle were screened. Both K_V_10.1 inhibitors ZVS-08 and **1** showed no significant inhibition and were thus considered selective for the K_V_1.1, K_V_1.3, K_V_1.4, K_V_2.1, K_V_4.2, Na_V_1.4, and Na_V_1.5 ion channels at a concentration of 10 µM (Figure 13, Table S1). However, the selectivity should be further investigated to check the activity of these compounds on a broader range of ion channels.

### 3.6. Effects on the Growth of Cell Lines in 2D Cell Culture

**Figure 14:**
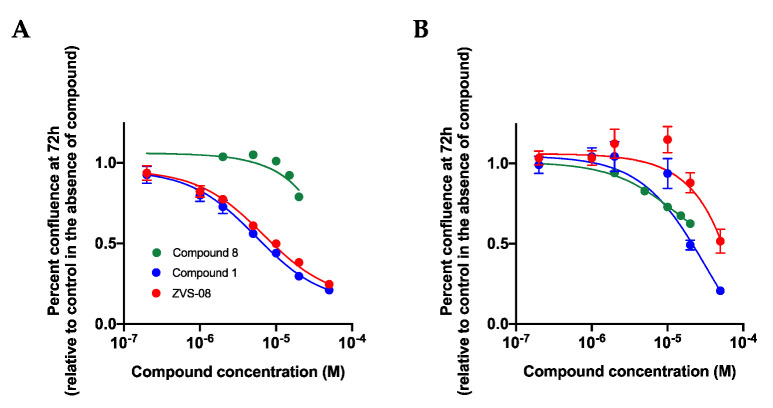
Effects of ZVS-08 and compounds **1** and **8** on the growth of MCF-7 (**A**) and Panc1 cells (**B**). ZVS-08 and compound **1** inhibited the growth of MCF-7 cells with IC_50_ of 6.8 (95% CI: 5.14–9.38 µM) and 5.48 µM (95% CI: 4.51–6.76 µM). Compound **8** was ineffective at the concentrations tested. None of the compounds inhibited the growth of Panc1 cells significantly at concentration below 10 µM, and an IC_50_ could only be determined for compound **1** (over 30 µM).

K_V_10.1 inhibitors with a favourable pharmacological profile are promising candidates for cancer therapy. To investigate the activity of the new compounds on cancer cells proliferation, compounds ZVS-08, **1** and **8** were tested on MCF-7, a breast cancer cell line showing high K_V_10.1 expression and low hERG expression, and on Panc1, a pancreatic cancer cell line with undetectable K_V_10.1 expression and high level of hERG expression. hTERT-RPE1 cells served as a non-cancer cell line. hTERT-RPE1 cells show transient physiological expression of Kv10.1 that begins during the G2 phase of the cell cycle and extends through mitosis as present in normal cells. Different concentrations of the compounds were added to the culture medium and the growth of the cell was monitored over 72 h (Figure 14).

ZVS-08 and **1** inhibited the growth of MCF-7 cells to a very similar extent and also with similar potency, with IC_50_ in the range of 5 µM. Compound **8** was inactive on this cell line, and all three compounds were significantly less efficient on Panc1; only the highest concentrations used showed effects. hTERT-RPE1 cells, a non-transformed cell line, showed a similar sensitivity to ZVS-08 and compound **1** as Panc1. In summary, ZVS-08 and **1** were able to reduce the growth of cancer cells expressing K_V_10.1 but were much less active on cells expressing hERG or on normal cells, suggesting that their effect on K_V_10.1 serves more to inhibit growth in these cells. The lack of activity of compound **8** in this assay could be caused by a higher affinity for serum proteins, depleting the compound in the medium, as has been repeatedly observed with many compounds [39].

### 3.7. 3D Cell Based Assays

To test the ability of the compounds to inhibit tumour progression in a more predictive system, we performed experiments on tumour spheroids of the pancreatic cancer cell line Colo357, which grows efficiently in this culture modality. The cells were seeded in the presence of Matrigel (see methods), allowed to form spheroids, and the compounds (50 µM) were added to the growth medium. The control contained the same amount of the vehicle (0.5% DMSO). Apoptosis was monitored by addition of a fluorescent caspase 2/7 activity reporter at the same time as the compounds. Cleavage of the reporter by caspase results in green florescence, and the intensity of the fluorescence was recorded over 72 h.

**Figure 15:**
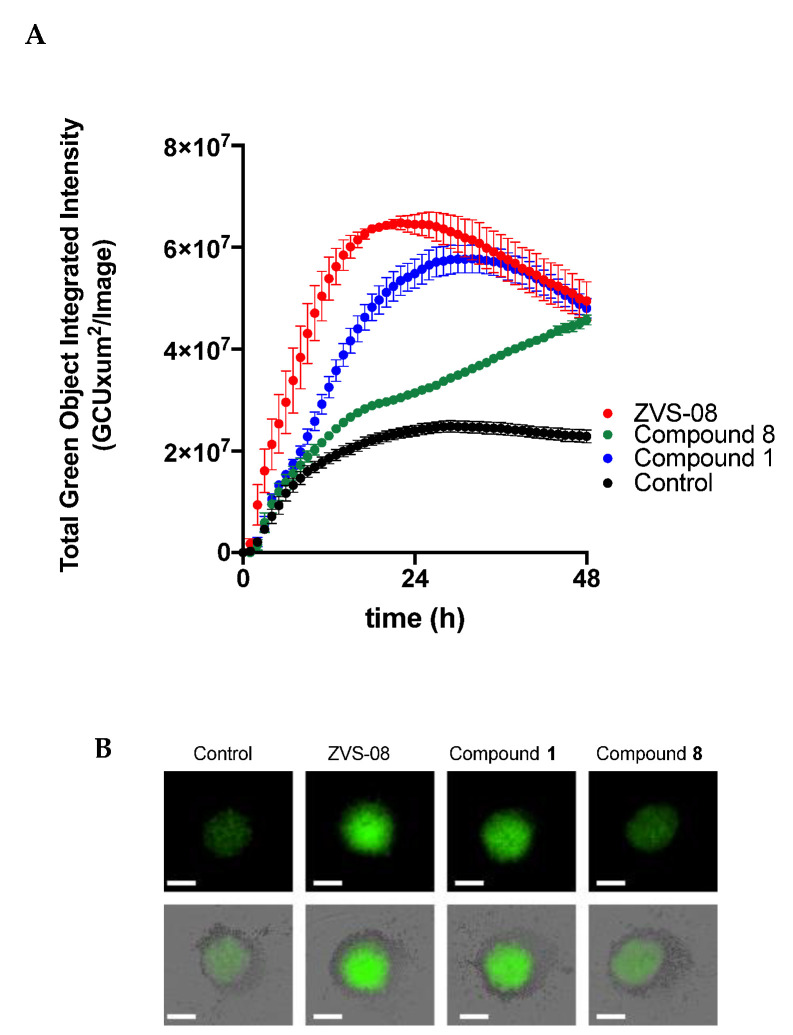
(**A**) Apoptosis induction by ZVS-08 and compounds **1** and **8** (50 µM) on tumour spheroids formed by Colo 357 cells. ZVS-08 was markedly faster and compound **8** was slowest. Data represent mean ± standard error (*N* = 3). (**B**) Representative images of spheroids 24 h after the addition of the indicated compounds (50 µM). Fluorescence intensity in the upper image corresponds to cleavage of the caspase 3/7 activity reporter; it has been merged with the phase contrast image in the lower raw. Scale bar: 400 µm.

As depicted in Figure 15, ZVS-08 induced apoptosis efficiently starting very early, while compound **1** required longer time, but reached similar efficacy. Compound **8** showed apoptosis statistically significant over the control starting only after 24 h and did not reach the same intensity as the other compounds in the time of the experiment. This lower efficacy matches the small effects detected in conventional 2D culture with this compound.

### 3.8. Mutagenic Activity of ZVS-08 and 8

The association of nitro groups with mutagenicity is well known in medicinal chemistry. For this reason, this is not the usual functional group among approved drugs, but recently, there have been advances in drug design incorporating the nitro group in antiparasitic, antitubercular, and anticancer compounds. There is a need to understand bioactivation potential of compounds bearing aromatic nitro groups [37]. The potential mutagenic activity of compounds ZVS-08 and **8** was determined using the bacterial *Salmonella*/microsomal reverse mutation assay with TA98 and TA100 *Salmonella typhimurium* strains in the absence and presence of metabolic activation [40]. Compounds ZVS-08 and **8** were cytotoxic to both bacterial strains (−/+ S9 metabolic activation) at concentrations ≥0.5 mg/plate. At non-cytotoxic concentrations (0.0158–0.158 mg/plate), no mutagenic response was observed in the absence (−S9) or presence (+S9) of metabolic activation (Tables S2 and S3).

## 4. Discussion

Inhibition of K_V_10.1 potassium channels with small molecules or antibodies reduces cancer cell growth in vitro and in vivo. In a retrospective study in brain metastases, treatment with agents that can inhibit K_V_10.1 improved the outcome of patients who had moderate expression of the channel [27]. This, together with the very high frequency of abnormal expression of the channel in human tumours, implies that K_V_10.1 inhibitors with appropriate potency and selectivity may be useful in the treatment of many tumour types. With this goal in mind, we set out to develop a new generation of K_V_10.1 inhibitors.

The discovery of K_V_10.1-selective potassium channel inhibitors is challenging due to the high similarity with the hERG, especially the ion-conducting pore. Most of the known K_V_10.1 inhibitors (Figure 1) also show hERG inhibition, which may lead to undesirable QT interval prolongations in patients [28]. Moreover, the exact location of the binding site for most of these inhibitors has not yet been experimentally determined. Structural information about the K_V_10.1 inhibitor complex is also not available, which limits the structure-based design of new selective K_V_10.1 inhibitors. Considering these limitations, we selected purpurealidine-based K_V_10.1 inhibitors that have been shown to bind to the VSD of the channel [8]. Given the greater structural dissimilarity between K_V_10.1 and hERG in the VSD than in the pore [20], our goal was to discover a new structural class of selective K_V_10.1 inhibitors by computer-assisted ligand-based virtual screening. The latter can overcome the limitation that the exact location of the binding site is unknown, as it only uses information about the ligand. However, in such a case, active and inactive ligands from the same structural class should be used in the modelling because they are expected to bind at the same binding site and in the same orientation.

The novel structural class of diarylamine-based K_V_10.1 inhibitors presented here was identified by ligand-based virtual screening using a pharmacophore model. Pharmacophore modelling is a powerful method that aligns active ligands based on their pharmacophore features and then creates a ligand-based pharmacophore model consisting of pharmacophore features common to all active ligands. Our initial model performed well in the validation experiment as it was able to find a majority of the active ligands and no inactive ligands from the compound library. However, it was too complex and did not return any hits when used in the virtual screening of a library of 556,000 compounds. After a series of simplifications, we arrived at a model with only six features (compared to 14 features in the original model) that performed excellently, finding all active compounds and no inactive ones (Figure 7D). By using this model in the virtual screen, we found 18 potential hit compounds, nine of which were purchased and tested for K_V_10.1 inhibition. Compound ZVS-08 proved to be a hit with an IC_50_ value of 3.70 µM. The hit compound ZVS-08 represents a new structural class of K_V_10.1 inhibitors with diarylamine structure.

With the structure–activity relationship studies we identified structural parts important for K_V_10.1 inhibition of this new structural type of K_V_10.1 inhibitors. The most potent inhibitors from this series contain a combination of disubstituted phenyl ring with both *ortho*-nitro and *para*-trifluoromethyl groups and basic moieties such as dimethylamino or morpholino group. Among all virtual hits, the compound ZVS-08 was the only one potent against the K_V_10.1. Amine groups and aromatic rings are also present in all ZVS virtual hits, but it might be possible that the substitution of the aromatic ring plays an important role in compound binding to the active site. Hit compound ZVS-08 has the ring substituted with two electron withdrawing groups that are not present in any other virtual hit. Based on results, these two groups are important for K_V_10.1 inhibition, as evident also in the optimisation of ZVS-08. Structural optimization resulted in a nanomolar K_V_10.1 inhibitor 1 (IC_50_ value of 740 nM). With structural optimization, we improved only the potency for K_V_10.1 but not for hERG, which remains the same as for the hit compound ZVS-08. New solutions are needed for father optimization of the selectivity of K_V_10.1 inhibitors against hERG channel.

The kinetics of inhibiting by ZVS-08 and its analogues resemble those of known com-pounds. The slow reduction in current amplitude along the stimulus is similar to inhibition by astemizole and is interpreted as inhibition of open channels. Nevertheless, this profile does not rule out an allosteric effect on gating, as mibefradil induces a kinetically similar effect. ZVS-08 induces a shift in the voltage dependence of K_V_10.1 currents in the hyperpolarizing direction reminiscent of that observed with mibefradil and with purpurealidine analogues, supporting the notion of an effect as a gating modifier rather than a blocker of open channels.

The exact binding site for the new compounds is still unknown. It is assumed that the parent structures bind to the VSD at a different site than mibefradil [8]. This also seems to be the case for the hit compound, as we observed no evidence of competition between ZVS-08 and mibefradil. We also tested competition with astemizol, due to the promiscuity of its binding site, but again we did not observe any competition. Therefore, we hypothesize that the new compounds bind to the VSD, probably from the inside. Mutagenesis experiments should help to identify the binding site [6]. The similarity between the behaviour of the ZVS-08 hit compound and astemizole would suggest that our hit shares the mechanism of inhibition and thus possibly the binding site in the channel pore and does not bind to the VSD like the purpurealidins from which our pharmacophore model was derived. We performed competition studies to test for a shared binding site. We determined the effect of different concentrations of ZVS-08 in the presence or absence of 500 nM astemizole (Figure 10). We observed no difference in affinity between the two conditions and conclude that ZVS-08 and astemizole do not share the same binding site.

The main interest of selective K_V_10.1 inhibitors would be their use as anticancer agents. ZVS-08 and compound **1** inhibited the growth of non-tumour and tumour cells without K_V_10.1 expression in conventional 2D culture at concentrations higher than 10 µM. At lower concentrations, however, ZVS-08 and compound **1** derivatives were able to inhibit efficiently the growth of MCF-7 breast cancer cells, while leaving non-transformed cells unaffected. MCF-7 cells show significant expression levels of K_V_10.1, but undetectable expression of hERG, indicating that the effect might be related to inhibition of K_V_10.1. Importantly, the compounds did not affect the growth of Panc1 cells, which abundantly express hERG, but not K_V_10.1. There was therefore a correlation between K_V_10.1 expression and sensitivity to the compounds. In tumour spheroids, all compounds were able to induce significant levels of apoptosis. In the case of the hit compound ZVS-08, the effect was remarkably fast and intense. Compound **8**, which was ineffective against tumour cells in monolayer cultures showed a slower onset in the induction of apoptosis but was able to induce cell death in longer times, which might indicate a more difficult access to the cells in the spheroid for this compound. These results do not exclude alternative mechanisms of action for the compounds, and further work will be required to determine to what extent Kv10.1 block is responsible for the effects observed.

## 5. Conclusions

To discover a novel structural class of K_V_10.1 inhibitors overexpressed in many different tumour types, we used a computational ligand-based approach. We identified the novel low micromolar K_V_10.1 inhibitor ZVS-08, which is based on a diarylamine scaffold. The inhibition profile of ZVS-08 with the combination of data from the competition experiments suggests that the binding site is not shared with two classical K_V_10.1 inhibitors. By synthesising the analogues, we were able to increase the potency of K_V_10.1 inhibition. Analogue compound 1 inhibited the K_V_10.1 channel with an IC_50_ value of 740 nM and showed a slight increase in selectivity on the hERG channel. Cancer cell proliferation was inhibited at a concentration of 5.48 μM in K_V_10.1 expressing cell line, while the cancerous and non-cancerous cell lines without K_V_10.1 expression were not inhibited. With the broad window between mutagenic activity and inhibition of K_V_10.1, the new compounds show promising potential for further development for cancer therapy.

## Figures and Tables

**Table 1:** List of screening compounds with pharmacophore fit scores and activity on K_V_10.1 at compound concentration of 50 μM.

Compound ID	Structure	Pharmacophore Fit Score	Effect on Kv10.1 (% Inhibition)
ZVS-01	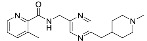	66.4900	−1.13 ± 4.50 (3)
ZVS-02	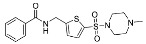	66.4745	5.03 ± 2.87 (3)
ZVS-03	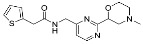	66.4421	−1.14 ± 3.47 (5)
ZVS-04	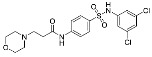	66.0596	−0.86 ± 0.75 (5)
ZVS-05	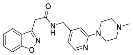	65.9545	8.84 ± 4.92 (6)
ZVS-06	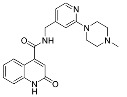	65.8501	−7.50 ± 4.65 (4)
ZVS-07	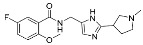	65.8019	4.68 ± 1.63 (5)
ZVS-08	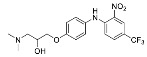	65.7908	80.27 ± 0.63 (3)
ZVS-09	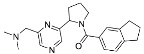	65.1035	0.15 ± 1.67 (4)

Compound ID: screening name of the compound; Pharmacophore fit score: scoring function calculated from the alignment of the pharmacophore features and the feature RMS deviation. Inhibition data are means ± standard error for the number of independent experiments indicated in parentheses.

**Table 2:** Potencies of compounds ZVS-08, **1** and **8** against K_V_10.1 and hERG.

Compound ID	IC_50_ Kv10.1 (μM)	IC_50_ hERG (μM)
ZVS-08	3.70 µM (95% CI: 2.08 µM to 6.47 µM)	0.194 µM (95% CI: 0.047 µM to 0.761 µM)
1	0.740 µM (95% CI: 490 nM to 1.17 µM)	0.207 µM (95% CI: 0.113 µM to 0.395 µM)
8	1.01 µM (95% CI: 796 nM to 1.30 µM	0.156 µM (95% CI: 0.070 µM to 0.373 µM)

**Table 3:** Structures and potencies of the designed and synthesised ZVS -08 analogues **1–12**.

Compound ID	Structure	% of Kv10.1 Inhibition at 10 μM
**1**	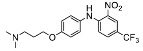	87.32 ± 4.60 (7)
**2**	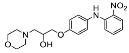	23.93 ± 26.80 (4)
**3**	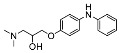	22.08 ± 33.52 (4)
**4**	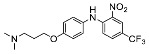	36.63 ± 19.89 (7)
**5**	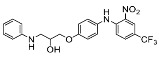	2.53 ± 11.73 (3)
**6**	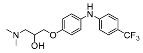	57.48 ± 28.38 (3)
**7**	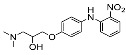	20.96 ± 30.27 (7)
**8**	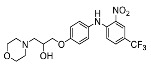	81.20 ± 4.97 (3)
**9**	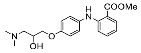	2.00 ± 7.45 (4)
**10**	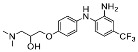	57.05 ± 21.71 (2)
**11**	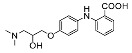	37.67 ± 7.13 (2)
**12**	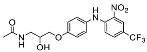	16.60 ± 38.36 (2)

Inhibition data are means ± standard error (or range) for the number of independent experiments indicated in parentheses.

## Data Availability

The data presented in this study are available on request from the corresponding authors.

## Supplementary Materials

The following are available online at https://www.mdpi.com/2072-6694/13/6/1244/s1. Table S1: Selectivity screening of ZVS-08 and 1 against KV and NaV channels. Table S2: Mutagenic activity of compound ZVS-08. Table S3: Mutagenic activity of compound 8, chemistry: analytical procedures description and NMR spectrums.
