# Supplementary material for: 3D Pharmacophore-Based Discovery of Novel KV10.1 Inhibitors with Antiproliferative Activity
Source: Cancers (Basel). 2021 Mar 12;13(6):1244. doi: 10.3390/cancers13061244 (PMC8002023; doi:10.3390/cancers13061244)
Supplement: Supplementary file 1 [file cancers-13-01244-s001.zip › cancers-1111130-suppplementary.pdf]

cancers

MDPI

Supplementary Materials: 3D Pharmacophore-Based Discovery of Novel Kv10.1 Inhibitors with Antiproliferative Activity

Žan Toplak [1], Louise Antonia Hendrickx [2], Špela Gubič [1], Štefan Možina [1], Bojana Žegura [3,4], Alja Štern [3,4], Matjaž Novak [3,4], Xiaoyi Shi [5], Steve Peigneur [2,] Jan Tytgat [2], Tihomir Tomašič [1], Luis A. Pardo [5,*], Lucija Peterlin Mašič [1,*]

[1] University of Ljubljana, Faculty of Pharmacy, Aškerčeva 7, 1000 Ljubljana; Slovenia; zan.toplak@ffa.uni-lj.si (Ž.T.); spela.gubic@ffa.uni-lj.si (Š.G.); stefan.mozina@ffa.uni-lj.si (Š.M.); tihomir.tomasic@ffa.uni-lj.si (T.T.); lucija.peterlin@ffa.uni-lj.si (L.P.M)

[2] University of Leuven, Toxicology and Pharmacology, Campus Gasthuisberg, Onderwijs en Navorsing 2, Herestraat 49, PO Box 922, 3000 Leuven, Belgium; louise.hendrickx@kuleuven.be (L.A.H.); steve.peigneur@kuleuven.be (S.P.); jan.tytgat@kuleuven.be (J.T.)

[3] National Institute of Biology, Department of Genetic Toxicology and Cancer Biology, Večna pot 111, 1000 Ljubljana, Slovenia; bojana.zegura@nib.si (B.Z); alja.stern@nib.si (A.S.); matjaz.novak@nib.si (M.N.)

[4] University of Ljubljana, Kongresni trg 12, 1000 Ljubljana

[5] AG Oncophysiology, Max-Planck Institute for Experimental Medicine, Hermann-Rein-Str. 3, 37075 Göttingen, Germany; shi@em.mpg.de (X.S.); pardo@em.mpg.de (L.A.P)

* Correspondence: pardo@em.mpg.de (L.A.P.); lucija.peterlin@ffa.uni-lj.si (L.P.M)

Supplementary tables

Table S1: Selectivity screening of ZVS-08 and **1** against voltage-gated potassium and sodium channels at a concentration of 10μM. Data were obtained with the two-electrode voltage clamp on *Xenopus laevis* oocytes.

Ion channel	Compound tested at 10μM	Average block (%)	SD	SEM	N
Kv1.1	ZVS-08	-2.30	9.01	4.51	4
	1	5.36	4.42	2.55	3
Kv1.3	ZVS-08	0.15	3.76	2.17	3
	1	1.65	1.90	1.10	3
Kv1.4	ZVS-08	-0.68	5.22	3.01	3
	1	0.18	4.06	2.34	3
Kv2.1	ZVS-08	6.66	2.79	1.61	3
	1	6.54	2.74	1.58	3
Kv4.2	ZVS-08	5.76	5.13	2.96	3
	1	19.51	23.72	8.97	7
Nav1.4	ZVS-08	4.27	6.89	3.98	3
	1	-2.97	4.23	2.11	4
Nav1.5	ZVS-08	9.31	1.95	0.98	4

1	3.18	2.56	1.28	4

SD- standard deviation; SEM - standard error of the mean; N – number of independent experiments

Table S2: Mutagenic activity of compound ZVS-08 determined by the *Salmonella*/microsomal reverse mutation assay with *S. typhimurium* strains TA98 and TA100 in the presence and absence of S9 metabolic activation. Revertants are presented as means of triplicate plates ± standard deviation and induction factor.

Average number of revertant colonies (mean ± SD) and induction factor (IF)

mg/plate	TA98		TA100	
	-S9	+S9	-S9	+S9
NC	27.33 ± 3.06	44.00 ± 3.60	94.33 ± 3.06	115.67 ± 20.65
	(1.08)	(1.21)	(1.06)	(1.15)
PC	341.00 ± 11.79	220.00 ± 18.25	464.00 ± 42.58	635 ± 43.97
	(13.46)	(6.06)	(42.58)	(6.31)
0	25.33 ± 3.06	36.33 ± 6.66	88.67 ± 7.77	100.67 ± 16.07
	(1)	(1)	(1)	(1)
0.0158	21.67 ± 4.51	31.00 ± 3.61	114.00 ±2.00	102.67 ± 15.01
	(0.86)	(0.85)	(1.29)	(1.02)
0.05	22.67 ± 8.08	45.33 ± 8.62	99.67 ± 16.80	81.67 ± 14.57
	(0.89)	(1.25)	(1.12)	(0.81)
0.158	17.67 ± 3.79	34.67 ± 14.05	81.00 ± 5.29	71.00 ± 7.21
	(0.70) [a]	(0.95) [a]	(0.91) [a]	(0.71) [a]
0.5	0 [c]	0 [b]	0 [c]	0 [b]
1.58	0 [d]	0 [d]	0 [d]	0 [d]
5	0 [d]	0 [d]	0 [d]	0 [d]

IF- induction factor for each concentration is shown in brackets; IF ≥ 2 is considered a positive response; the bacterial background lawn is considered as normal if not stated otherwise; [a] slightly reduced bacterial background; [b] moderately reduced bacterial background; [c] extremely reduced bacterial background; [d] bacterial background is absent.

Table S3: Mutagenic activity of compound **8** determined by the *Salmonella*/microsomal reverse mutation assay with *S. typhimurium* strains TA98 and TA100 in the presence and absence of S9 metabolic

activation. Revertants are presented as means of triplicate plates ± standard deviation and induction factor.

Average number of revertant colonies (mean ± SD) and induction factor (IF)

mg/plate	TA98		TA100	
	-S9	+S9	-S9	+S9
NC	27.33 ± 3.06 (1.08)	44.00 ± 3.60 (1.21)	104.33 ± 7.77 (1.05)	97.67 ± 7.64 (0.91)
PC	341.00 ± 11.79 (13.46)	220.00 ± 18.25 (6.06)	473.67 ± 38.68 (4.77)	641.00 ± 57.00 (5.97)
0	25.33 ± 3.0 (1)	36.33 ± 6.66 (1)	99.33 ± 2.52 (1)	107.33 ± 21.57 (1)
0.0158	21.67 ± 3.21 (0.86)	25.00 ± 5.57 (0.69)	90.33 ± 5.51 (0.91)	94.67 ± 13.50 (0.88)
0.05	20.33 ± 2.52 (0.80)	43.33 ± 9.29 (1.19)	100.00 ± 8.89 (1.01)	99.33 ± 6.66 (0.93)
0.158	17 ± 3.46 (0.67) [a]	37.33 ± 3.21 (1.03) [a]	82.67 ± 7.23 (0.83) [a]	84.33 ± 8.50 (0.79) [a]
0.5	0 [b]	0 [b]	0 [b]	77.67 ± 8.74 (0.72) [b]
1.58	0 [c]	0 [c]	0 [c]	44.33 ± 11.59 (0.41) [c]
5	0 [c]	0 [c]	0 [c]	11.33 ± 3.06 (0.11) [c]

IF- induction factor for each concentration is shown in brackets; IF ≥ 2 is considered a positive response; the bacterial background lawn is considered as normal if not stated otherwise; [a] slightly reduced bacterial background; [b] moderately reduced bacterial background; [c] extremely reduced bacterial background.

Chemistry

Reagents and solvents were obtained from Acros Organics (Geel, Belgium), Sigma-Aldrich (St. Louis, MO, USA) and Apollo Scientific (Stockport, UK). Merck 60 F254 plates (0.25 mm) were used for the TLC analysis visualized with UV light and spray reagents. Flash column chromatography was performed on silica gel 60 (particle size 240–400 mesh). Melting points were determined on a hot-stage microscope from Reichert. ^{1}H and ^{13}C NMR spectra were recorded at 400 and 100 MHz on a Bruker AVANCE III 400 spectrometer (Bruker Corporation, MA, USA) with the DMSO-d_6 or $CDCl_3$ solutions. For HPLC analyses an Thermo Scientific Dionex Ultimate 3000 (Thermo Fisher Scientific, Waltham, MA, USA) and Acquity UPLC C18 column (1,7 μm, 2,1 mm × 50 mm). Mobile phase consisted of acetonitrile (as solvent A) and 0.1% fumaric acid and ultrapure water (as solvent B). The gradient for solvent A was 10–90% in 8 min and 90% to 11 min with flow rate 0.4 mL/min and injection volume: 2 μL. Mass spectra were obtained using ADVION expression CMSL mass spectrometer (Advion Inc., Ithaca, USA).

Synthetic procedures and analytical data

***N*-(4-(3-(Dimethylamino)propoxy)phenyl)-2-nitro-4-(trifluoromethyl)aniline (1).** To solution of **15** (100 mg, 0.34 mmol) in *N*,*N*-dimethylformamide (DMF) (3 mL) Cs_2CO_3 (163 mg, 0.50 mmol) and 3-dimethylamino-1-propyl chloride hydrochloride (83 mg, 0.50 mmol) were added, flask was flushed with argon and stirred at 100 °C for 3 h. The reaction mixture was filtered, filtrate was poured over ice

(50 mL) and extracted with ethyl acetate (3 × 50 mL) that was washed with brine (2 × 25 mL) and dried over Na_2SO_4. Solvent was removed under reduced pressure and residue was purified using Biotage Isolera One System reversed-phase chromatography (Biotage SNAP Cartridge KP-C18-HS 12 g column), MF: gradient 0.1 % trifluoroacetic acid in H_2O/acetonitril). Yield: 31.1 %; orange crystals (40 mg); ^{1}H NMR (400 MHz, $CDCl_3$) δ 9.60 (s, 1H), 8.48 (dd, J_1 = 2.1 Hz, J_2 = 1.1 Hz, 1H), 7.48 (dd, J_1 = 9.1 Hz, J_2 = 2.2 Hz, 1H), 7.17 (d, J = 8.8 Hz, 2H), 7.03 (d, J = 9.1 Hz, 1H), 7.01 – 6.94 (m, 2H), 4.05 (t, J = 6.4 Hz, 2H), 2.48 (t, J = 7.2 Hz, 2H), 2.05 – 1.93 (m, 2H); ^{13}C NMR (101 MHz, $CDCl_3$) δ 158.19, 146.52, 131.85, 131.82, 131.79, 131.76, 131.34, 129.95, 127.63, 127.53, 126.55, 124.94, 124.89, 124.85, 124.81, 124.76, 122.25, 119.56, 119.32, 118.98, 118.64, 118.30, 116.65, 115.89, 66.64, 56.40, 45.62, 27.57 ppm; HRMS (ESI$^+$) for $C_{18}H_{21}F_3N_3O_3$ ($[M+H]^+$) calculated 384.1524, found 384.1530; HPLC retention time: 4.047 min (97.3 % at 254 nm).

General procedure A. Synthesis of compounds 2 and 8 (with 2 as an example). To a solution of **19** (200 mg, 0.699 mmol) in methanol (5 mL), cooled to 0 °C on an ice bath,morpholine (0.074 mL, 0.838 mmol) was added. The reaction mixture was stirred for 24 h at r.t. and then additional amount of morpholine (0.074 mL, 0.838 mmol) was added. After 24 h the solvent was removed under reduced pressure and product was purified with flash column chromatography using dichloromethane/methanol = 20:1 as eluent.

1-Morpholino-3-(4-((2-nitrophenyl)amino)phenoxy)propan-2-ol (2). Yield: 88.5 %; red crystals (231 mg); mp: 95.6-97.5 °C; ^{1}H NMR (400 MHz, $CDCl_3$) δ 9.40 (s, 1H, NH), 8.19 (dd, J_1 = 8.6 Hz, J_2 = 1.6 Hz, 1H, Ar-H), 7.32 (dddd, J_1 = 8.6 Hz, J_2 = 6.9 Hz, J_3 = 1.7 Hz, J_4 = 0.6 Hz, 1H, Ar-H), 7.23 – 7.14 (m, 2H, 2 × Ar-H), 7.05 – 6.94 (m, 3H, 3 × Ar-H), 6.72 (ddd, J_1 = 8.6 Hz, J_2 = 6.9 Hz, J_3 = 1.6 Hz, 1H, Ar-H), 4.19 – 4.07 (m, 1H, CH), 4.02 (dd, J_1 = 4.9 Hz, J_2 = 1.4 Hz, 2H, CH_2), 3.75 (dt, J_1 = 5.7 Hz, J_2 = 3.4 Hz, 4H, 2 × CH_2), 2.70 (dt, J_1 = 9.8 Hz, J_2 = 4.8 Hz, 2H, CH_2), 2.65 – 2.54 (m, 2H, CH_2), 2.49 (dt, J_1 = 10.4 Hz, J_2 = 4.1 Hz, 2H, CH_2) ppm; ^{13}C NMR (101 MHz, $CDCl_3$) δ 157.11, 144.46, 135.86, 132.61, 131.67, 127.14, 126.73, 116.97, 115.85, 115.75, 70.60, 67.12, 65.45, 61.08, 53.86 ppm; HRMS (ESI$^+$) for $C_{19}H_{24}N_3O_5$ ($[M+H]^+$) calculated 374.1711, found 374.1705; HPLC retention time: 4.090 min (99.5 % at 254 nm).

General procedure B. Synthesis of compounds ZVS-08, 3, 6, 7 and 9 (with 3 as an example). To a solution of **24** (170 mg, 0.706 mmol) in methanol (10 mL), Me_2NH × HCl (172 mg, 2.110 mmol) was added and the mixture was stirred at 70 °C for 24 h. The solvent was removed under reduced pressure and crude product was purified with Biotage Isolera One System reversed-phase chromatography (Biotage SNAP Cartridge KP-C18-HS 12 g column), MF: gradient 0.1 % trifluoroacetic acid in H_2O/acetonitril). Fractions containing **3** were combined, acetonitrile was removed under reduced pressure and pH of the remaining water phase was adjusted to 8. The product was extracted with ethyl acetate (2 × 25 mL). Organic phase was washed with brine (2 × 25 mL), dried over Na_2SO_4, filtered and the solvent removed under reduced pressure to obtain **3** (25 mg).

1-(Dimethylamino)-3-(4-(phenylamino)phenoxy)propan-2-ol (3).

Yield: 12.5%; yellowish crystals (25 mg); mp: 88.0-90.1 °C; ^{1}H NMR (400 MHz, $CDCl_3$) δ 7.24 – 7.18 (m, 2H, 2 × Ar-H), 7.09 – 7.03 (m, 2H, 2 × Ar-H), 6.94 – 6.80 (m, 5H, 5 × Ar-H), 5.49 (s, 1H, NH), 4.10 – 4.02 (m, 1H, CH), 3.98 – 3.93 (m, 2H, CH_2), 2.56 (dd, J_1 = 12.2 Hz, J_2 = 9.9 Hz, 1H, CH_2), 2.40 (dd, J_1 = 12.2 Hz, J_2 = 3.7 Hz, 1H, CH_2), 2.34 (s, 6H, 2 × CH_3) ppm; ^{13}C NMR (101 MHz, $CDCl_3$) δ 154.41, 145.09, 136.16, 129.37, 121.95, 119.69, 115.84, 115.53, 70.96, 66.42, 62.00, 45.70 ppm; HRMS (ESI$^+$) for $C_{17}H_{23}N_2O_2$ ($[M+H]^+$) calculated 287.1754, found 287.1751; HPLC retention time: 3.117 min (99.7 % at 254 nm).

***N*-(4-(3-(Dimethylamino)propoxy)phenyl)-2-nitroaniline (4).** To solution of **18** (100 mg, 0.43 mmol) in DMF (3 mL), Cs_2CO_3 (212 mg, 0.43 mmol) and 3-dimethylamino-1-propyl chloride hydrochloride (107 mg, 0.65 mmol) were added, flask was flushed with argon and stirred at 100 °C for 3 h. The mixture was filtered, filtrate was poured over ice water (50 mL) and extracted with ethyl acetate (3 × 50 mL).

Combined organic phase was washed with brine (2 × 25 mL) and dried over Na_2SO_4. Solvent was removed under reduced pressure and the residue purified using Biotage Isolera One System reversed-phase chromatography (Biotage SNAP Cartridge KP-C18-HS 12 g column), MF: gradient 0.1 % trifluoroacetic acid in H_2O/acetonitril).

Yield: 10.9 %; orange crystals (15 mg); ^{1}H NMR (400 MHz, $CDCl_3$) δ 9.40 (s, 1H), 8.18 (dd, J_1 = 8.7 Hz, J_2 = 1.6 Hz, 1H), 7.31 (ddd, J_1 = 8.4 Hz, J_2 = 6.8 Hz, J_3 = 1.6 Hz, 1H), 7.21 – 7.14 (m, 2H), 7.00 (d, J = 1.3 Hz, 1H), 6.94 (d, J = 8.9 Hz, 2H), 6.70 (ddd, J_1 = 8.4 Hz, J_2 = 6.9 Hz, J_3 = 1.3 Hz, 1H), 4.04 (t, J = 6.3 Hz, 2H), 2.60 (t, J = 7.4 Hz, 2H), 2.36 (s, 6H), 2.03 (dq, J_1 = 8.0 Hz, J_2 = 6.4 Hz, 2H); ^{13}C NMR (101 MHz, $CDCl_3$) δ 157.23, 144.48, 135.75, 132.43, 131.18, 127.08, 126.59, 116.78, 115.77, 115.52, 66.26, 56.24, 45.15, 27.08 ppm; HRMS (ESI$^+$) for $C_{17}H_{22}N_3O_3$ ([M+H]$^+$) calculated 316.1654, found 316.1656; HPLC retention time: 3.277 min (97.5 % at 254 nm).

1-(4-((2-Nitro-4-(trifluoromethyl)phenyl)amino)phenoxy)-3-(phenylamino)propan-2-ol (5). To a solution of **16** (100 mg, 0.282 mmol) in methanol (5 mL), cooled to 0 °C on an ice bath, aniline (0.031 mL, 0.338 mmol) was added and the reaction mixture stirred for 16 h at r.t. Then additional amount of aniline (0.031 mL, 0.338 mmol) was added. After 24 h the solvent was removed under reduced pressure and crude product was purified with flash column chromatography using ethyl acetate/hexane = 1:2 as eluent.

Yield: 63.3 %; orange solid (85 mg); mp: 97.5-99.8 °C; ^{1}H NMR (400 MHz, $CDCl_3$) δ 9.62 (s, 1H, NH), 8.51 (dd, J_1 = 2.1 Hz, J_2 = 1.1 Hz, 1H, Ar-H), 7.50 (dd, J_1 = 9.1 Hz, J_2 = 2.2 Hz, 1H, Ar-H), 7.26 – 7.17 (m, 4H, 4 × Ar-H), 7.08 – 6.97 (m, 3H, 3 × Ar-H), 6.77 (tt, J_1 = 7.3 Hz, J_2 = 1.0 Hz, 1H, Ar-H), 6.74 – 6.65 (m, 2H, 2 × Ar-H), 4.36 – 4.25 (m, 1H, CH), 4.17 – 4.05 (m, 2H, CH_2), 3.47 (dd, J_1 = 13.1 Hz, J_2 = 4.4 Hz, 1H, CH_2), 3.34 (dd, J_1 = 13.1 Hz, J_2 = 7.1 Hz, 1H, CH_2), 2.51 (d, J = 4.5 Hz, 1H, OH) ppm; ^{13}C NMR (101 MHz, $CDCl_3$) δ 157.55, 148.09, 146.33, 131.91, 131.88, 131.84, 131.81, 131.52, 130.76, 129.51, 129.48, 127.62, 124.93, 124.89, 124.85, 124.81, 122.23, 119.55, 119.21, 118.87, 118.53, 118.35, 118.30, 116.57, 116.53, 116.02, 113.47, 113.44, 70.59, 68.90, 46.73 ppm; HRMS (ESI$^+$) for $C_{22}H_{21}N_3O_4F_3$ ([M+H]$^+$) calculated 448.1479, found 448.1474; HPLC retention time: 6.720 min (95.6 % at 254 nm).

1-(Dimethylamino)-3-(4-((4-(trifluoromethyl)phenyl)amino)phenoxy)propan-2-ol (6). Synthesised according to General procedure B with 3 equivalents of Me_2NH × HCl.

Yield: 29.6 %; light brown crystals (38 mg); mp: 76.3-79.1 °C; ^{1}H NMR (400 MHz, $CDCl_3$) δ 7.41 (d, J = 8.4 Hz, 2H, 2 × Ar-H), 7.14 – 7.07 (m, 2H, 2 × Ar-H), 6.95 – 6.89 (m, 2H, 2 × Ar-H), 6.86 (d, J = 8.4 Hz, 2H, 2 × Ar-H), 5.72 (s, 1H, NH), 4.13 – 4.02 (m, 1H, CH), 4.01 – 3.94 (m, 1H, CH_2), 2.57 (dd, J_1 = 12.2 Hz, J_2 = 9.9 Hz, 1H, CH_2), 2.40 (dd, J_1 = 12.2 Hz, J_2 = 3.8 Hz, 1H, CH_2), 2.39 (d, J = 3.8 Hz, 1H, CH_2), 2.34 (s, 6H, 2 × CH_3) ppm; ^{13}C NMR (101 MHz, $CDCl_3$) δ 155.66, 148.57, 134.11, 128.89, 126.81, 126.77, 126.73, 126.70, 126.21, 124.12, 123.52, 121.02, 120.83, 120.70, 120.37, 120.05, 115.62, 113.91, 70.85, 66.33, 61.90, 45.69 ppm; HRMS (ESI$^+$) for $C_{18}H_{22}N_2O_2F_3$ ([M+H]$^+$) calculated 355.1628, found 355.1624; HPLC retention time: 4.183 min (99.0 % at 254 nm).

1-(Dimethylamino)-3-(4-((2-nitrophenyl)amino)phenoxy)propan-2-ol (7). Synthesised according to General procedure B with 4 equivalents of Me_2NH × HCl.

Yield: 30.4%; orange crystals (70 mg); mp: 76.1-77.3 °C; ^{1}H NMR (400 MHz, $CDCl_3$) δ 9.41 (s, 1H, NH), 8.19 (dd, J_1 = 8.6 Hz, J_2 = 1.5 Hz, 1H, Ar-H), 7.32 (ddd, J_1 = 6.9 Hz, J_2 = 4.3 Hz, J_3 = 1.5 Hz, 1H, Ar-H), 7.22 – 7.16 (m, 2H, Ar-H), 7.03 – 6.94 (m, 3H, 3 × Ar-H), 6.71 (ddd, J_1 = 8.6 Hz, J_2 = 6.9 Hz, J_3 = 1.5 Hz, 1H , Ar-H), 4.15 – 4.04 (m, 1H, CH), 4.02 – 3.97 (m, 1H, CH_2), 2.59 (dd, J_1 = 12.2 Hz, J_2 = 10.0 Hz, 1H, CH_2), 2.42 (dd, J_1 = 12.2 Hz, J_2 = 3.7 Hz, 1H, CH_2), 2.35 (s, 6H, 2 × CH_3) ppm; ^{13}C NMR (101 MHz, $CDCl_3$) δ 157.20, 144.51, 135.87, 132.59, 131.58, 127.14, 126.72, 116.94, 115.87, 115.76, 70.80, 66.24, 61.86, 45.69 ppm; HRMS

(ESI+) for $C_{17}H_{22}N_3O_4$ ([M+H]+) calculated 332.1605, found 332.1599; HPLC retention time: 4.083 min (98.4 % at 254 nm).

1-Morpholino-3-(4-((2-nitro-4-(trifluoromethyl)phenyl)amino)phenoxy)propan-2-ol (8). Synthesised according to General procedure A with second addition 0.6 equivalents of morpholine and reaction was stopped after 12 h. Additional purification with flash column chromatography acetonitrile/acetone = 1:1 was needed to obtain pure product.

Yield: 31.3 %; orange crystals (39 mg); mp: 90.0-92.3 °C; ^{1}H NMR (400 MHz, $CDCl_3$) δ 9.61 (s, 1H, NH), 8.50 (d, *J* = 1.1 Hz, 1H, Ar-H), 7.49 (dd, J_1 = 9.1 Hz, J_2 = 2.3 Hz, 1H, Ar-H), 7.23 – 7.16 (m, 2H, 2 × Ar-H), 7.07 – 6.97 (m, 3H, 3 × Ar-H), 4.19 – 4.10 (m, 1H, CH), 4.03 (dd, J_1 = 4.8 Hz, J_2 = 2.4 Hz, 2H, CH_2), 3.75 (dt, J_1 = 5.7 Hz, J_2 = 3.5 Hz, 4H, 2 × CH_2), 3.49 (s, 1H, OH), 2.70 (dt, J_1 = 9.6 Hz, J_2 = 4.9 Hz, 2H, CH_2), 2.64 – 2.53 (m, 2H, CH_2), 2.49 (dt, J_1 = 10.2 Hz, J_2 = 4.6 Hz, 2H, CH_2) ppm; ^{13}C NMR (101 MHz, $CDCl_3$) δ 157.86, 146.39, 131.84, 131.81, 131.47, 130.48, 127.62, 127.54, 124.93, 124.88, 124.83, 122.24, 119.55, 119.47, 119.13, 118.79, 118.45, 116.60, 116.02, 70.68, 67.13, 65.45, 61.07, 53.88 ppm; HRMS (ESI+) for $C_{20}H_{23}N_3O_5F_3$ ([M+H]+) calculated 442.1584 found, 442.1579; HPLC retention time: 4.927 min (99.0 % at 254 nm).

Methyl 2-((4-(3-(dimethylamino)-2-hydroxypropoxy)phenyl)amino)benzoate (9). Synthesised according to General procedure B with 2 equivalents of Me_2NH × HCl and **28**.

Yield: 24.0 %; light brown crystals (40 mg); ^{1}H NMR (400 MHz, $CDCl_3$) δ 9.29 (s, 1H), 7.94 (dd, J_1 = 8.1 Hz, J_2 = 1.6 Hz, 1H), 7.26 (d, *J* = 1.5 Hz, 1H), 7.20 – 7.12 (m, 2H), 6.98 (dd, J_1 = 8.6 Hz, J_2 = 1.1 Hz, 1H), 6.91 – 6.85 (m, 2H), 6.68 (ddd, J_1 = 8.1 Hz, J_2 = 7.1 Hz, J_3 = 1.1 Hz, 1H), 4.49 (tt, J_1 = 8.5 Hz, J_2 = 4.2 Hz, 1H), 4.15 (dd, J_1 = 9.4 Hz, J_2 = 4.5 Hz, 1H), 3.94 (dd, J_1 = 9.4 Hz, J_2 = 7.5 Hz, 1H), 3.89 (s, 3H), 3.37 – 3.26 (m, 2H), 2.97 (d, *J* = 15.5 Hz, 6H) ppm; HRMS (ESI+) for $C_{19}H_{25}N_3O_4$ ([M+H]+) calculated 345.1805, found 345.1809; HPLC retention time: 3.970 min (99.1 % at 254 nm).

1-(4-((2-Amino-4-(trifluoromethyl)phenyl)amino)phenoxy)-3-(dimethylamino)propan-2-ol (10). To an argon flushed solution of ZVS-08 (47 mg, 0.119 mmol) in methanol (2 mL) 10 mg of Pd/C was added and the reaction mixture was stirred under hydrogen atmosphere for 2 h. The catalyst was filtered off and the solvent removed under reduced pressure. The residue was dissolved in diethyl ether (2 mL), impurities were filtered off and the solvent was removed under reduced pressure.

Yield: 46.0 %; off-white crystals (20 mg); mp: 85.8-87.5 °C; R^{1}H NMR (400 MHz, $CDCl_3$) δ 7.04 – 6.94 (m, 3H, 3 × Ar-H), 6.89 (s, 4H, 4 × Ar-H), 5.22 (s, 1H, NH), 4.06 (ddt, J_1 = 9.8 Hz, J_2 = 5.6 Hz, J_3 = 4.2 Hz, 1H, CH), 3.98 – 3.92 (m, 2H, CH_2), 3.69 (s, 2H, NH_2), 2.56 (dd, J_1 = 12.2 Hz, J_2 = 9.9 Hz, 1H, CH_2), 2.39 (dd, J_1 = 12.2 Hz, J_2 = 3.7 Hz, H), 2.33 (s, 6H, 2 × CH_3) ppm; ^{13}C NMR (101 MHz, $CDCl_3$) δ 154.40, 137.59, 136.24, 135.95, 128.68, 125.98, 124.58, 124.26, 123.94, 123.62, 123.29, 120.87, 120.59, 117.75, 117.06, 117.02, 116.98, 116.94, 115.76, 113.36, 113.32, 113.28, 113.25, 71.03, 66.41, 61.95, 45.71 ppm; HRMS (ESI+) for $C_{18}H_{22}N_3O_2F_3$ ([M+H]+) calculated 370.1737, found 370.1732; HPLC retention time: 3.333 min (91.0 % at 254 nm).

2-((4-(3-(Dimethylamino)-2-hydroxypropoxy)phenyl)amino)benzoic acid (11). Compound **9** (30 mg, 0.087 mmol) was dissolved in methanol (2 mL) and 1 M NaOH (0.087 mL, 0.087 mmol) was added. The reaction mixture was stirred at r.t. for 12 h. The solvent was removed under reduced pressure. The residue was dissolved in concentrated citric acid (10 mL) and the product was extracted with ethyl acetate (3 × 10 mL). Organic solvent was removed under reduced pressure to give **11**.

Yield: 32.5 %; light brown crystals (13 mg); ^{1}H NMR (400 MHz, $CDCl_3$) δ 8.00 (dd, J_1 = 8.0 Hz, J_2 = 1.7 Hz, 1H), 7.32 – 7.29 (m, 1H), 7.17 – 7.10 (m, 2H), 6.98 (d, *J* = 8.2 Hz, 1H), 6.85 (d, *J* = 8.9 Hz, 2H), 6.73 – 6.66 (m, 1H), 4.51 (dt, J_1 = 8.2 Hz, J_2 = 4.1 Hz, 1H), 4.15 (dd, J_1 = 9.4 Hz, J_2 = 4.5 Hz, 1H), 3.99 – 3.91 (m, 1H),

3.35 – 3.28 (m, 2H), 2.97 (s, 7H), 2.63 (s, 2H) ppm; HRMS (ESI$^+$) for $C_{18}H_{23}N_2O_4$ ([M+H]$^+$) calculated 331.1649, found 331.16523; HPLC retention time: 3.153 min (99.4 % at 254 nm).

***N*-(2-Hydroxy-3-(4-((2-nitro-4-(trifluoromethyl)phenyl)amino)phenoxy)propyl)acetamide (12).** To argon flushed solution of **16** (200 mg, 0.565 mmol) in acetonitrile (3 mL), BF_3OEt_2 (80 mg, 0.072 mL, 0.656 mmol) was added and the reaction mixture stirred for 16 h at 80 °C. Reaction was stopped by adding saturated $NaHCO_3$ solution (5 mL), stirred for 5 min at r.t., then extracted with EtOAc (10 mL) and washed with brine (5 mL). Organic phase was dried over Na_2SO_4, filtered and then solvent removed under reduced pressure. The residue was purified with flash column chromatography using dichloromethane/methanol = 20:1, and once more using ethyl acetate/hexane = 2:1 as eluents.

Yield: 7.2 %; dark orange crystals (17 mg); mp: 69.7-71.8 °C; ^{1}H NMR (400 MHz, $CDCl_3$) δ 9.61 (s, 1H, NH), 8.50 (d, *J* = 1.1 Hz, 1H, Ar-H), 7.50 (dd, J_1 = 9.1 Hz, J_2 = 2.2 Hz, 1H, Ar-H), 7.23 – 7.17 (m, 2H, 2 × Ar-H), 7.07 – 6.97 (m, 3H, 3 × Ar-H), 4.90 (ddt, J_1 = 9.9 Hz, J_2 = 7.2 Hz, J_3 = 4.9 Hz, CH), 4.07 (d, *J* = 5.0 Hz, 2H, CH_2), 4.01 (ddd, J_1 = 14.2 Hz, J_2 = 10.0 Hz, J_3 = 1.6 Hz, 1H, CH_2), 3.74 (ddd, J_1 = 14.3 Hz, J_2 = 7.2 Hz, J_3 = 1.5 Hz, 1H, CH_2), 2.02 (t, *J* = 1.4 Hz, 3H, CH_3) ppm; HRMS (ESI$^-$) for $C_{18}H_{21}N_3O_5F_3$ ([M-H]$^+$) calculated 370.1737, found 370.1732; HPLC retention time: 4.523 min (87.3 % at 254 nm).

1-Chloro-2-nitro-4-(trifluoromethyl)benzene (14). To 4-chlorobenzotrifluoride (**13**, 1.49 mL, 11.08 mmol) cooled on an ice bath, mixture of HNO_3 (1.93 mL, 27.96 mmol) and H_2SO_4 (1.2 mL, 22.15 mmol) was added dropwise. The reaction mixture was stirred at 80 °C for one week, then poured over crushed ice (5 mL). The organic phase was then separated and used in the next step without further purification.

Yield: 64.4 %; yellowish oil (1.70 g); ^{1}H NMR (400 MHz, DMSO-d_6) δ 8.49 (d, *J* = 2.1 Hz, H, Ar-H), 8.08 (dd, J_1 = 8.5 Hz, J_2 = 2.1 Hz, Ar-H), 8.01 (d, *J* = 8.5 Hz, H, Ar-H) ppm.

4-((2-Nitro-4-(trifluoromethyl)phenyl)amino)phenol (15). Crude product **14** (340 mg, 1.51 mmol) was dissolved in isopropanol (6 mL), followed by addition of 4-aminophenol (329 mg, 3.01 mmol). The reaction mixture was stirred under reflux for 12 h. The solvent was removed under reduced pressure and crude product purified with flash column chromatography using ethyl acetate/hexane = 1:2 as eluent to obtain **15** (1.18 g).

Yield: 85.2 %; red crystals (383 mg); mp: 119.8-120.0 °C; ^{1}H NMR (400 MHz, $CDCl_3$) δ 9.59 (s, 1H, NH) 8.50 (d, *J* = 1.1 Hz, 1H, Ar-H), 7.50 (dd, J_1 = 9.1 Hz, J_2 = 2.2 Hz, 1H Ar-H), 7.18 – 7.12 (m, 2H, 2 × Ar-H), 7.04 (d, *J* = 9.1 Hz, 1H, Ar-H), 6.95 – 6.90 (m, 2H, 2 × Ar-H), 4.90 (s, 1H, OH) ppm; ^{13}C NMR (101 MHz, $CDCl_3$) δ 155.11, 146.57, 131.91, 131.88, 131.84, 131.81, 131.37, 130.08, 127.84, 127.64, 124.94, 124.91, 124.87, 124.83, 124.78, 122.25, 119.56, 119.41, 119.07, 118.72, 118.39, 116.92, 116.65, 31.13 ppm; MS (ESI-) m/z = 296.9 ([M-H]$^-$).

General procedure C. Synthesis of compounds 16, 19, 22, 24 and 28 (with 19 as an example). To an argon flushed solution of **17** (500 mg, 2.17 mmol) in MeCN (40 mL), cooled to 0 °C on an ice bath, K_2CO_3 (973 mg, 7.04 mmol) and (±)-epichlorohydrin (1.41 g, 15.20 mmol, 1.19 mL) were added. Reaction mixture was stirred at 80 °C for 16 h. K_2CO_3 was filtered off, the solvent was removed under reduced pressure and the remaining residue was purified with flash column chromatography using dichloromethane/methanol = 20:1 as eluent to obtain **19** (428 mg).

2-Nitro-*N*-(4-(oxiran-2-ylmethoxy)phenyl)aniline (19).

Yield: 68.8 %; orange crystals (428 mg) mp: 64.1-64.6 °C; ^{1}H NMR (400 MHz, $CDCl_3$) δ 9.40 (s, 1H, NH), 8.19 (dd, J_1 = 8.6 Hz, J_2 = 1.6 Hz, 1H, Ar-H), 7.36 – 7.30 (m, 1H, Ar-H), 7.23 – 7.16 (m, 2H, 2 × Ar-H), 7.04 – 6.95 (m, 3H, 3 × Ar-H), 6.72 (ddd, J_1 = 8.6 Hz, J_2 = 6.9 Hz, J_3 = 1.6 Hz, 1H, Ar-H), 4.28 (dd, J_1 = 11.1 Hz,

J_2 = 2.8 Hz, 1H, CH_2), 3.97 (dd, J_1 = 11.1 Hz, J_2 = 5.7 Hz, 1H, CH_2), 3.39 (ddt, J_1 = 5.7 Hz, J_2 = 4.1 Hz, J_3 = 2.8 Hz, 1H, CH), 2.94 (dd, J_1 = 4.9 Hz, J_2 = 4.1 Hz, 1H, CH_2), 2.79 (dd, J_1 = 4.9 Hz, J_2 = 2.8 Hz, 1H, CH_2) ppm; ^{13}C NMR (101 MHz, $CDCl_3$) δ 156.91, 144.44, 135.88, 132.73, 131.94, 127.17, 126.77, 117.04, 115.92, 115.89, 69.26, 50.27, 44.81 ppm; MS (ESI+) m/z = 309.3 ([M+Na]$^+$).

2-Nitro-*N*-(4-(oxiran-2-ylmethoxy)phenyl)-4-(trifluoromethyl)aniline (16). Synthesised according to General procedure C with 5 equivalents of (±)-epichlorohydrin and purified with flash column chromatography using dichloromethane/methanol = 200:1 as eluent.

Yield: 79.8 %; orange solid (180 mg); mp: 82.7-83.8 °C ^{1}H NMR (400 MHz, $CDCl_3$) δ 9.61 (s, 1H, NH), 8.53 – 8.48 (m, 1H, Ar-H), 7.50 (dd, J_1 = 9.1 Hz, 2.2 Hz, 1H, Ar-H), 7.23 – 7.17 (m, 2H, 2 × Ar-H), 7.08 – 6.98 (m, 3H, 3 × Ar-H), 4.30 (dd, J_1 = 11.1 Hz, J_2 = 2.9 Hz, 1H, CH_2), 3.98 (dd, J_1 = 11.1 Hz, J_2 = 5.8 Hz, 1H, CH_2) , 3.39 (ddt, J_1 = 5.8 Hz, J_2 = 4.1 Hz, J_3 = 2.9 Hz, 1H, CH), 2.98 – 2.89 (m, 1H, CH_2), 2.79 (dd, J_1 = 4.9 Hz, J_2 = 2.9 Hz, 1H, CH_2) ppm; ^{13}C NMR (101 MHz, $CDCl_3$) δ 158.57, 147.27, 132.70, 132.66, 132.28, 131.46, 128.36, 125.66, 122.99, 119.89, 119.55, 117.33, 116.83, 69.68, 50.54, 45.03 ppm; MS (ESI$^-$) m/z = 352.3 ([M-H]$^-$).

4-((2-Nitrophenyl)amino)phenol (18). To a stirred solution of 1-fluoro-2-nitrobezene (**17**, 1.62 g, 11.45 mmol, 1.21 mL) and $NaHCO_3$ (1.92 g, 22.91 mmol) in EtOH (20 mL), 4-aminophenol (1.50 g, 13.75 mmol) was added and the mixture was stirred at 100 °C for 20 h. After reaction mixture was cooled to r.t., $NaHCO_3$ was filtered off and the solvent was removed under reduced pressure. The crude product was purified with flash column chromatography using dichloromethane/methanol = 40:1) as eluent to obtain **18** (1.76 g).

Yield: 66.7 %; red crystals (1.76 g); ^{1}H NMR (400 MHz, $CDCl_3$) δ 9.39 (s, 1H, NH), 8.19 (dd, J_1 = 8.6 Hz, J_2 = 1.6 Hz, 1H, Ar-H), 7.33 (dddd, J_1 = 8.6 Hz, J_2 = 6.9 Hz, J_3 = 1.6 Hz, J_4 = 0.6 Hz, 1H, Ar-H), 7.21 – 7.08 (m, 2H, 2 × Ar-H), 6.99 (dd, J_1 = 8.7 Hz, J_2 = 1.3 Hz, 1H, Ar-H), 6.95 – 6.84 (m, 2H, 2 × Ar-H), 6.72 (ddd, J_1 = 8.4 Hz, J_2 = 6.9 Hz, J_3 = 1.3 Hz, 1H, Ar-H), 4.86 (s, 1H, OH) ppm; HRMS (ESI$^+$) for $C_{12}H_{11}N_2O_3$ ([M+H]$^+$) calculated 229.0619, found 229.0613.

4-((4-(Trifluoromethyl)phenyl)amino)phenol (21). Into an argon flushed sealed tube filled with solution of 4-aminophenol (116 mg, 1.07 mmol) and NaO*t*-Bu (234 mg, 2.22 mmol) in 1,4-dioxan (3 mL), compound **20** (200 mg, 0.889 mmol, 0.125 mL), BrettPhos (1.61 mg, 0.003 mmol) and $Pd(dba_3)_2$ (8.13 mg, 0.009 mmol) were successively added. Before sealing, the tube was filled with argon and then reaction mixture was vigorously stirred at 90 °C for 12 h. The reaction mixture was then cooled to room temperature and water (5 mL) was added. Water phase was extracted with ethyl acetate (2 × 10 mL), concentrated and then purified with flash column chromatography using ethyl acetate/hexane = 1:4 as eluent to obtain **21** (149 mg).

Yield: 66.2 %; brown crystals (149 mg); mp: 61.3-62.7 °C; ^{1}H NMR (400 MHz, $CDCl_3$) δ 7.41 (d, J = 8.3 Hz, 2H, 2 × Ar-H), 7.10 – 7.04 (m, 2H, 2 × Ar-H), 6.88 – 6.80 (m, 4H, 4 × Ar-H), 5.71 (s, 1H, NH), 4.81 (s, 1H, OH) ppm; MS (ESI$^-$) m/z = 252.6 ([M-H]$^-$).

4-(Oxiran-2-ylmethoxy)-*N*-(4-(trifluoromethyl)phenyl)aniline (22). Synthesised according to General procedure C with 5 equivalents of (±)-epichlorohydrin.

Yield: 61.5 %; yellowish crystals (112 mg); mp: 46.2-48.7 °C; ^{1}H NMR (400 MHz, $CDCl_3$) δ 7.42 (d, J = 8.4 Hz, 2H, 2 × Ar-H), 7.14 – 7.07 (m, 2H, 2 × Ar-H), 6.95 – 6.89 (m, 2H, 2 × Ar-H), 6.87 (d, J = 8.4 Hz, 2H, 2 × Ar-H), 5.73 (s, 1H, NH), 4.24 (dd, J_1 = 11.0 Hz, J_2 = 3.1 Hz, 1H, CH_2), 3.96 (dd, J_1 = 11.0 Hz, J_2 = 5.7 Hz, 1H, CH_2), 3.37 (ddt, J_1 = 5.7 Hz, J_2 = 4.2 Hz, J_3 = 2.7 Hz, 1H, CH), 2.93 (dd, J_1 = 4.9 Hz, J_2 = 4.2 Hz, 1H, CH_2),

2.78 (dd, J_1 = 4.9 Hz, J_2 = 2.7 Hz, 1H, CH_2) ppm; HRMS (ESI+) for $C_{16}H_{15}F_3NO_2$ ([M+H]+) calculated 310.1049, found 310.1067.

4-(Oxiran-2-ylmethoxy)-*N*-phenylaniline (24). Synthesised according to General procedure C with 5 equivalents of (±)-epichlorohydrin and bought compound **23**.

Yield: 64.4 %; brown solid (168 mg); mp: 57.2-59.8 °C; ^{1}H NMR (400 MHz, $CDCl_3$) δ 7.29 – 7.18 (m, 2H, 2 × Ar-H), 7.09 – 7.03 (m, 2H, 2 × Ar-H), 6.94 – 6.81 (m, 5H, Ar-H), 5.51 (s, 1H, NH), 4.20 (dd, J_1 = 11.0 Hz, J_2 = 3.2 Hz, 1H, CH_2), 3.95 (dd, J_1 = 11.0 Hz, J_2 = 5.6 Hz, 1H, CH_2), 3.36 (ddt, J_1 = 5.6 Hz, J_2 = 4.2 Hz, J_3 = 2.7 Hz, 1H, CH), 2.91 (dd, J_1 = 4.9 Hz, J_2 = 4.2 Hz, 1H, CH_2), 2.76 (dd, J_1 = 4.9 Hz, J_2 = 2.7 Hz, 1H, CH_2) ppm; MS (ESI+) m/z = 242.2 ([M+H]+).

2-((4-Hydroxyphenyl)amino)benzoic acid (26). To an argon flushed solution of 2-bromobenzoic acid (4.00g, 19.90 mmol) in 2-ethoxyethanol (20 mL), CuO (63 mg, 0.80 mmol), Cu (114 mg, 1.79 mmol), K_2CO_3 (5.50 g, 39.80 mmol) and 4-aminophenol (2.28 g, 20.89 mmol) were added and reaction mixture stirred at 130 °C for 16 h. Reaction mixture was then was cooled and water (35 mL) was added. Reaction mixture was filtered through Celite and then pH was adjusted to 5. The precipitated black solid was filtered off and dissolved in the 5 % Na_2CO_3 solution (35 mL). The solution was once more filtered through Celite and then pH was adjusted to 5. Precipitated black product was dried under reduced pressure and used in the next step without further purification.

Yield: 50.0 %; black solid (2.28 g); ^{1}H NMR (400 MHz, DMSO-d_6) δ 12.89 (s, 1H), 9.38 (d, J = 31.2 Hz, 2H), 7.84 (dd, J_1 = 8.0 Hz, J_2 = 1.7 Hz, 1H), 7.30 (ddd, J_1 = 8.7 Hz, J_2 = 7.1 Hz, J_3 = 1.7 Hz, 1H), 7.10 – 7.01 (m, 2H), 6.86 (dd, J_1 = 8.5 Hz, J_2 = 1.0 Hz, 1H), 6.84 – 6.77 (m, 2H), 6.65 (ddd, J_1 = 8.0 Hz, J_2 = 7.0 Hz, J_3 = 1.1 Hz, 1H) ppm.

Methyl 2-((4-hydroxyphenyl)amino)benzoate (27). Compound **26** (2.28 g, 9.96 mmol) was dissolved in anhydrous methanol (100 mL), cooled to 0 °C on an ice bath, and then thionyl chloride (11.85 g, 99.59 mmol, 7.22 mL) was slowly added. Reaction mixture was stirred overnight at 70 °C. The solvent was removed under reduced pressure, remaining residue was purified with flash column chromatography using ethyl acetate/hexane = 1:4 as eluent.

Yield: 54.9%; brown solid (1.33 g); ^{1}H NMR (400 MHz, $CDCl_3$) δ 9.23 (s, 1H), 7.94 (ddd, J_1 = 8.1 Hz, J_2 = 1.7 Hz, J_3 = 0.4 Hz, 1H), 7.27 – 7.24 (m, 1H), 7.15 – 7.09 (m, 2H), 6.96 (dd, J_1 = 8.6 Hz, J_2 = 1.1 Hz, 1H), 6.88 – 6.80 (m, 2H), 6.66 (ddd, J_1 = 8.1 Hz, J2 = 7.0 Hz, J_3 = 1.1 Hz, 1H), 4.92 (s, 1H), 3.90 (s, 3H) ppm; ^{13}C NMR (101 MHz, $CDCl_3$) δ 169.21, 152.80, 149.72, 134.33, 133.62, 131.67, 126.35, 116.32, 116.27, 113.47, 110.93, 51.85 ppm.

Methyl 2-((4-(oxiran-2-ylmethoxy)phenyl)amino)benzoate (28). Synthesised according to General procedure C from **27** (1.33 g, 5.47 mmol) with 10 equivalents of (±)-epichlorohydrin and purified with flash column chromatography using ethyl acetate/hexane = 1:4 as eluent.

Yield: 12.0%; brownish solid (201 mg); ^{1}H NMR (400 MHz, $CDCl_3$) δ 9.28 (s, 1H), 7.94 (dd, J_1 = 8.1 Hz, J_2 = 1.6 Hz, 1H), 7.26 (s, 1H), 7.20 – 7.14 (m, 2H), 6.99 (dd, J_1 = 8.5 Hz, J_2 = 1.1 Hz, 1H), 6.96 – 6.88 (m, 2H), 6.67 (ddd, J_1 = 8.1 Hz, J_2 = 7.0 Hz, J_3 = 1.1 Hz, 1H), 4.23 (dd, J_1 = 11.1 Hz, J_2 = 3.2 Hz, 1H), 3.99 (s, 1H), 3.90 (s, 3H), 3.37 (ddt, J_1 = 5.8 Hz, J_2 = 4.1 Hz, J_3 = 2.9 Hz, 1H), 2.92 (dd, J_1 = 4.9 Hz, J_2 = 4.1 Hz, 1H), 2.77 (dd, J_1 = 5.0 Hz, J_2 = 2.7 Hz, 1H) ppm; ^{13}C NMR (101 MHz, $CDCl_3$) δ 169.16, 155.64, 149.49, 134.31, 134.15, 131.68, 125.88, 116.44, 115.65, 113.51, 111.08, 69.26, 51.85, 50.34, 44.89 ppm.

1-(Dimethylamino)-3-(4-((2-nitro-4-(trifluoromethyl)phenyl)amino)phenoxy)propan-2-ol (ZVS-08). Synthesized according to General procedure B with 3.6 equivalents of Me_2NH × HCl. Product was

additionally purified with flash column chromatography using acetonitrile/acetone/triethylamine = 1:1:0.01 as eluent.

Yield: 21.3 %; orange crystals (48 mg); mp: 78.3-81.2 °C; ^{1}H NMR (400 MHz, $CDCl_3$) δ 9.61 (s, 1H, NH), 8.50 (s, 1H, Ar-H), 7.49 (dd, J_1 = 9.0 Hz, J_2 = 2.2 Hz, 1H, Ar-H), 7.22 – 7.16 (m, 2H, 2 × Ar-H), 7.09 – 6.97 (m, 3H, 3 × Ar-H), 4.16 – 4.05 (m, 1H, CH), 4.02 (dd, J_1 = 4.9 Hz, J_2 = 2.2 Hz, 2H, CH_2), 2.59 (dd, J_1 = 12.2 Hz, J_2 = 9.9 Hz, 1H, CH_2), 2.42 (dd, J_1 = 12.2 Hz, J_2 = 3.7 Hz, 1H, CH_2), 2.36 (s, 6H, 2 × CH_3) ppm; ^{13}C NMR (101 MHz, $CDCl_3$) δ 158.43, 146.85, 132.22, 132.19, 132.16, 132.12, 131.73, 130.68, 127.94, 127.84, 125.23, 125.20, 125.16, 125.11, 122.52, 119.81, 119.67, 119.32, 118.98, 118.64, 116.87, 116.24, 70.80, 66.15, 61.70, 45.49 ppm; HRMS (ESI$^+$) for $C_{18}H_{21}N_3O_4F_3$ ([M+H]$^+$) calculated 400.1479, found 400.1473; HPLC retention time: 4.883 min (99.5 % at 254 nm).

^{1}H and ^{13}C Spectra of compounds

N-(4-(3-(dimethylamino)propoxy)phenyl)-2-nitro-4-(trifluoromethyl)aniline (1)

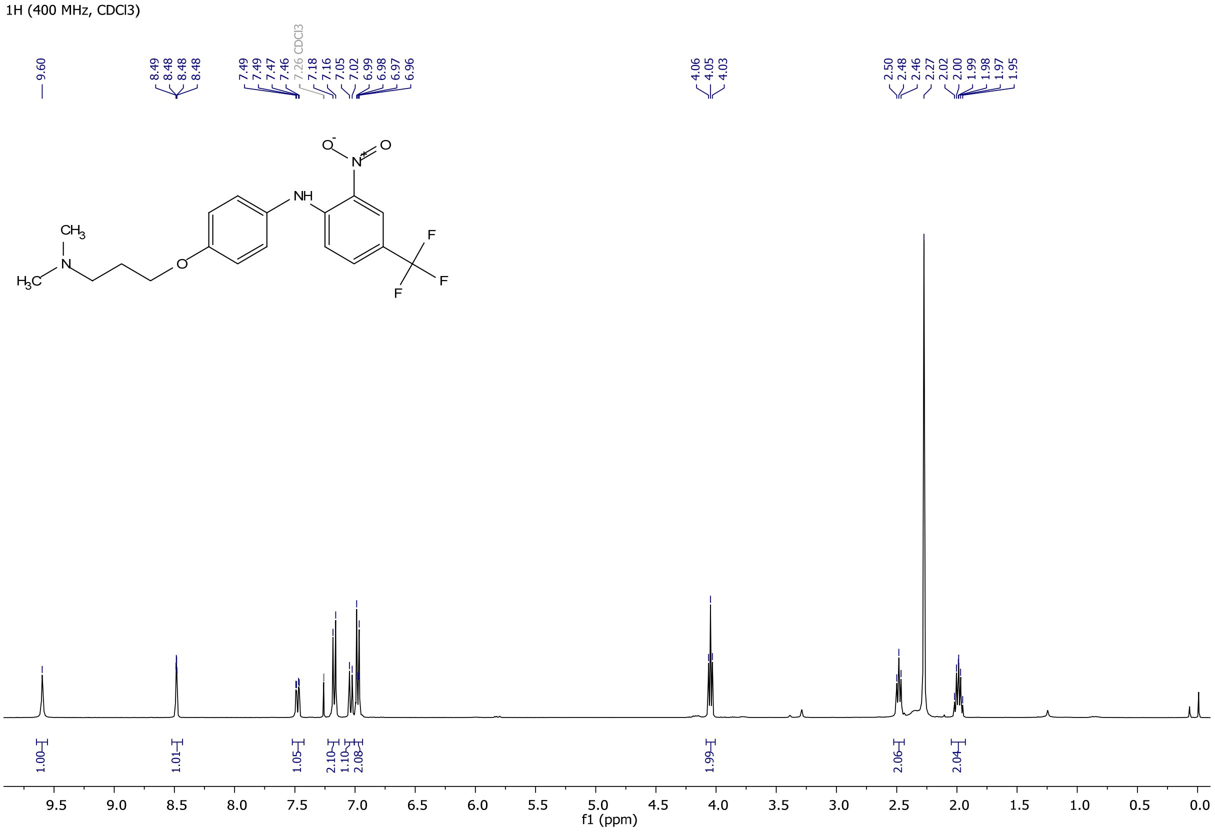

13C (101 MHz, CDCl3)

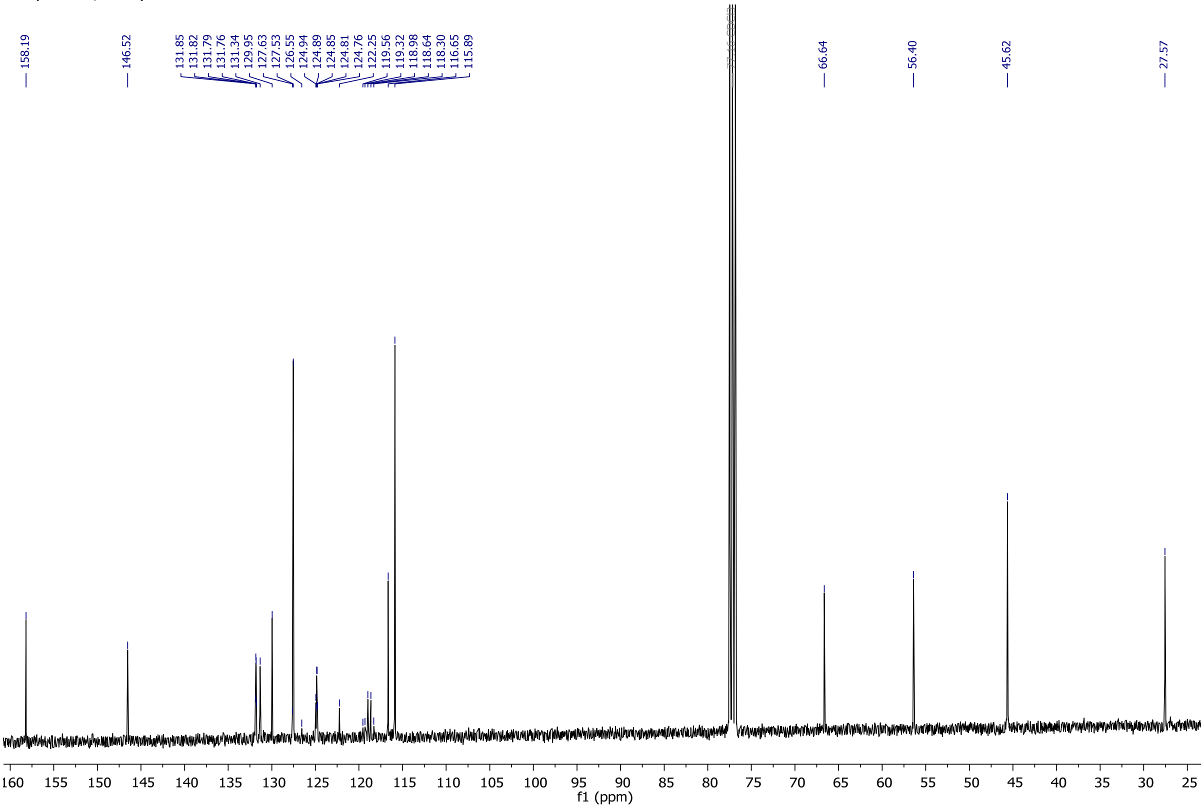

1-Morpholino-3-(4-((2-nitrophenyl)amino)phenoxy)propan-2-ol (2)

1H (400 MHz, CDCl3)

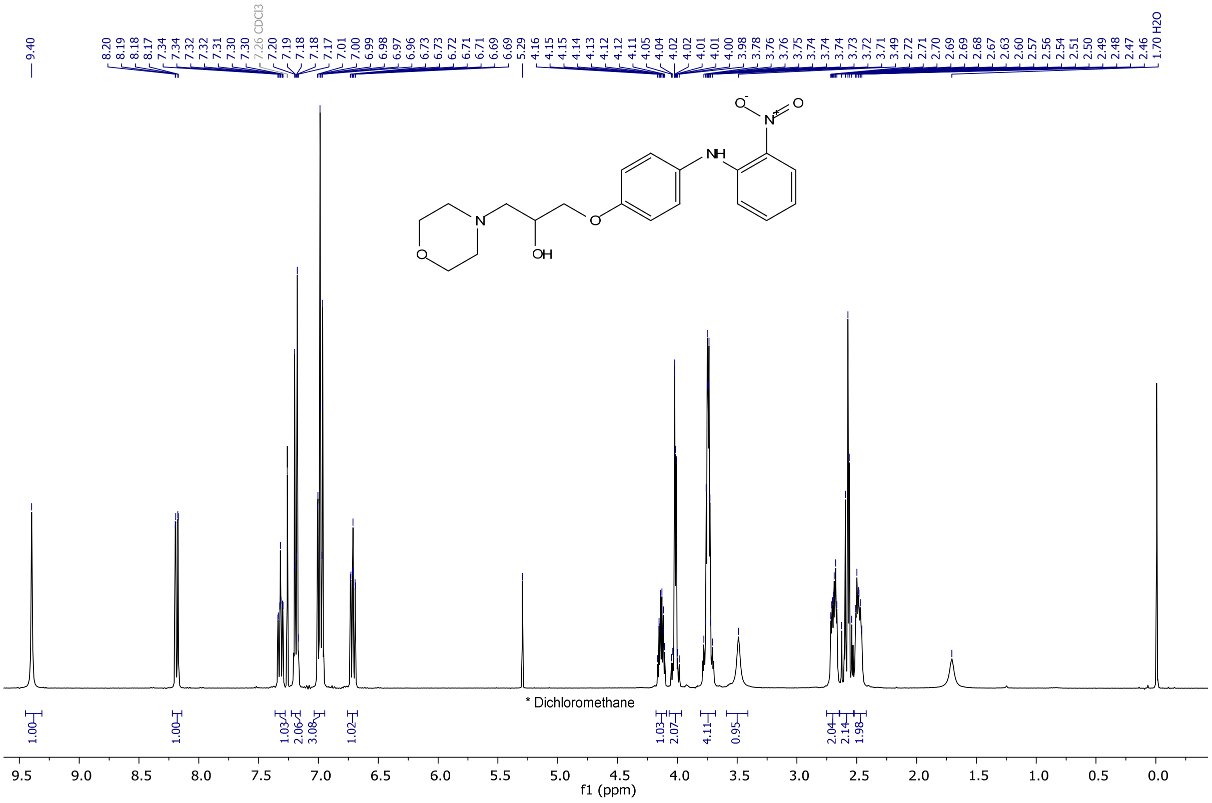

13C (101 MHz, CDCl3)

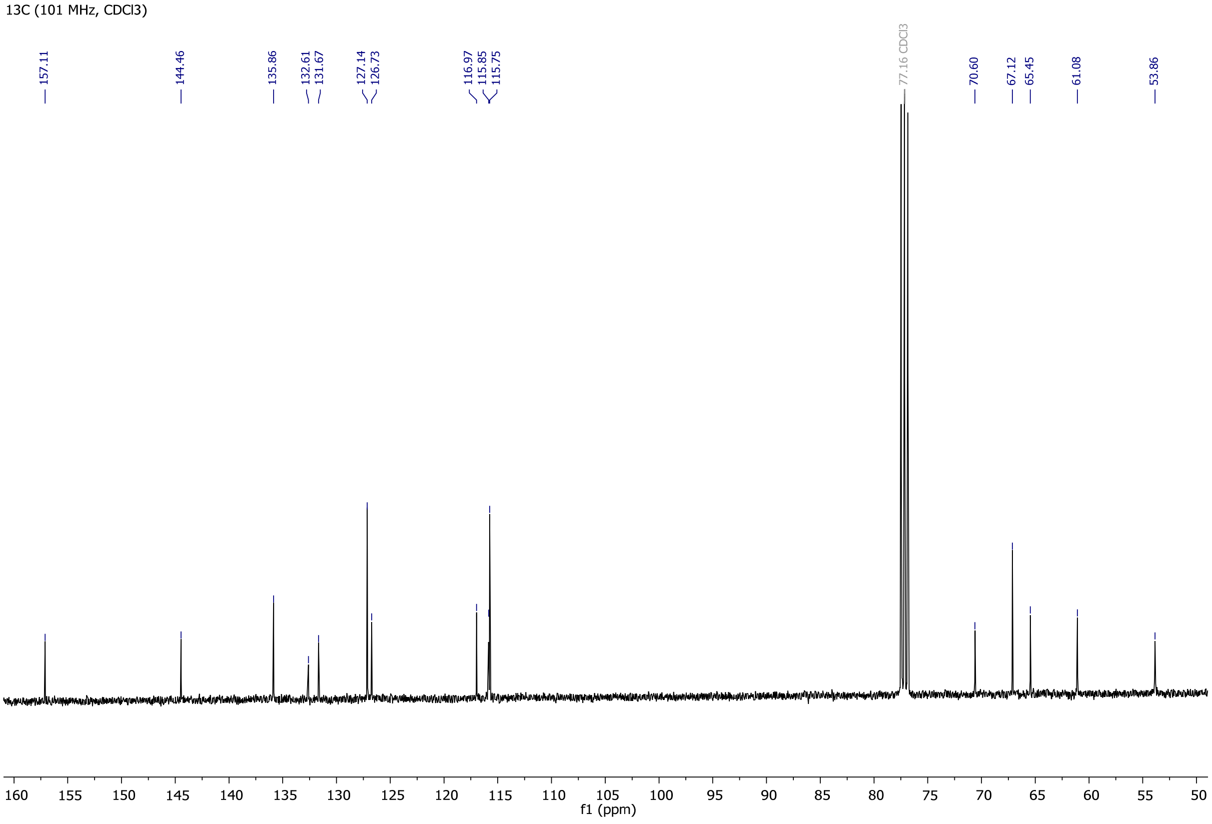

1-(Dimethylamino)-3-(4-(phenylamino)phenoxy)propan-2-ol (3)

1H (400 MHz, CDCl3)

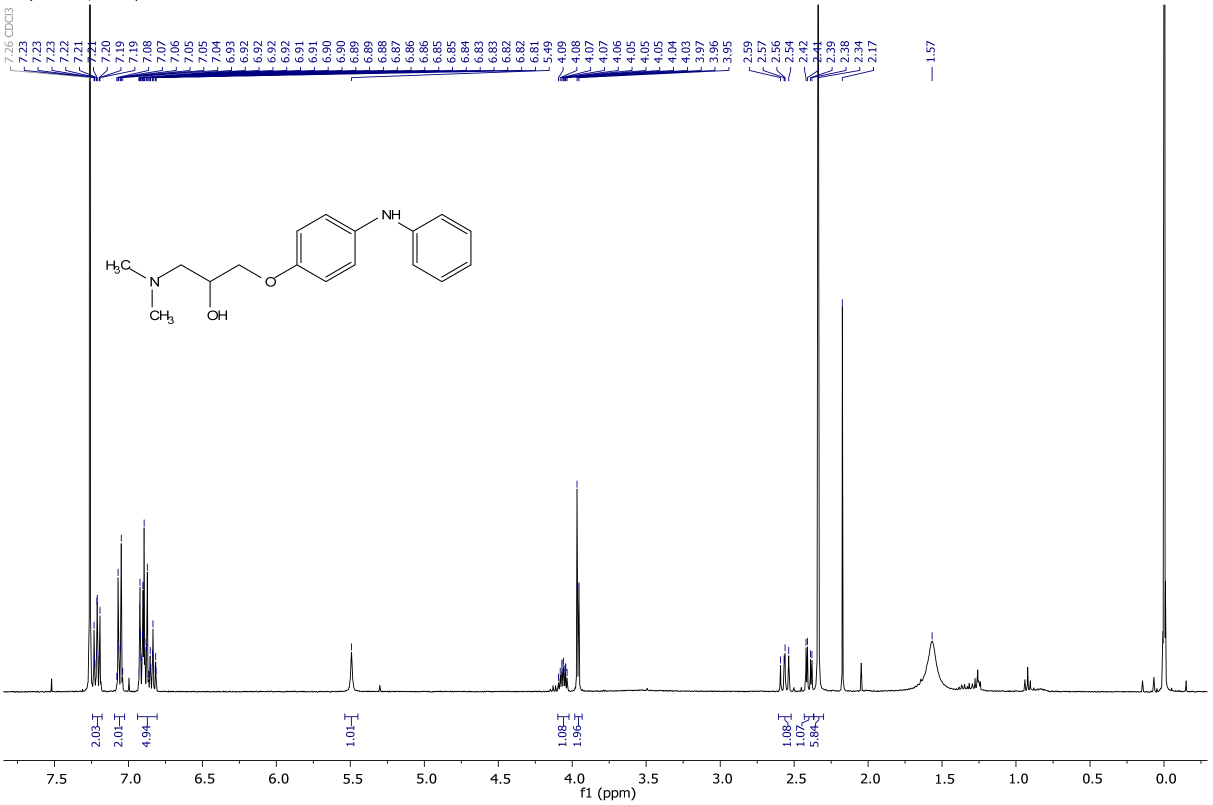

13C (101 MHz, CDCl3)

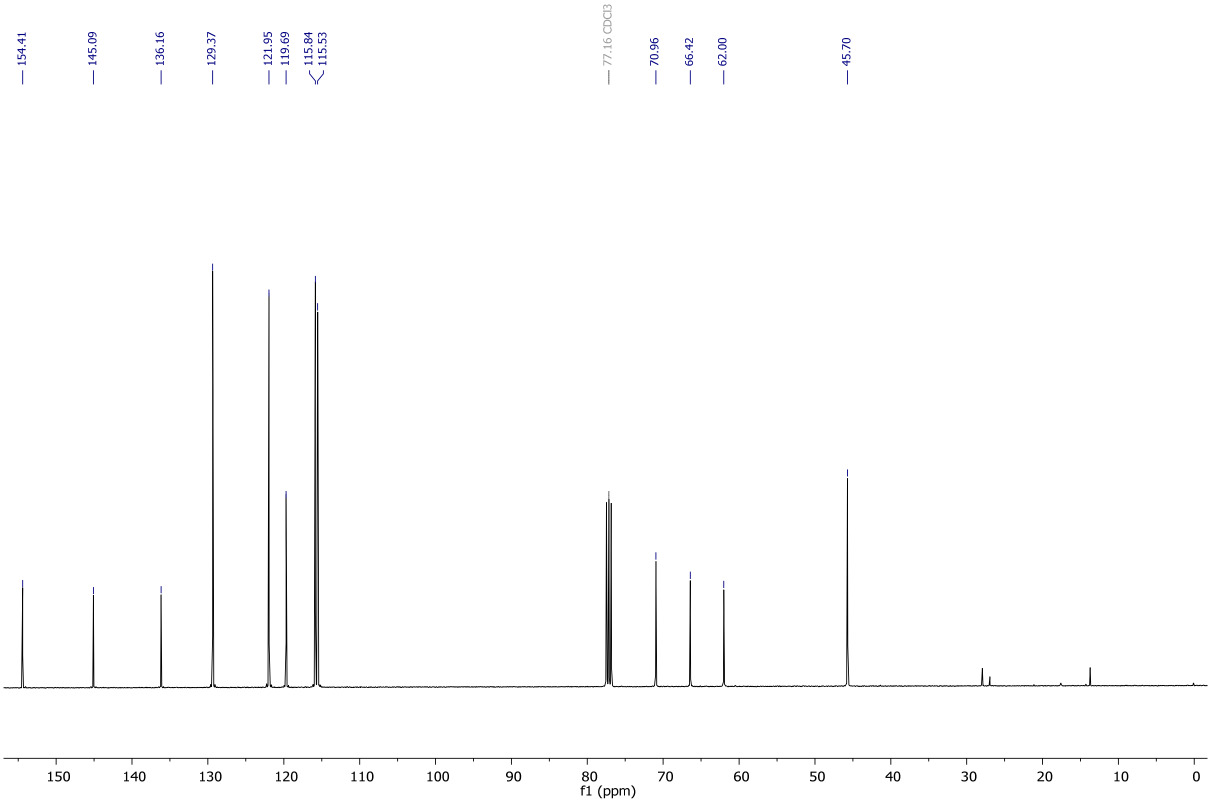

N-(4-(3-(dimethylamino)propoxy)phenyl)-2-nitroaniline (4)

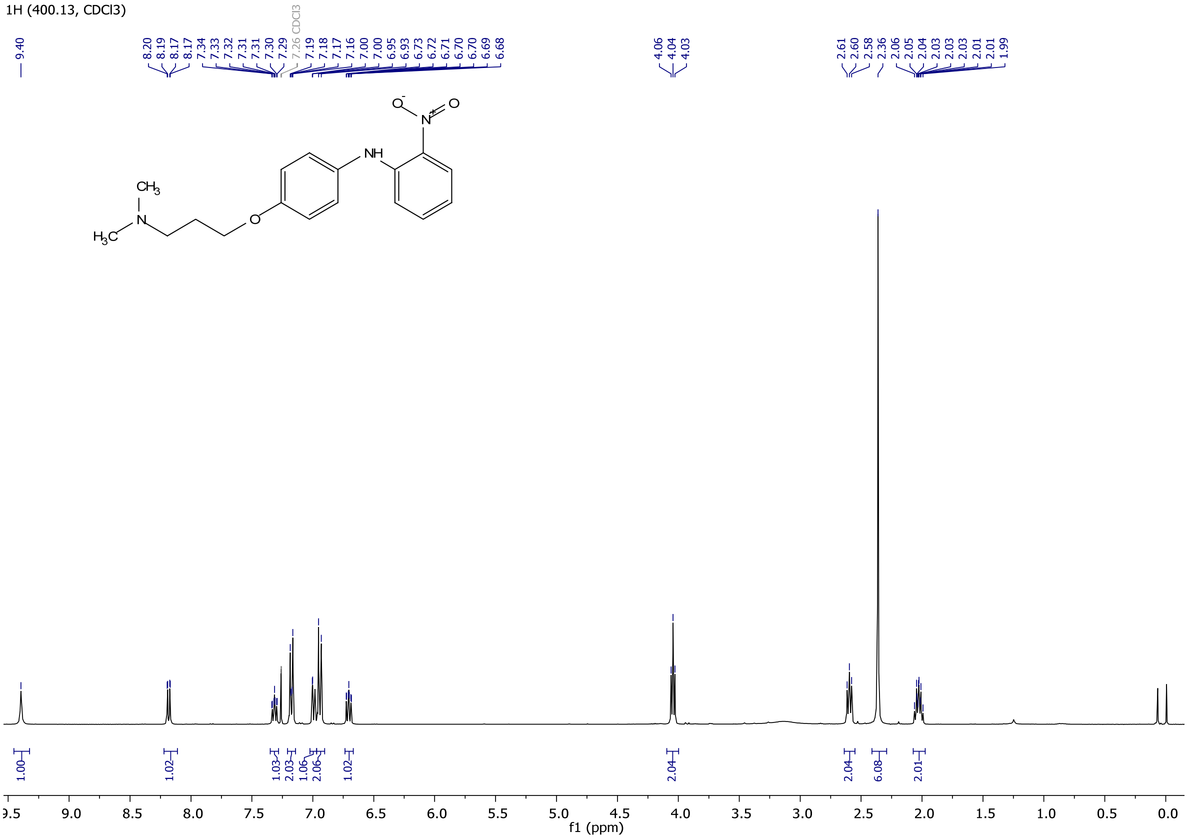

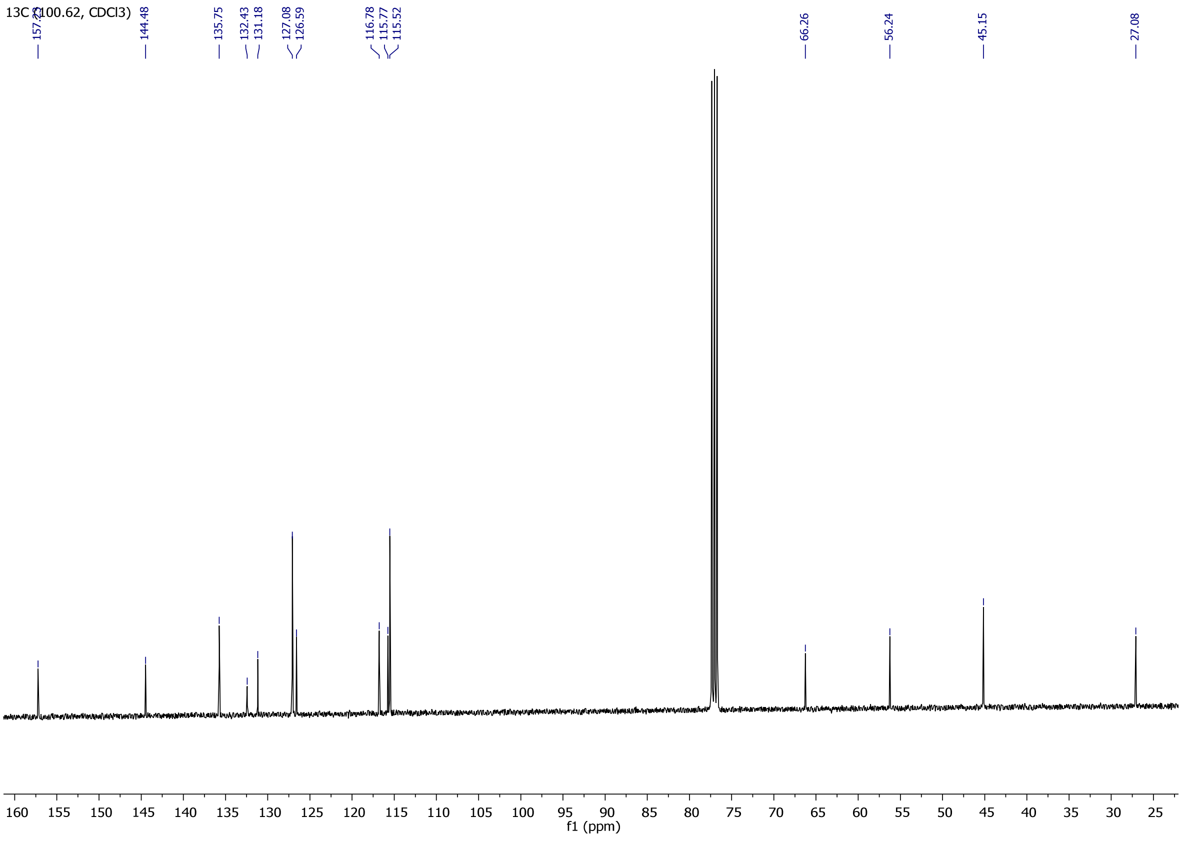

1-(4-((2-Nitro-4-(trifluoromethyl)phenyl)amino)phenoxy)-3-(phenylamino)propan-2-ol (5)

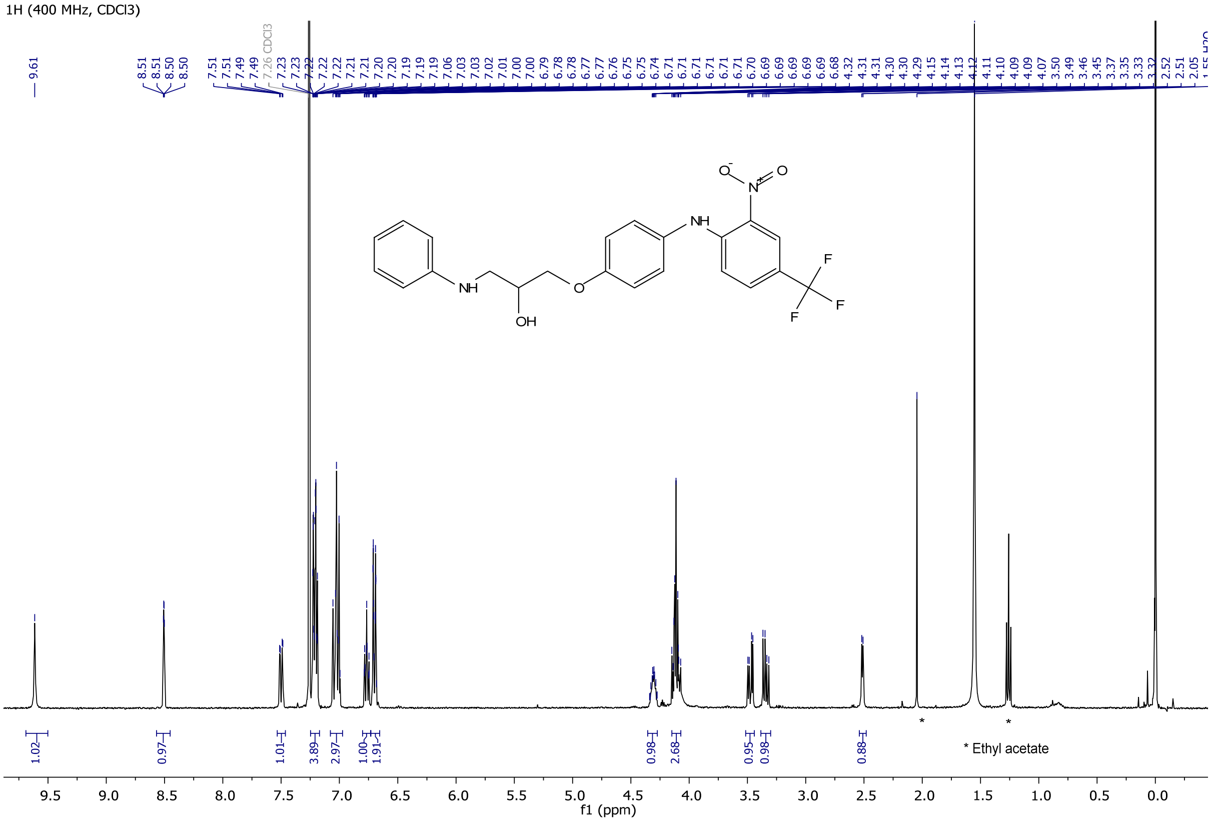

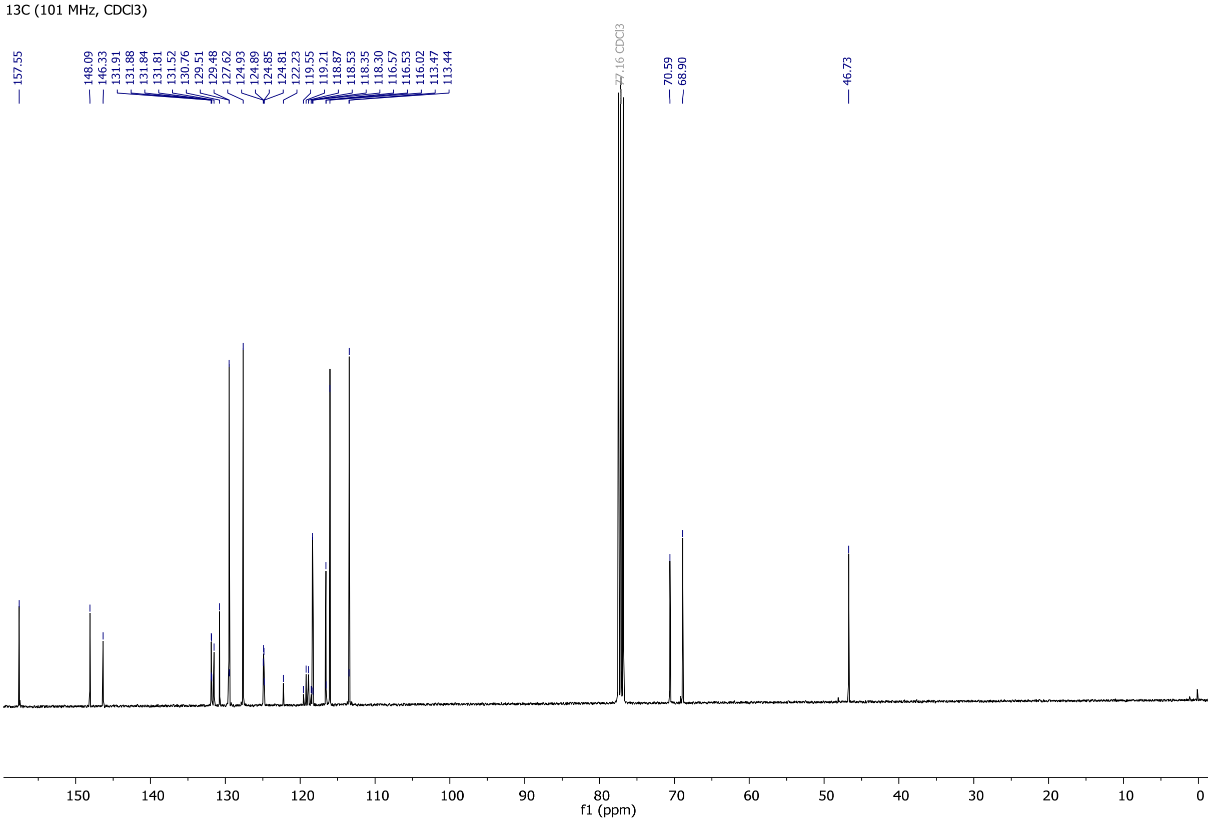

1-(Dimethylamino)-3-(4-((4-(trifluoromethyl)phenyl)amino)phenoxy)propan-2-ol (6)

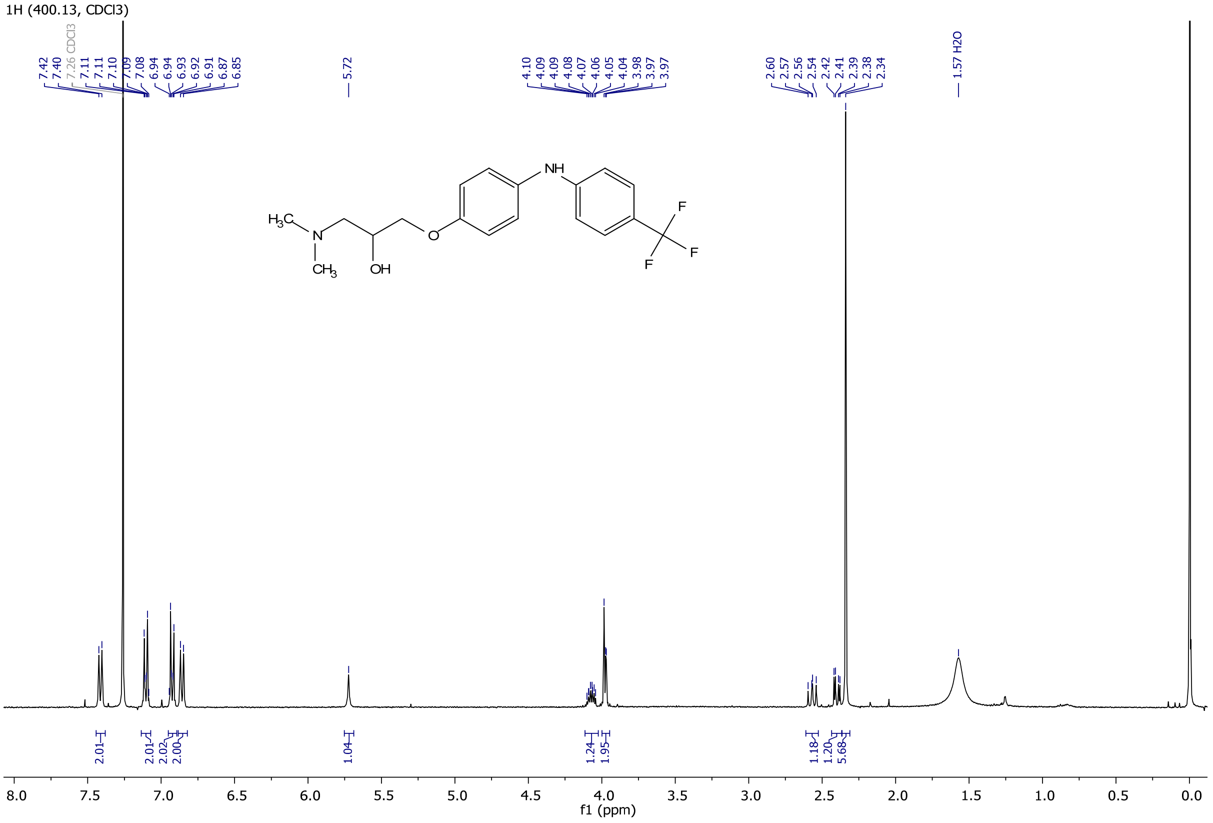

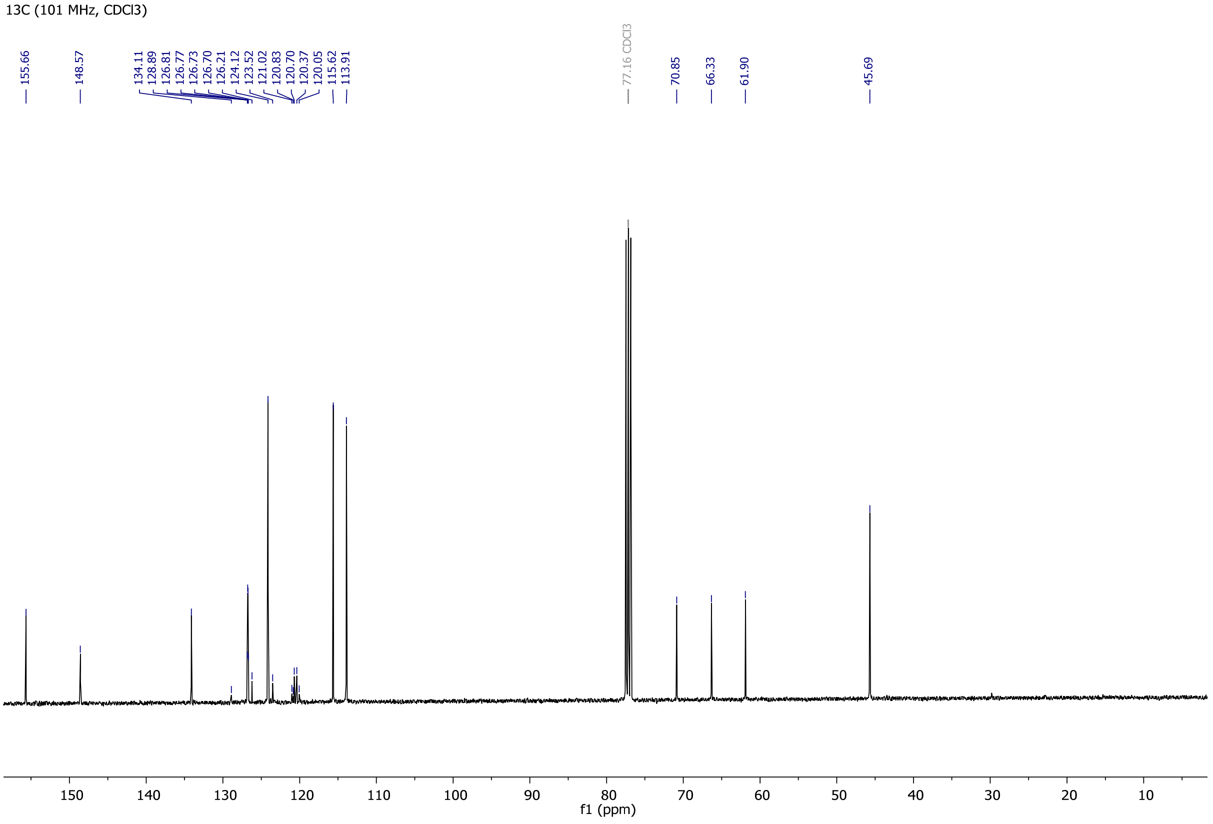

1-(Dimethylamino)-3-(4-((2-nitrophenyl)amino)phenoxy)propan-2-ol (7)

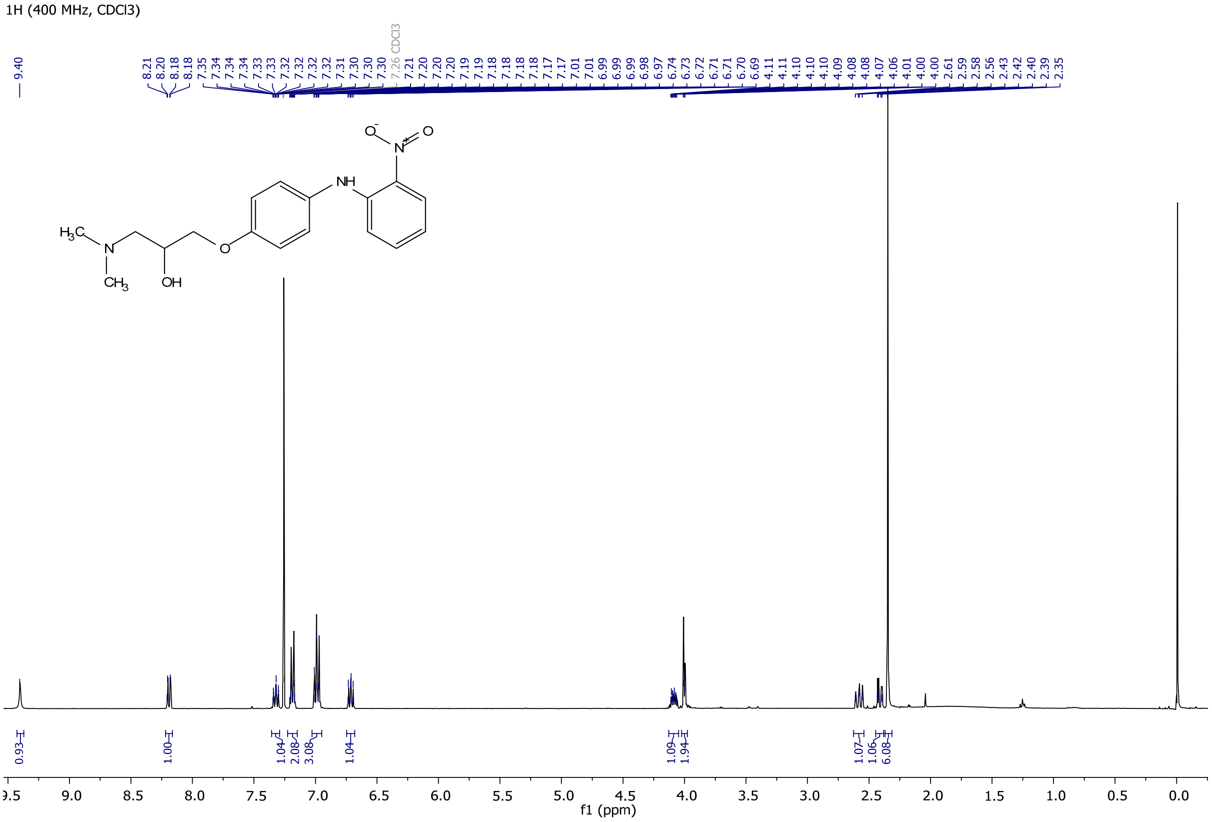

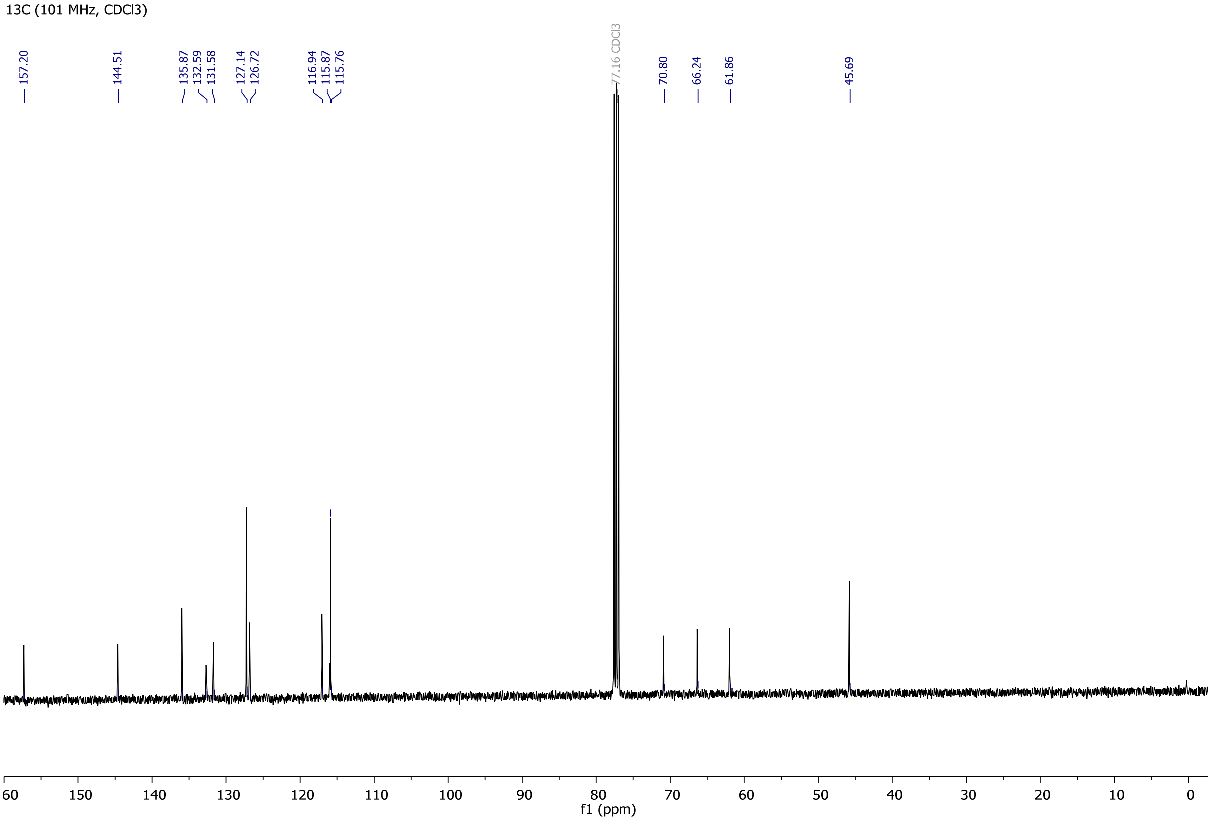

1-Morpholino-3-(4-((2-nitro-4-(trifluoromethyl)phenyl)amino)phenoxy)propan-2-ol (8)

1H (400 MHz, CDCl3)

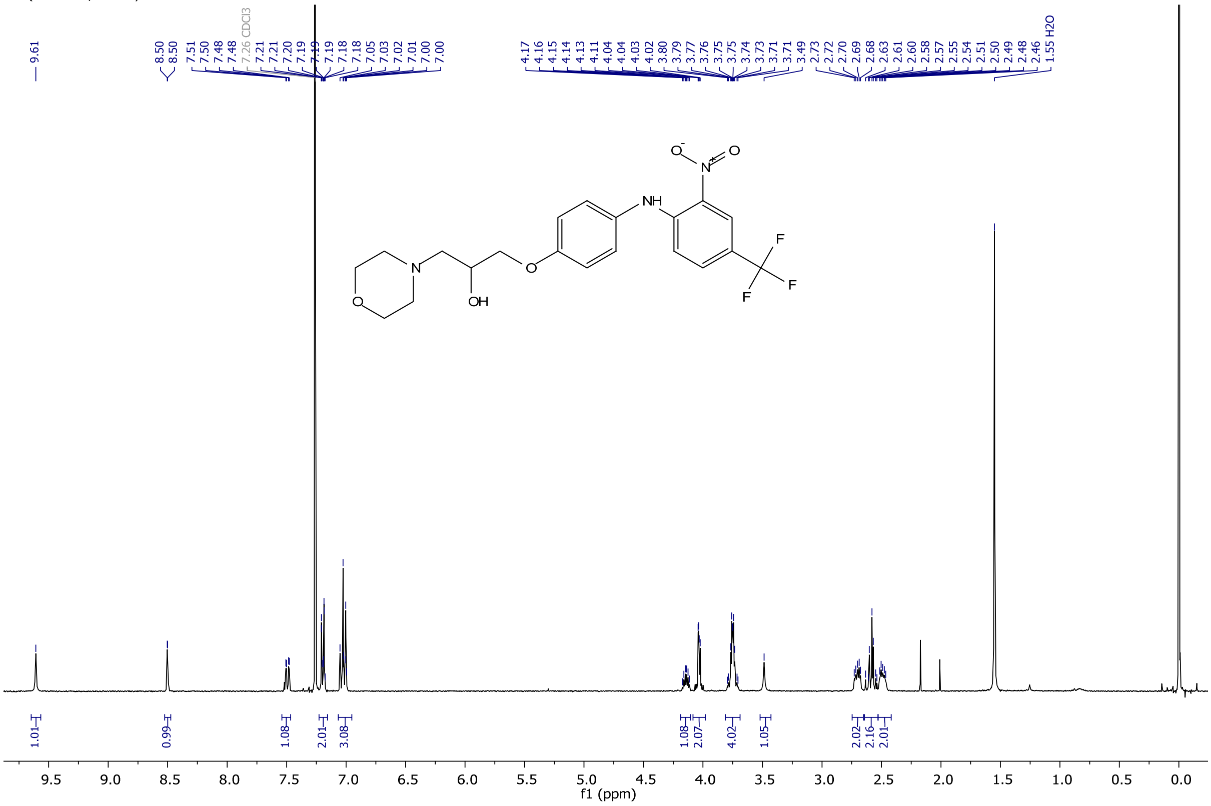

13C (101 MHz, CDCl3)

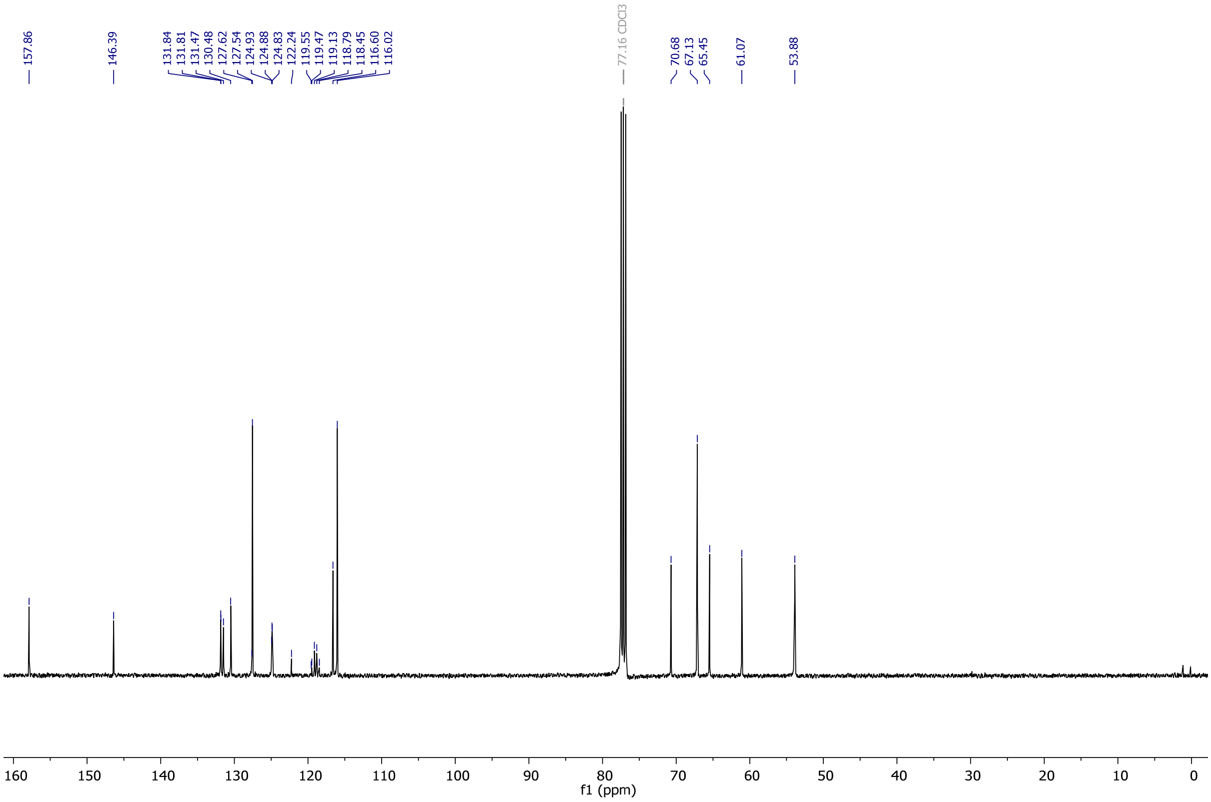

Methyl 2-((4-(3-(dimethylamino)-2-hydroxypropoxy)phenyl)amino)benzoate (9)

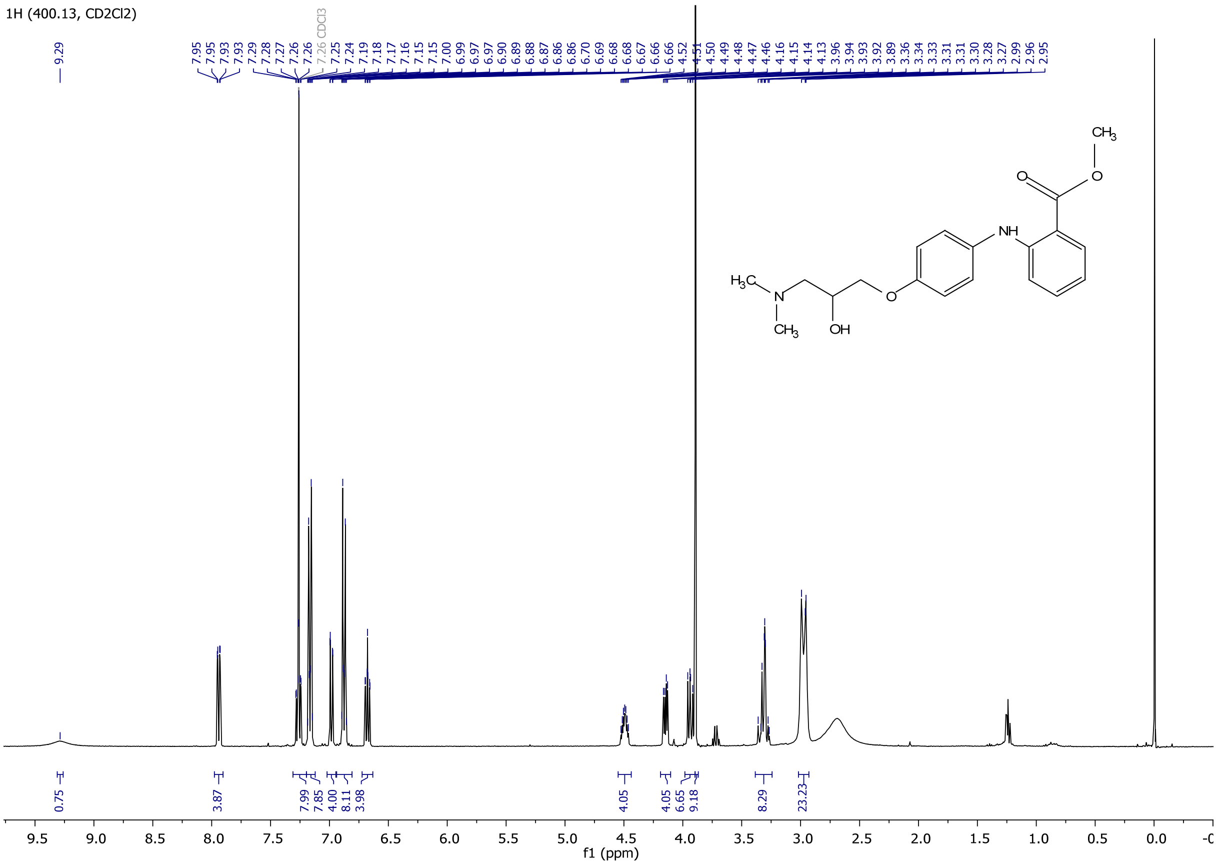

1-(4-((2-Amino-4-(trifluoromethyl)phenyl)amino)phenoxy)-3-(dimethylamino)propan-2-ol (<u>10</u>)

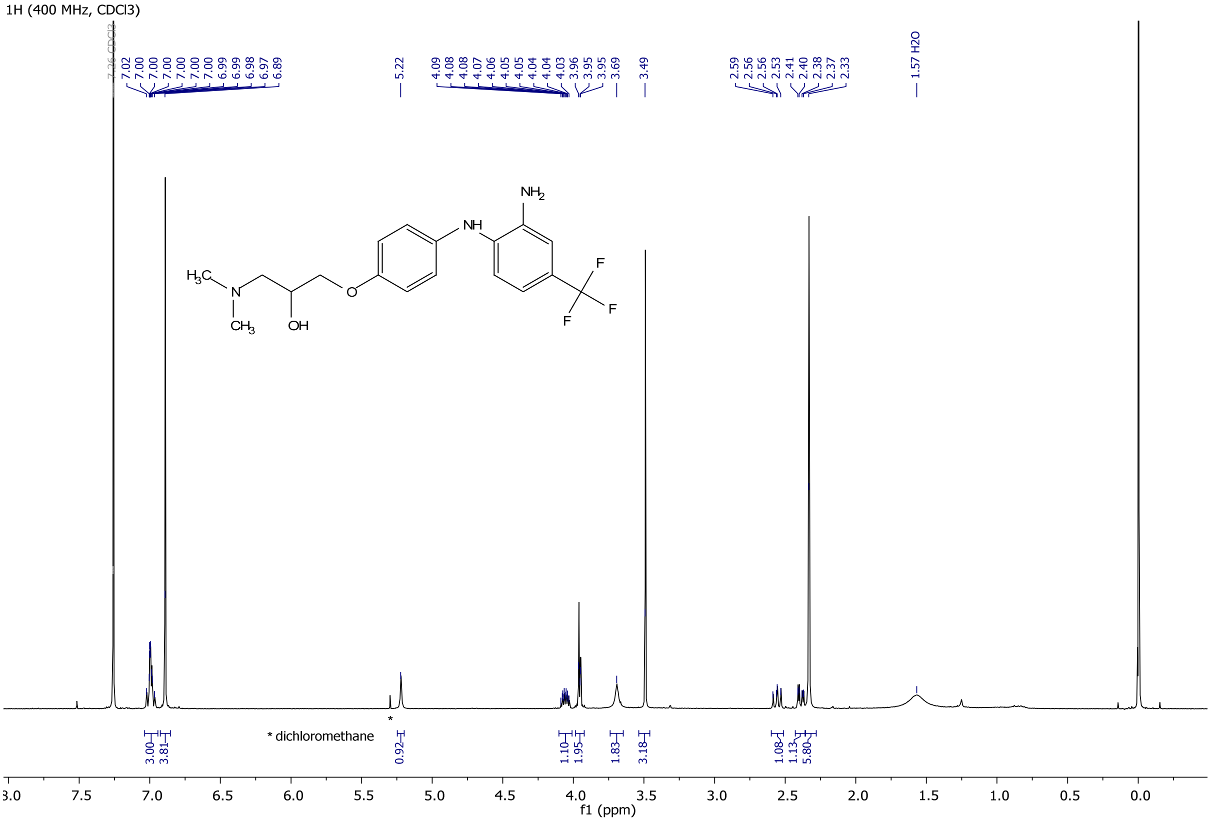

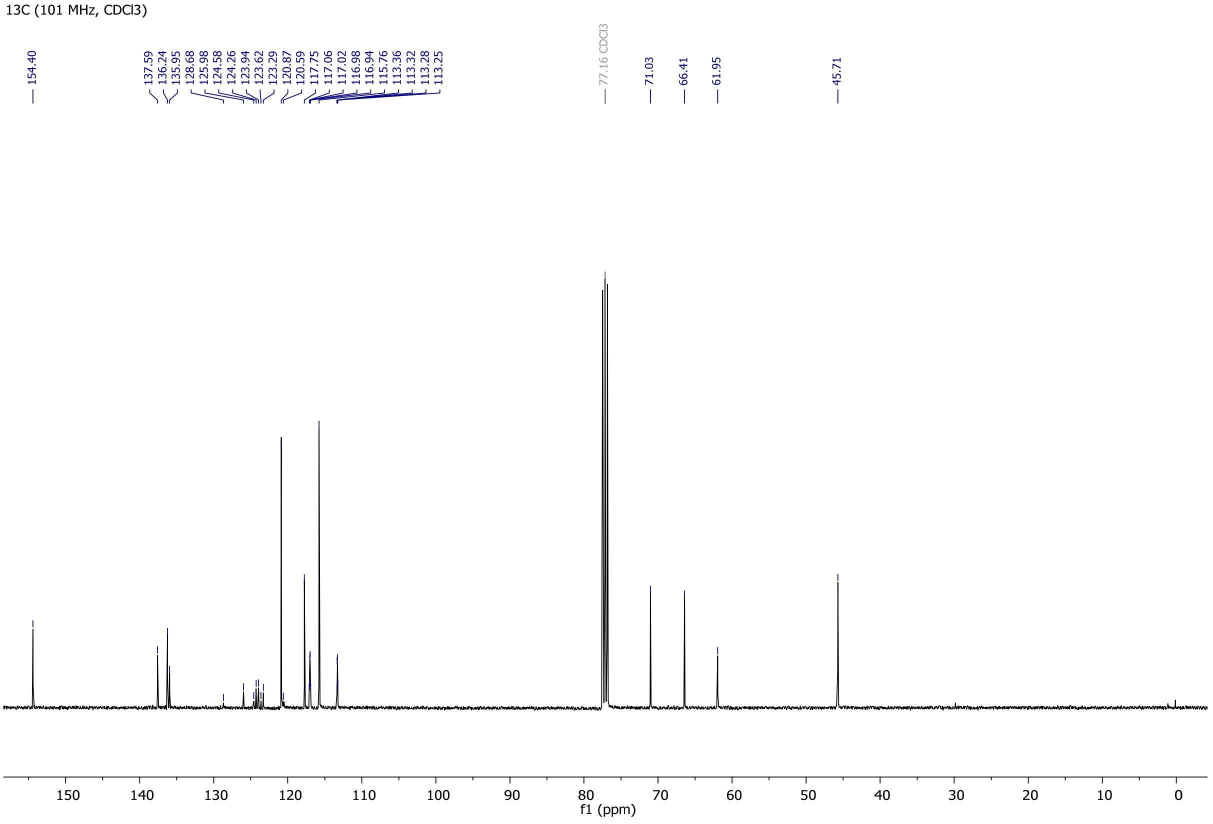

2-((4-(3-(Dimethylamino)-2-hydroxypropoxy)phenyl)amino)benzoic acid (11)

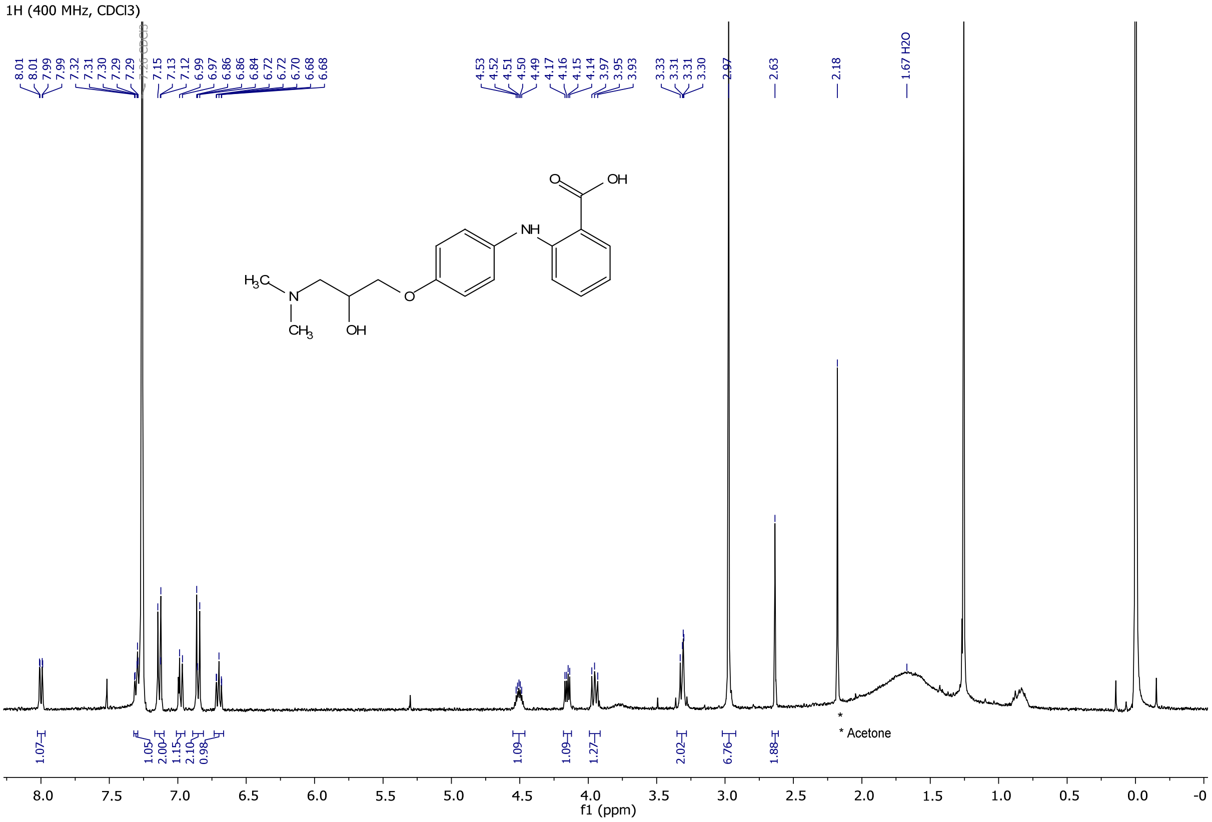

N-(2-hydroxy-3-(4-((2-nitro-4-(trifluoromethyl)phenyl)amino)phenoxy)propyl)acetamide (12)

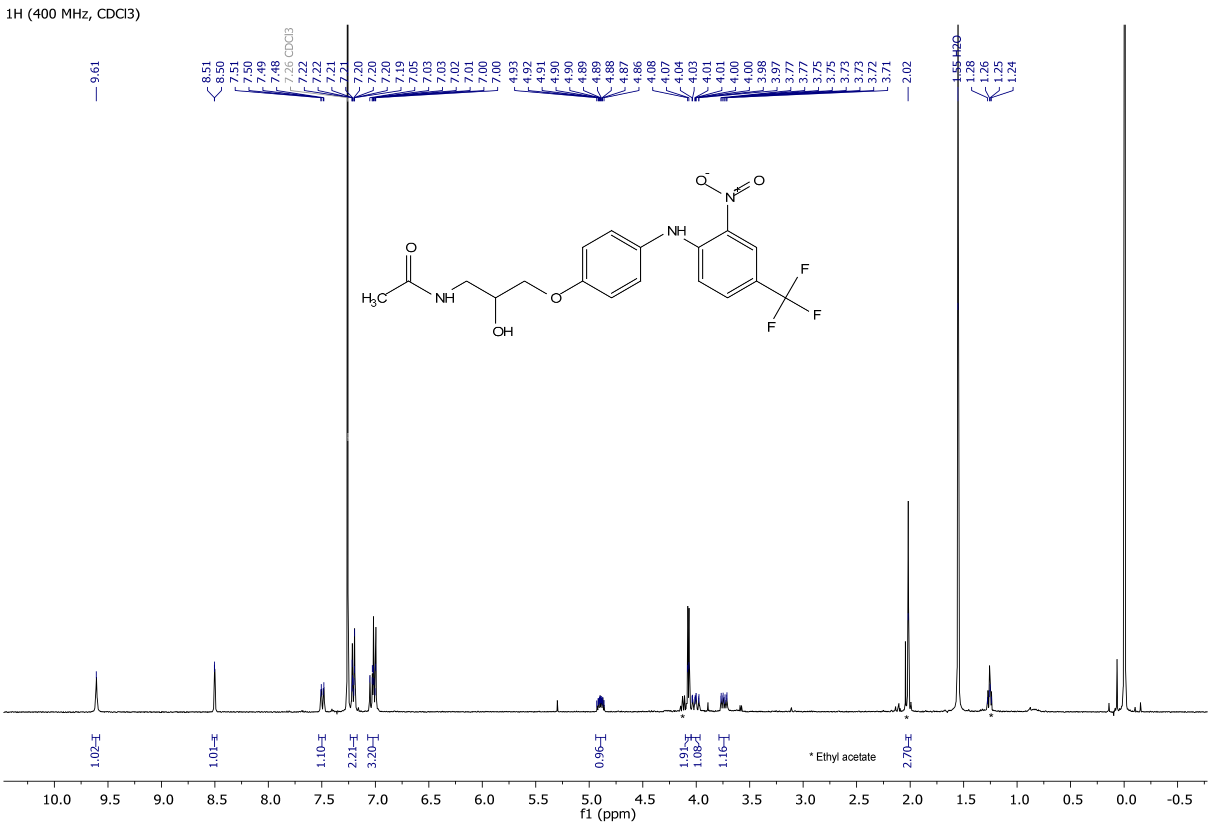

1-Chloro-2-nitro-4-(trifluoromethyl)benzene (14)

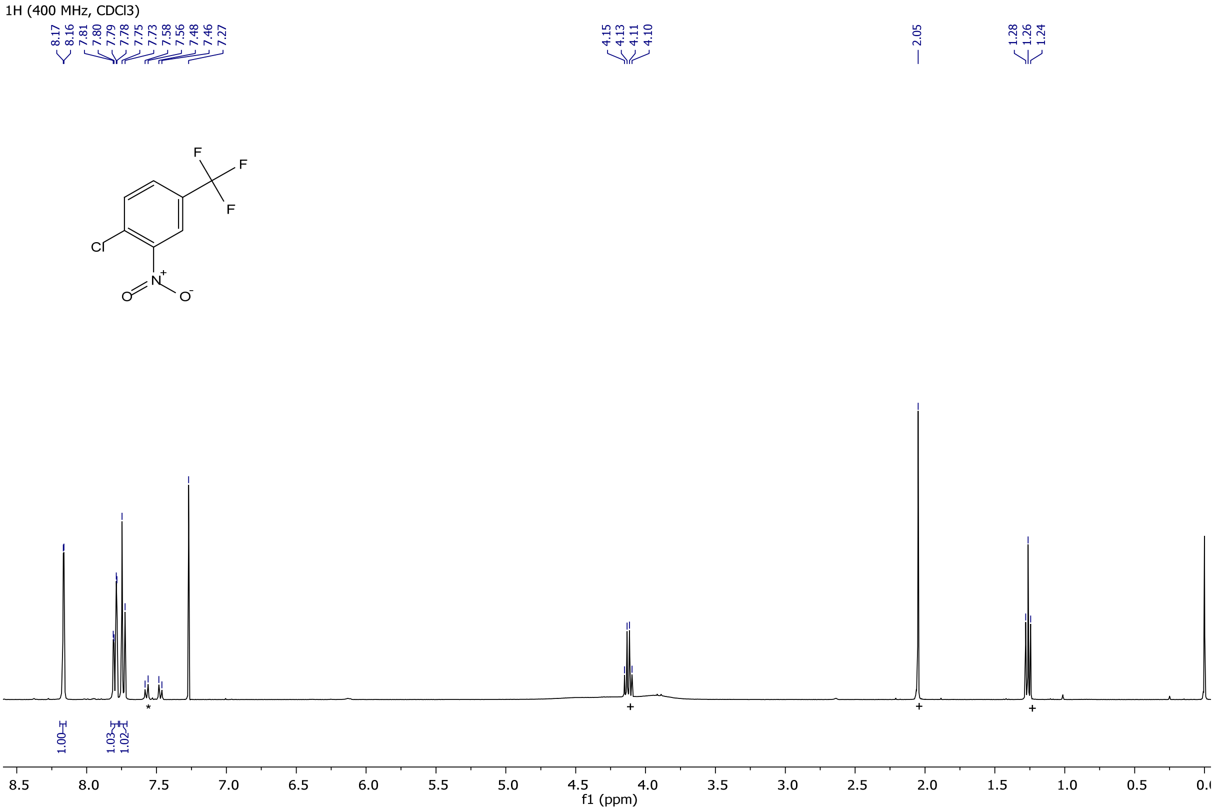

4-((2-Nitro-4-(trifluoromethyl)phenyl)amino)phenol (15)

1H (400 MHz, CDCl3)

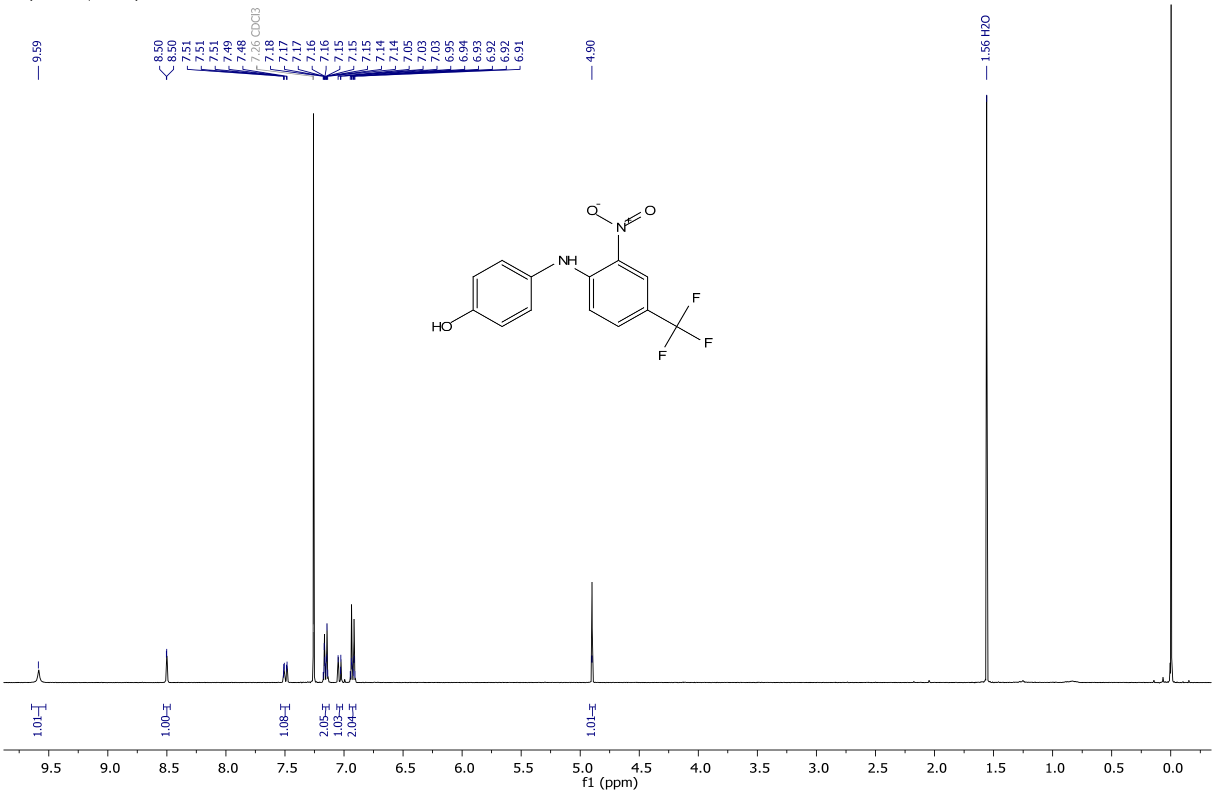

13C (101 MHz, CDCl3)

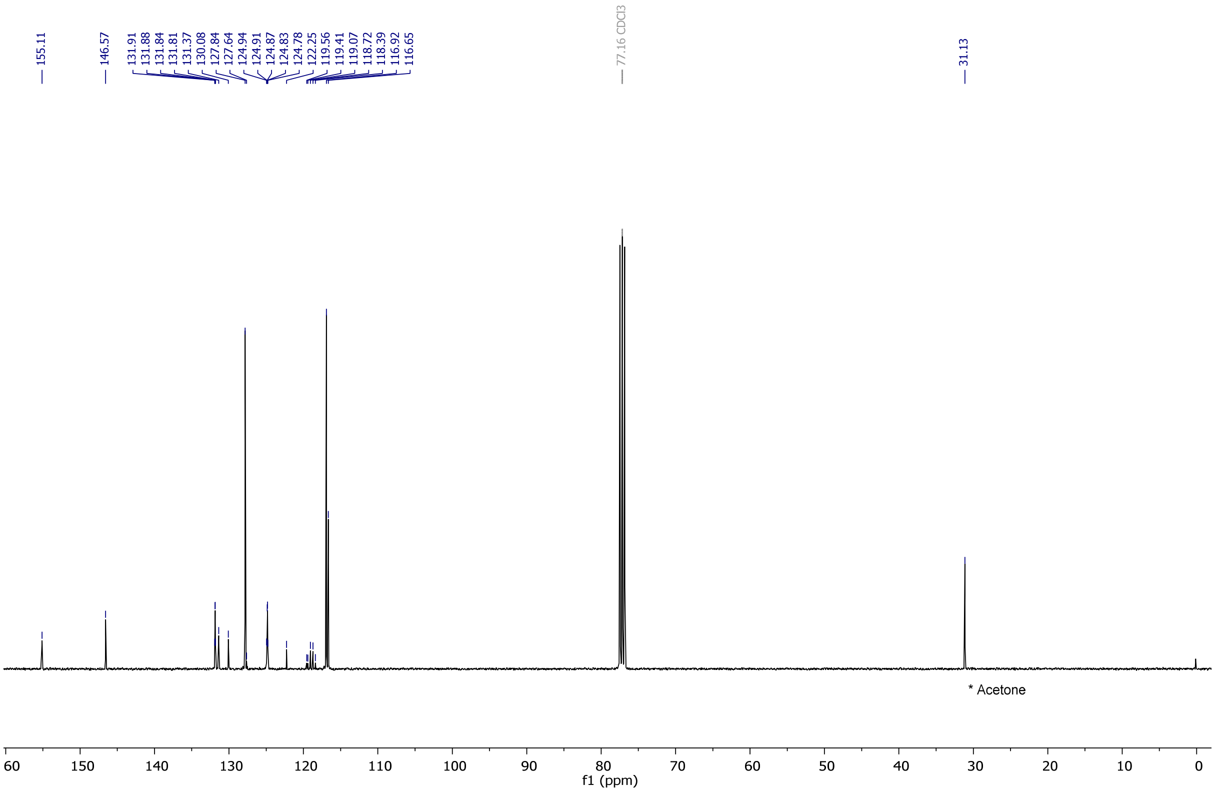

2-Nitro-*N*-(4-(oxiran-2-ylmethoxy)phenyl)-4-(trifluoromethyl)aniline (16)

1H (400 MHz, CDCl3)

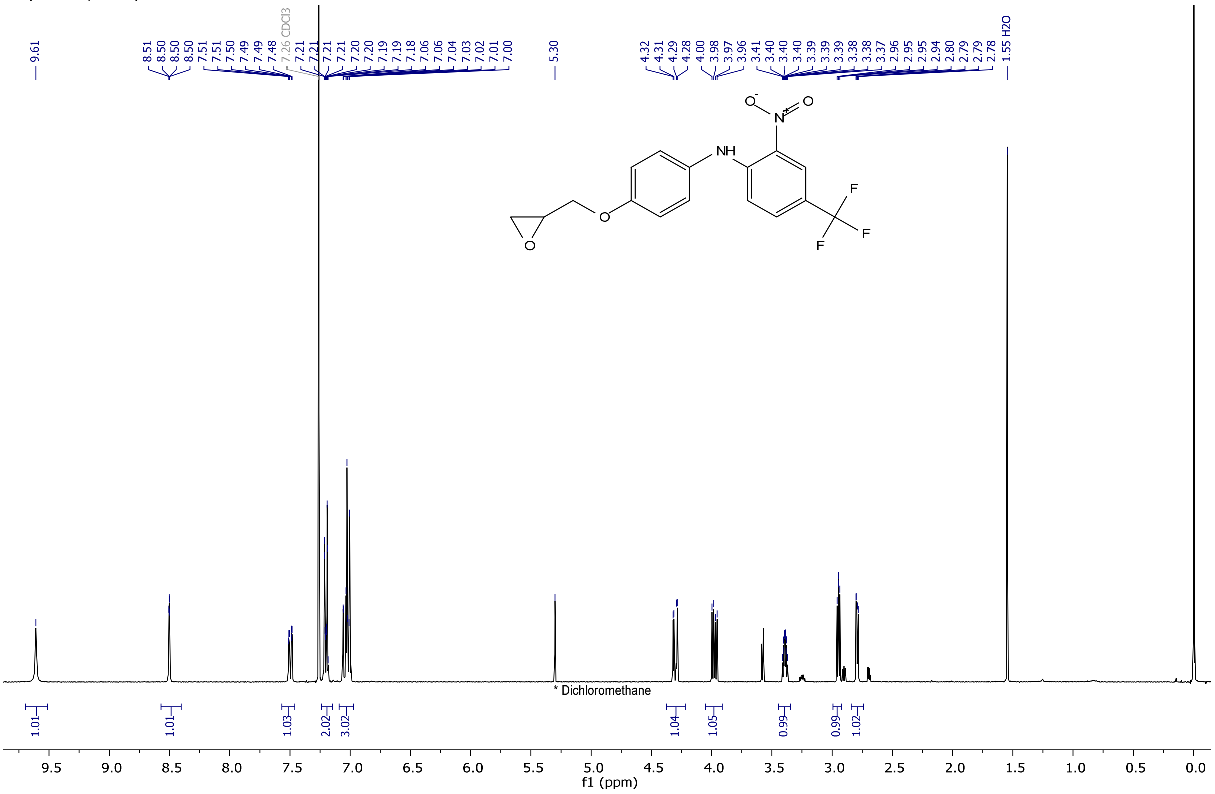

13C (100 MHz, CDCl3)

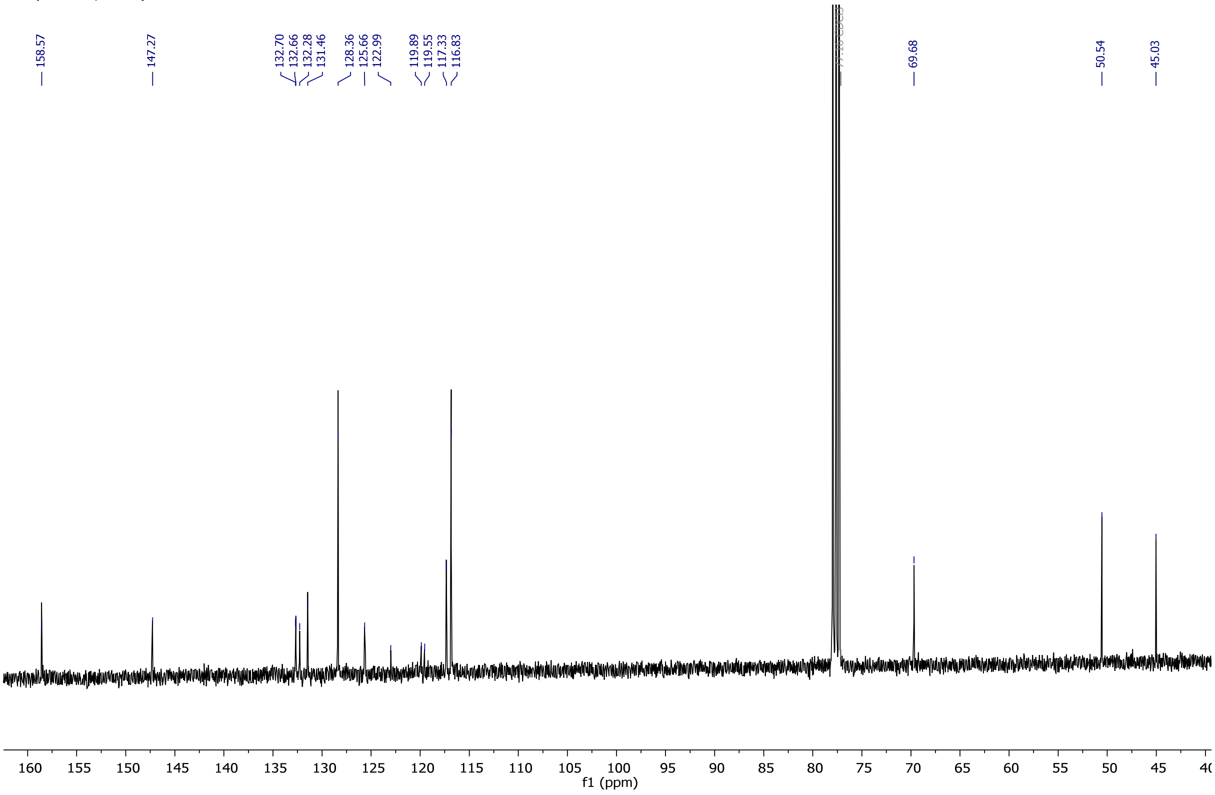

4-((2-Nitrophenyl)amino)phenol (<u>18</u>)

1H (400 MHz, CDCl3)

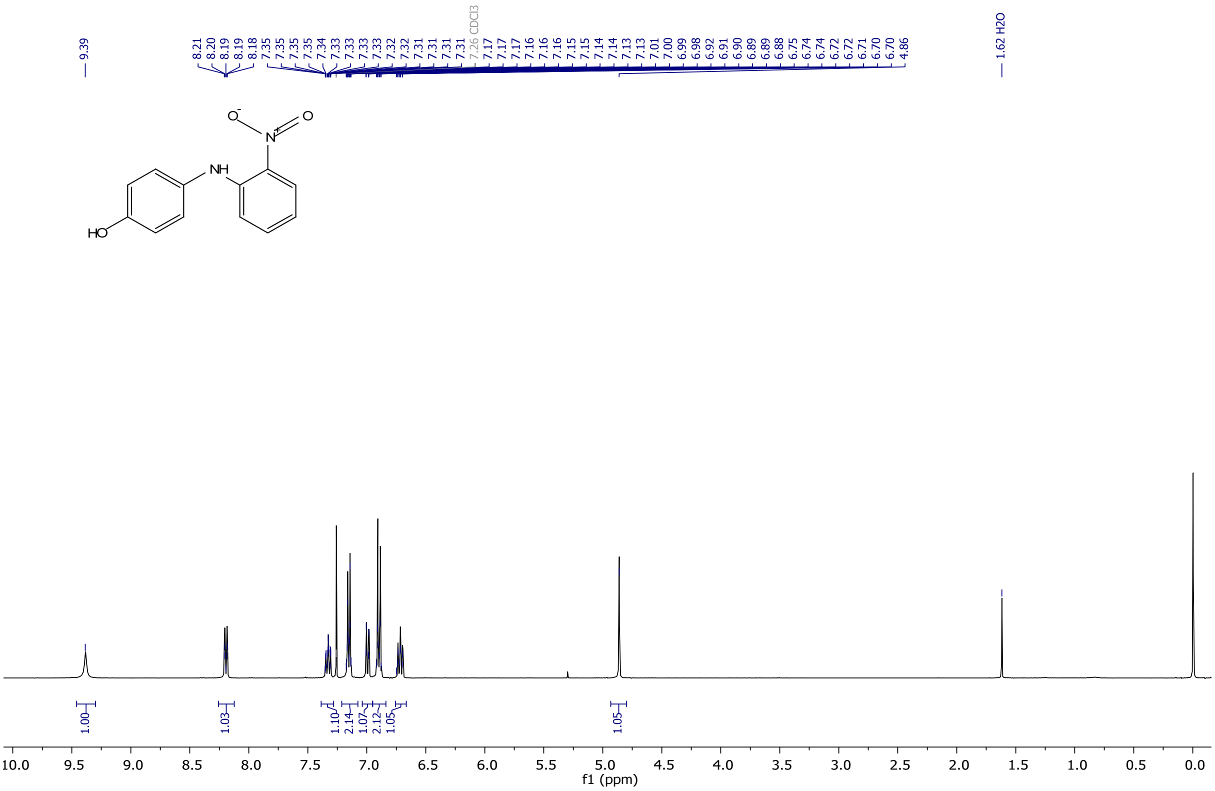

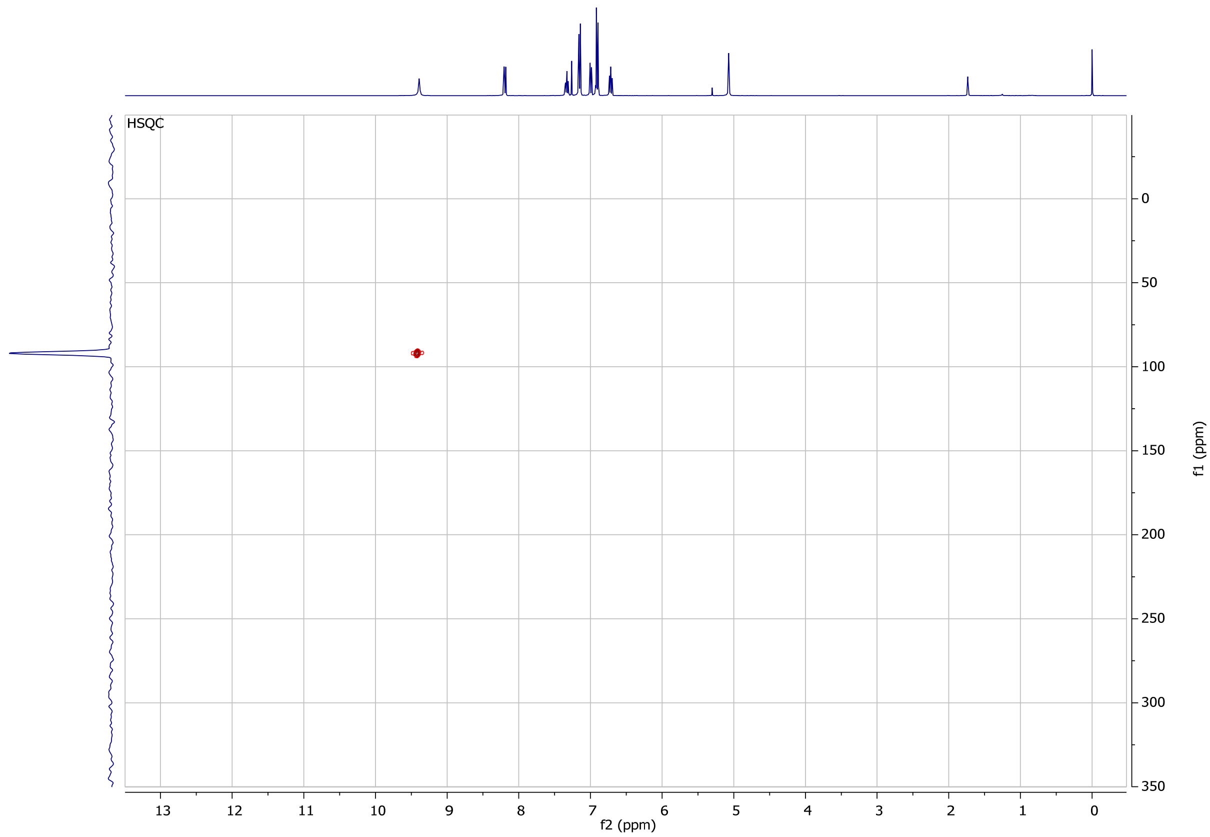

2-Nitro-*N*-(4-(oxiran-2-ylmethoxy)phenyl)aniline (19)

1H (400 MHz, CDCl3)

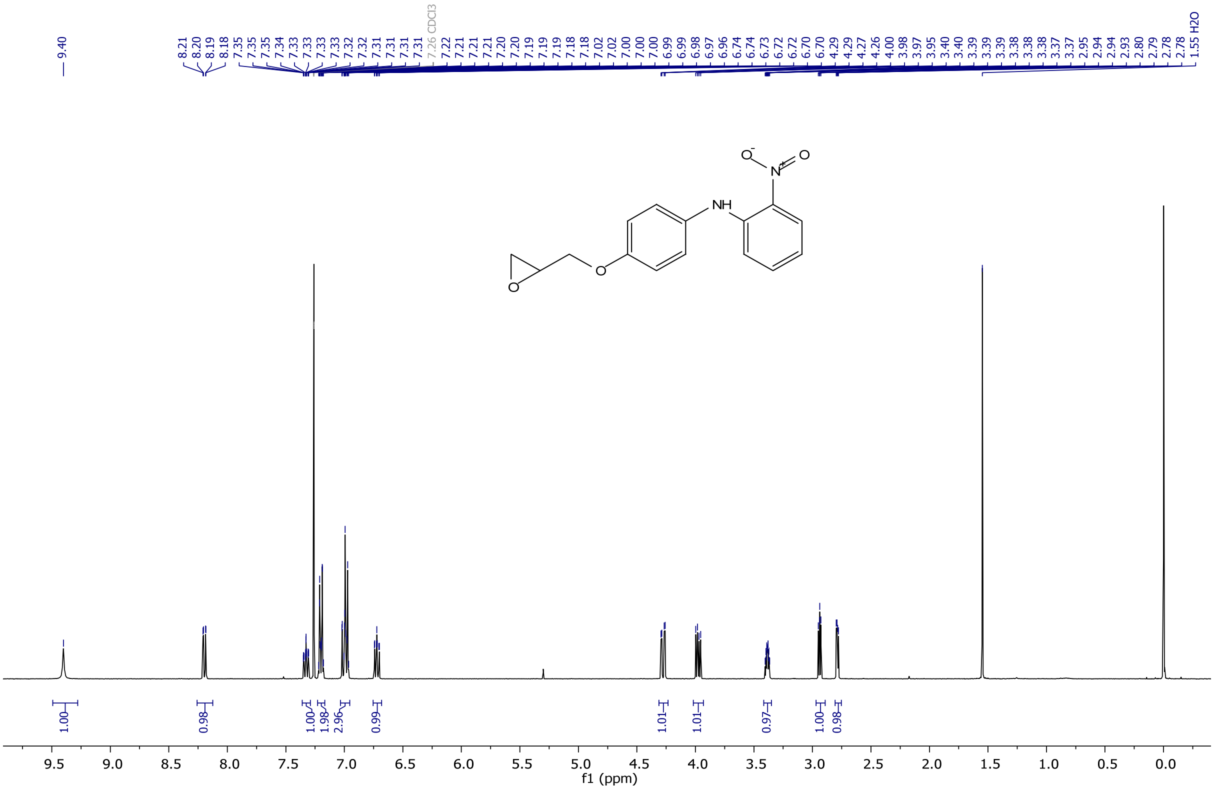

13C (101 MHz, CDCl3)

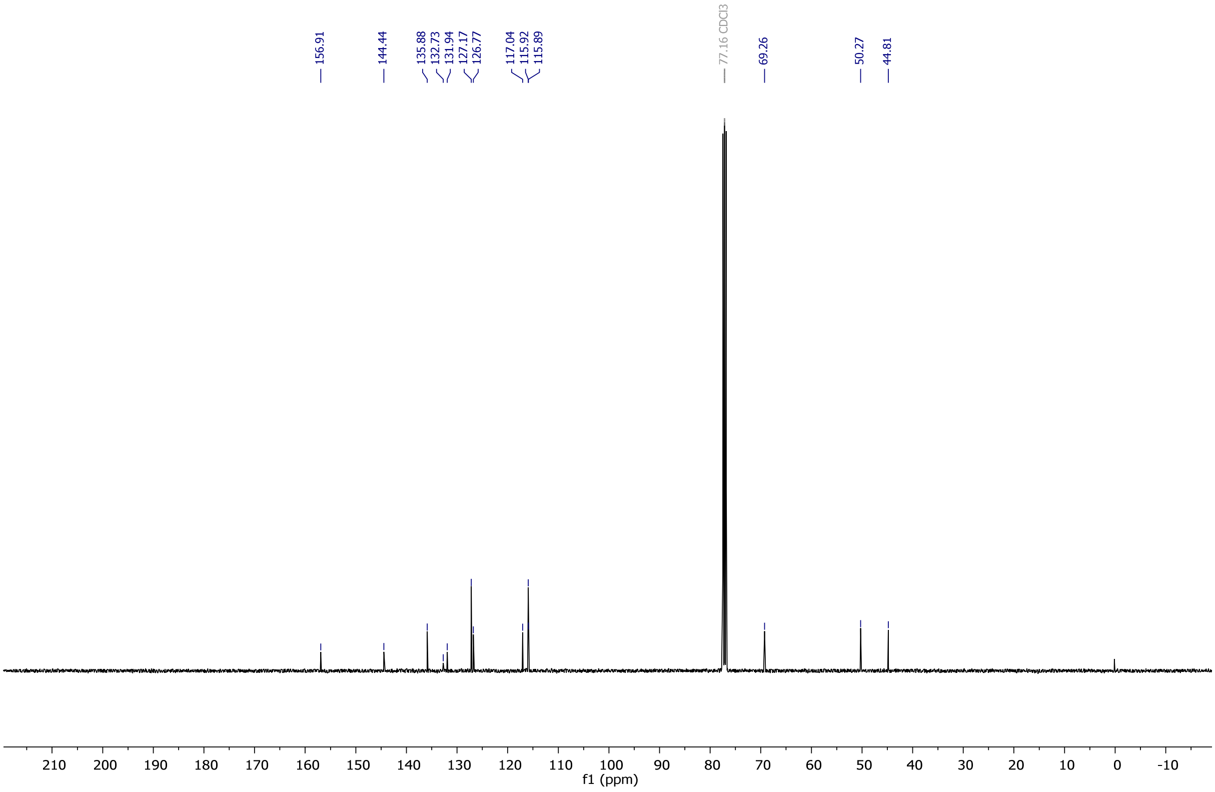

4-((4-(Trifluoromethyl)phenyl)amino)phenol (21)

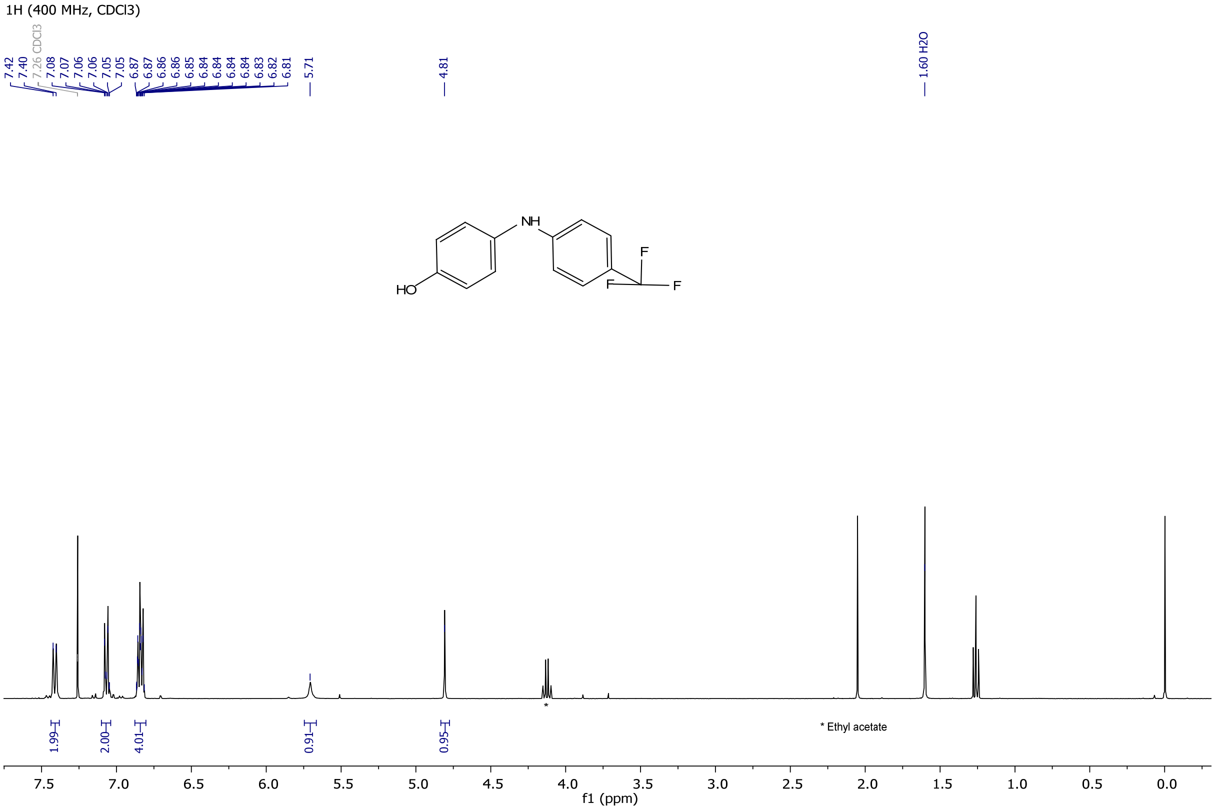

4-(Oxiran-2-ylmethoxy)-*N*-(4-(trifluoromethyl)phenyl)aniline (22)

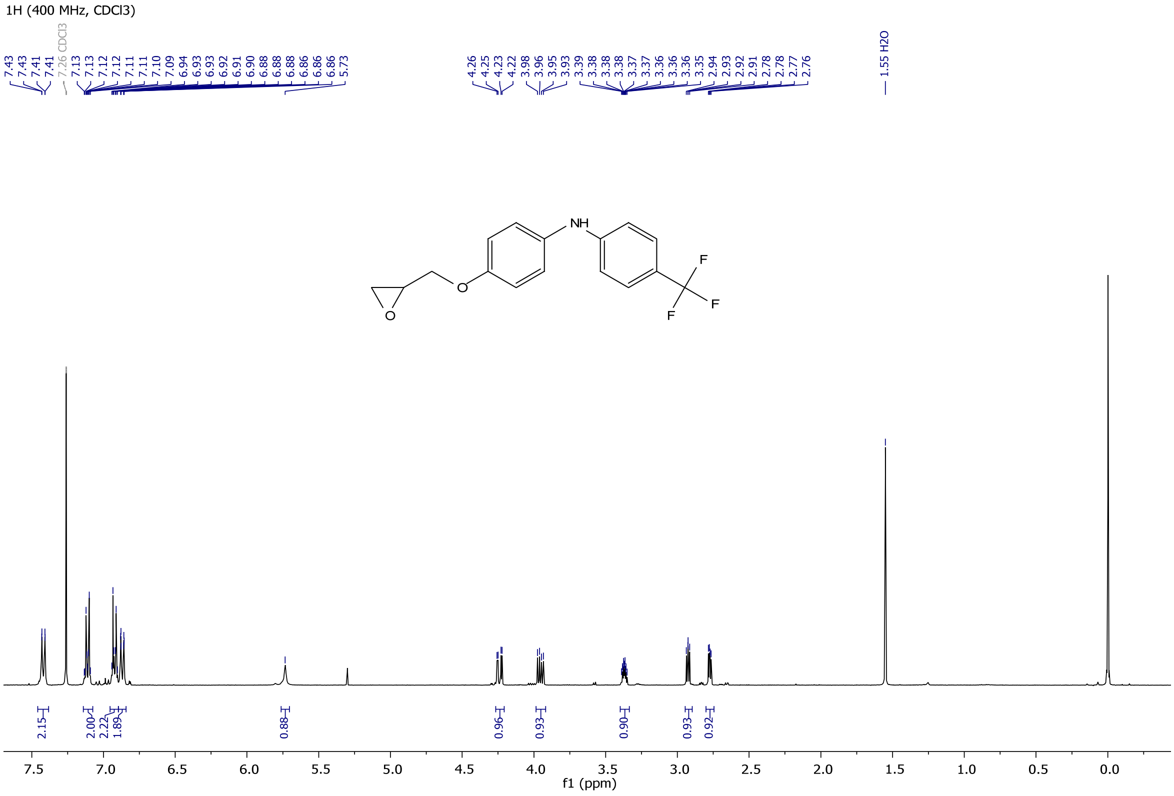

4-(Oxiran-2-ylmethoxy)-*N*-phenylaniline (24)

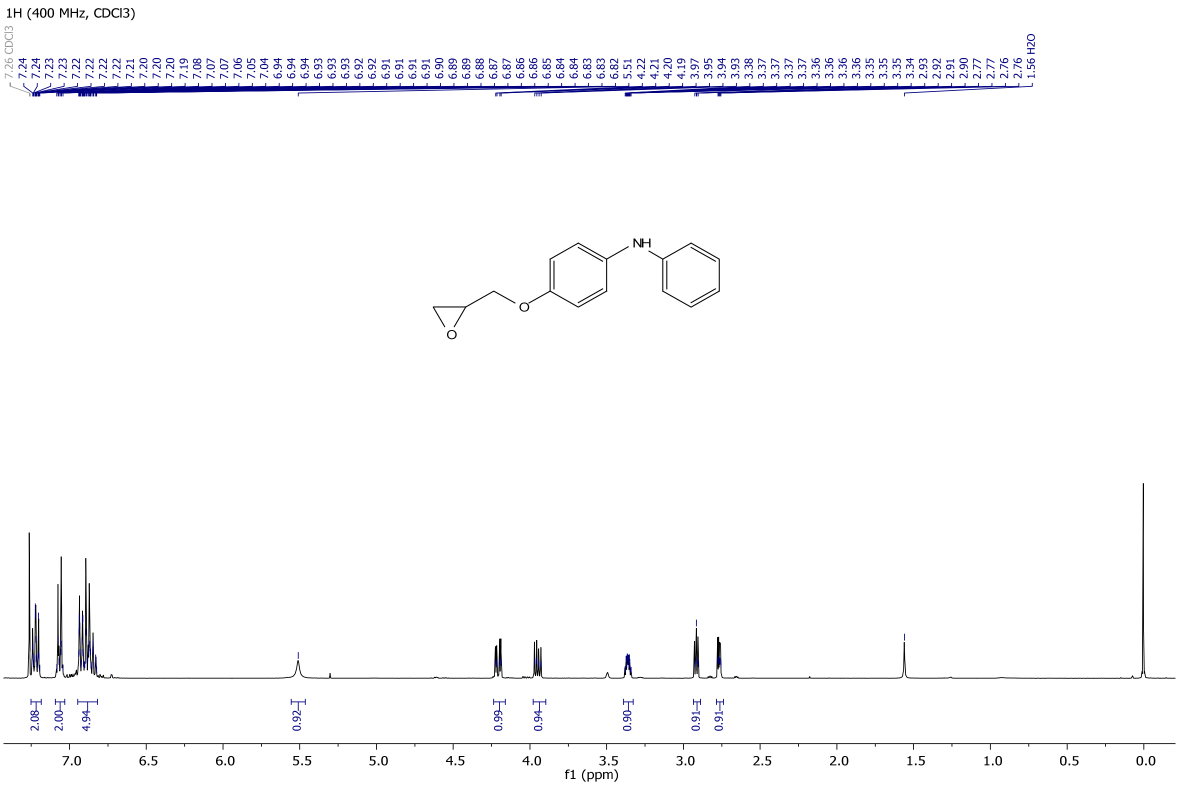

2-((4-hydroxyphenyl)amino)benzoic acid (26)

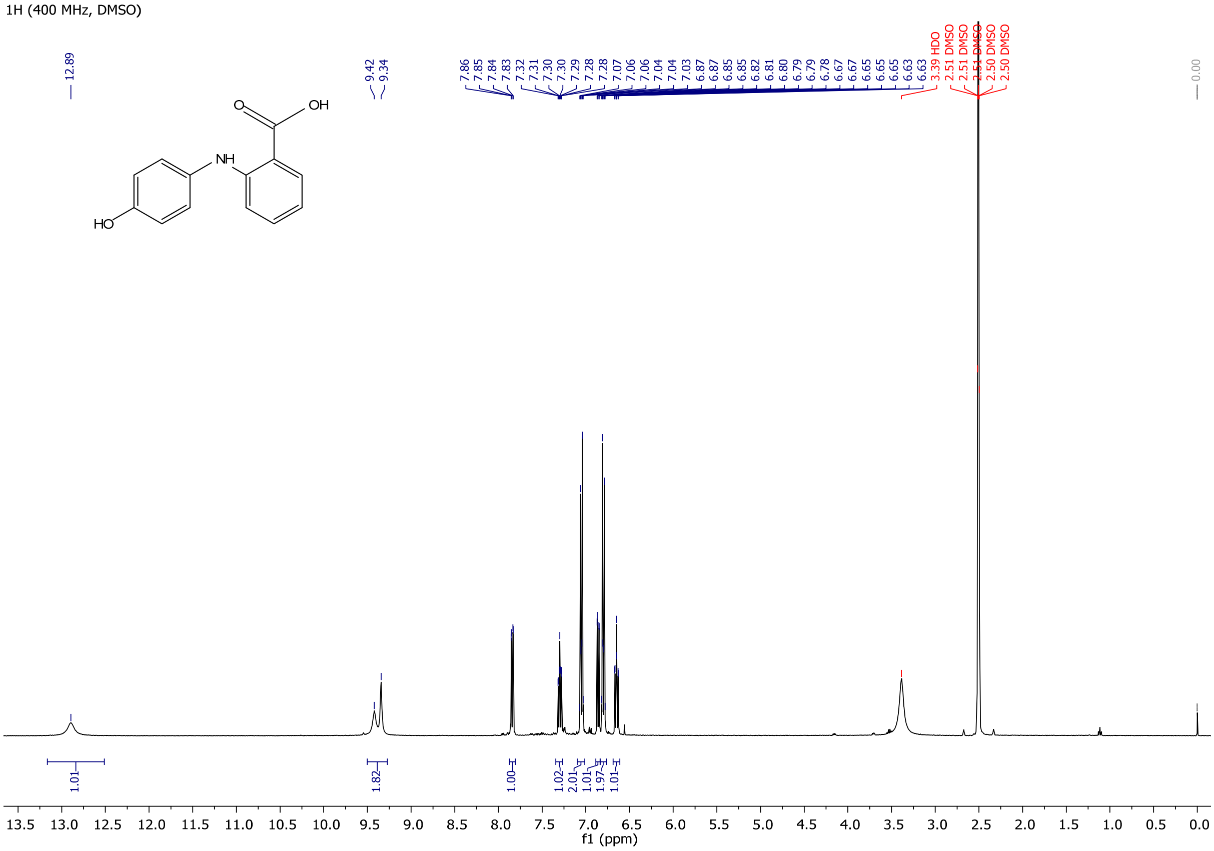

Methyl 2-((4-hydroxyphenyl)amino)benzoate (27)

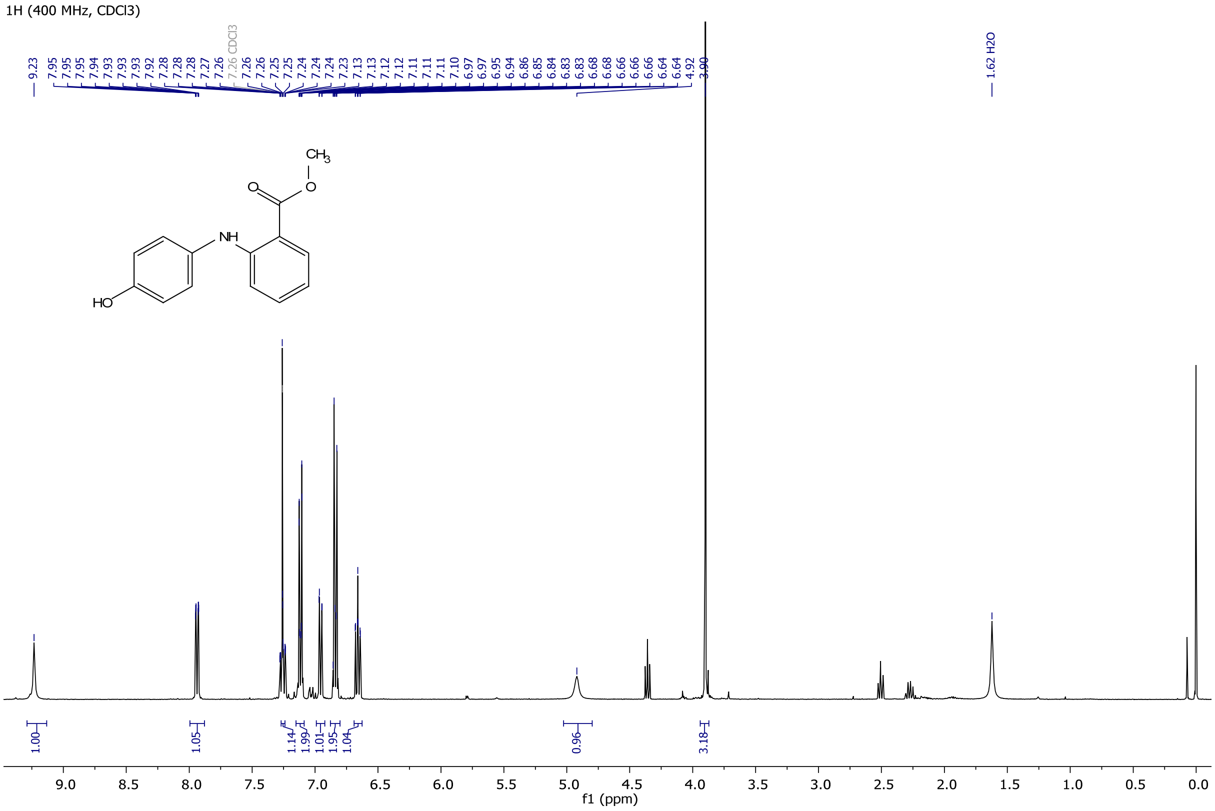

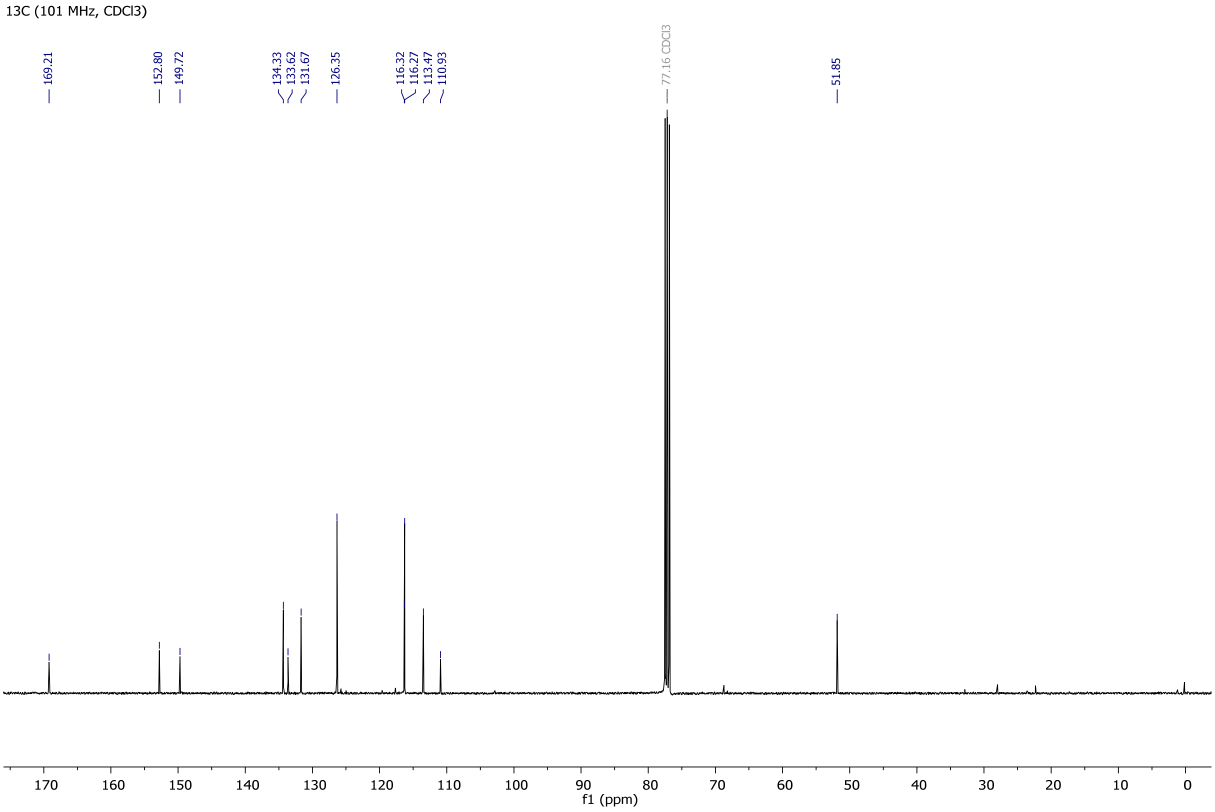

Methyl 2-((4-(oxiran-2-ylmethoxy)phenyl)amino)benzoate (28)

1H (400 MHz, CDCl3)

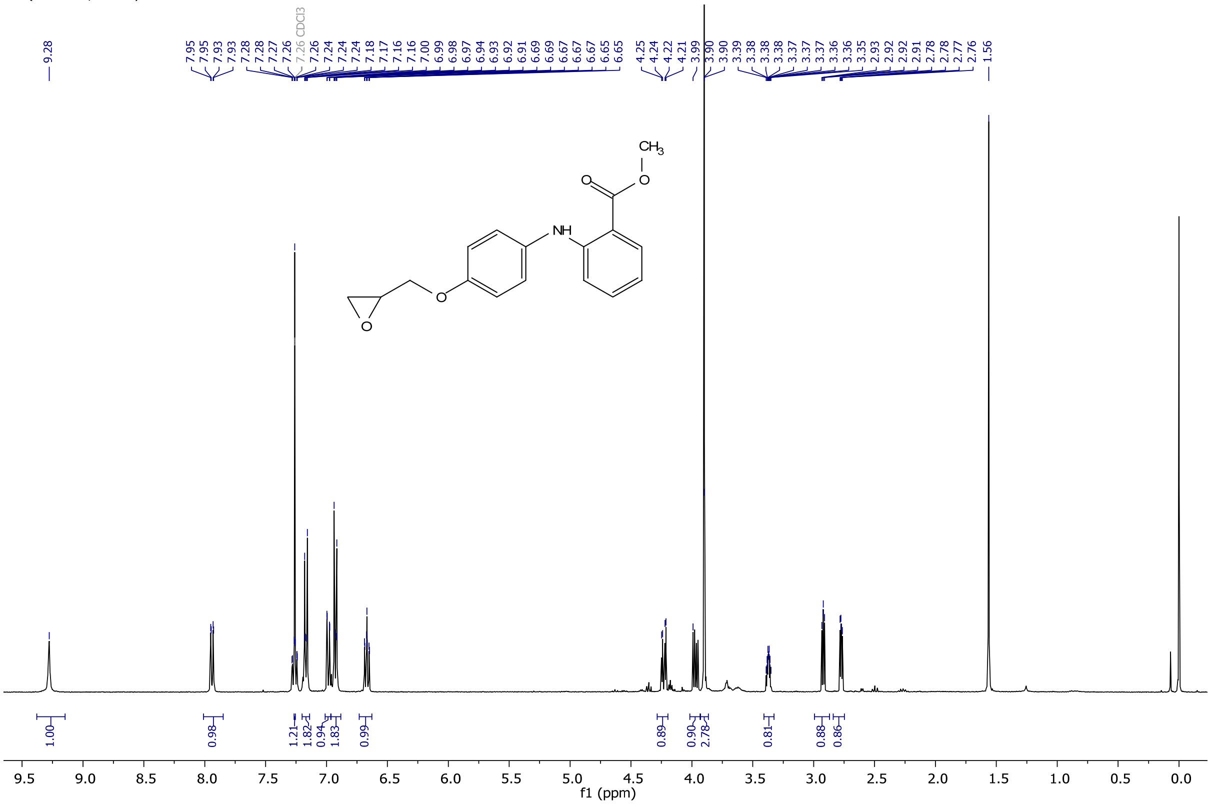

13C (101 MHz, CDCl3)

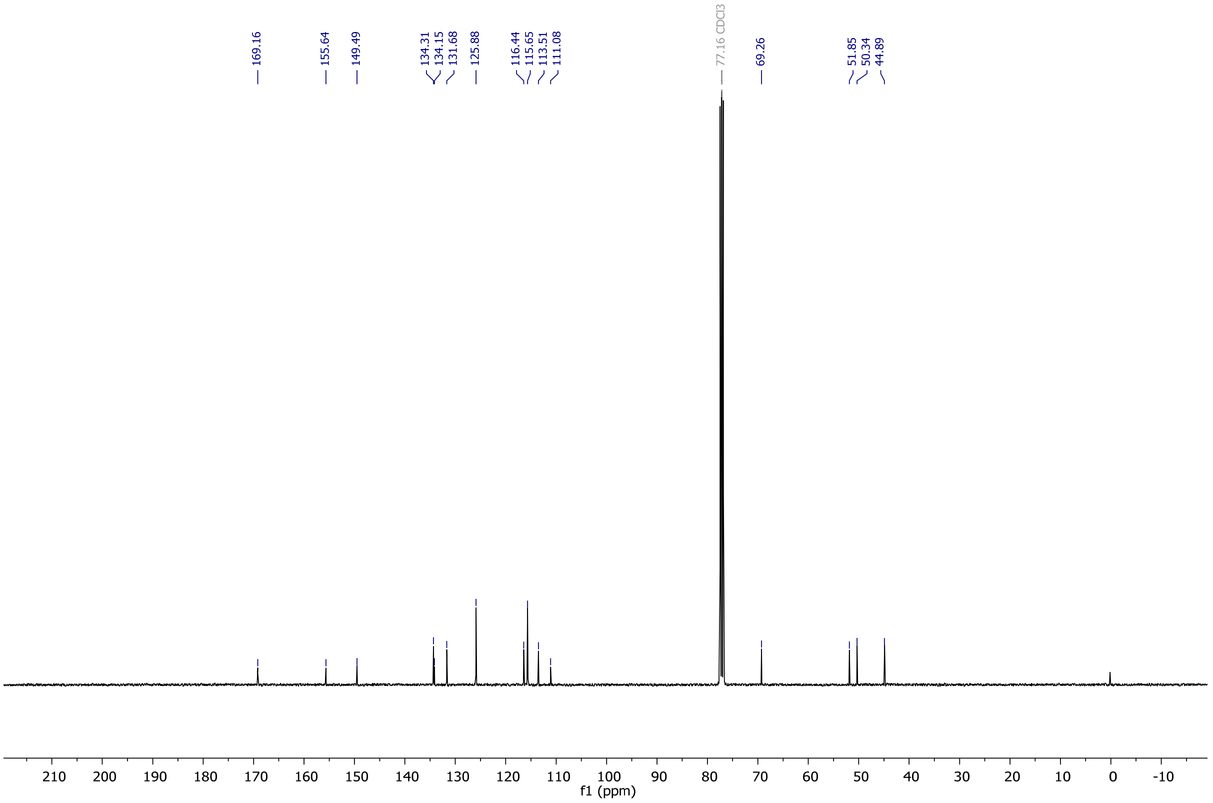

1-(Dimethylamino)-3-(4-((2-nitro-4-(trifluoromethyl)phenyl)amino)phenoxy)propan-2-ol (<u>ZVS-08</u>)

1H (400 MHz, CDCl3)

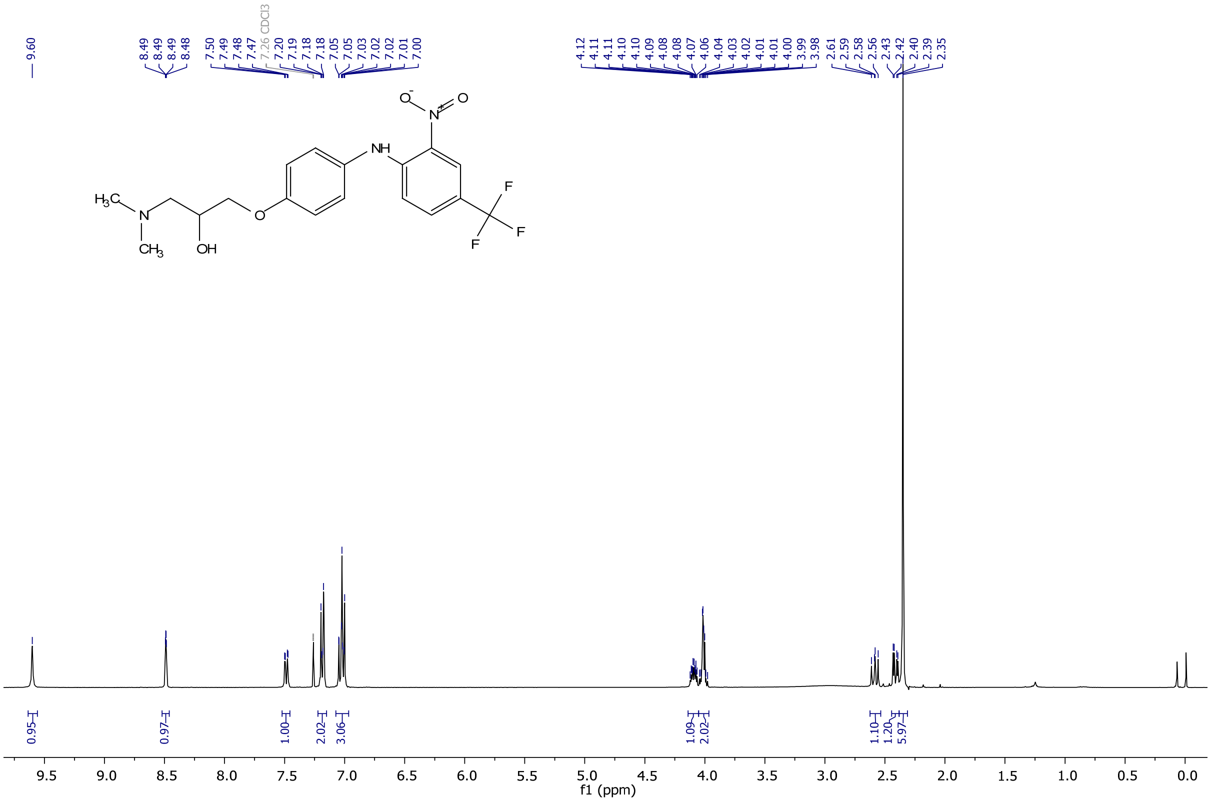

13C (100 MHz, CDCl3)

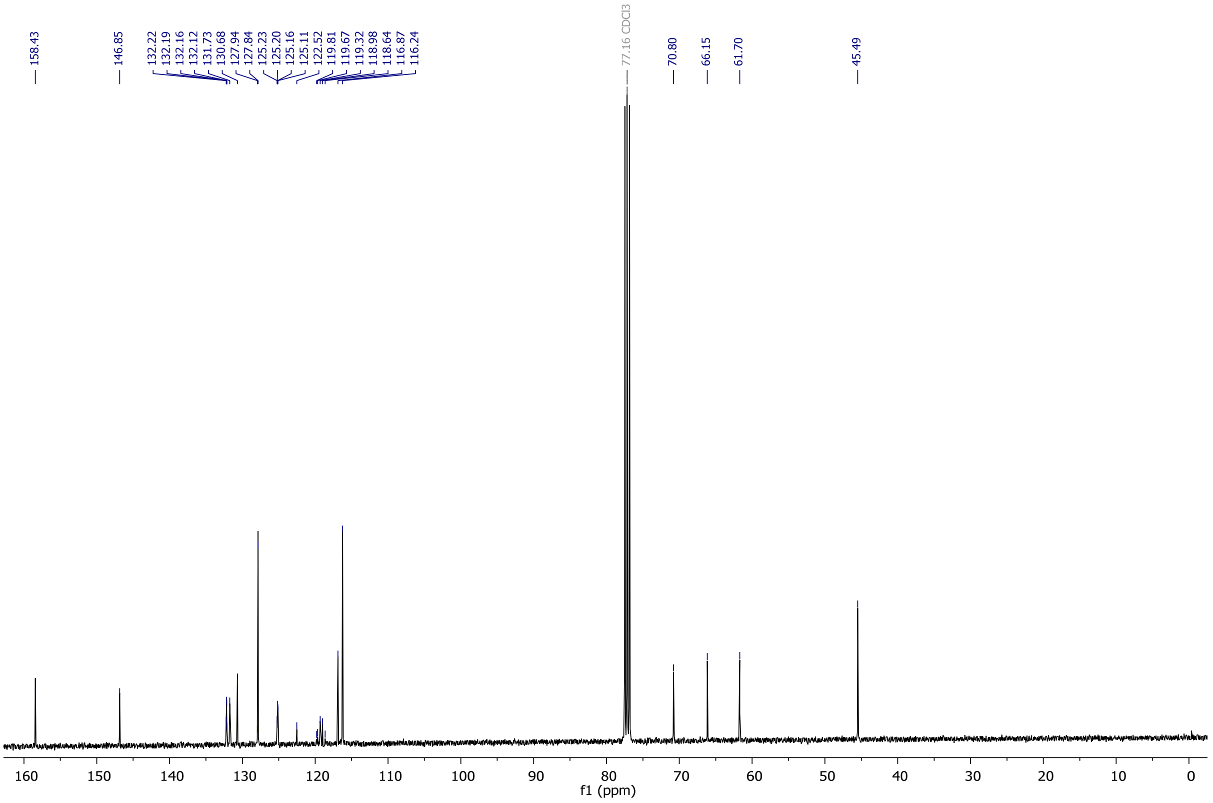

© 2021 by the authors. Submitted for possible open access publication under the terms and conditions of the Creative Commons Attribution (CC BY) license (http://creativecommons.org/licenses/by/4.0/).
